# Open access route and citation impact in orthopaedic research: A comparison of Gold/hybrid, Green, and Bronze open access within ten high-impact subscription-based journals

**DOI:** 10.1371/journal.pone.0360532

**Published:** 2026-11-04

**Authors:** William J. Daniels, Michelle Cruickshank, Taylor Dejong, Michelle Ghert

**Affiliations:** 1 University of British Columbia, Faculty of Medicine, Vancouver, British Colombia, Canada; 2 McMaster University, Michael G. DeGroote School of Medicine, Hamilton, Ontario, Canada; 3 McMaster University, Division of Orthopaedic Surgery, Hamilton, Ontario, Canada; 4 University of Maryland, Department of Orthopaedics, Baltimore, Maryland, United States of America; VIT University, INDIA

## Abstract

Open access (OA) publishing has expanded access to research, but citation patterns may differ across OA routes. We examined the association between OA route and citation rate within ten high-impact, predominantly subscription-based orthopaedic journals, analysing 24,115 articles published from 2014 to 2019. Journals in which more than 95% of articles were OA were excluded by design; fully OA journals were therefore not represented, and every Gold OA article in this corpus was published as hybrid OA within a subscription journal (hereafter Gold/hybrid OA). Articles were classified as Gold/hybrid OA, Green OA, Bronze OA, or Closed Access using Web of Science OA indicators. Results are reported in three layers: unadjusted descriptive comparisons, a primary adjusted analysis, andsensitivity analyses. The primary analysis used an entropy-balanced negative binomial generalized linear mixed model adjusting for publication year, document type, author count, and journal influence, with journal-level random effects. Most articles were Closed Access (78.5%), followed by Green OA (9.1%), Bronze OA (8.7%), and Gold/hybrid OA (3.8%). In the primary analysis, Gold/hybrid OA and Green OA had higher citation rates than Closed Access (rate ratio 1.41, 95% CI 1.30–1.54; and 1.35, 95% CI 1.28–1.43), and did not differ from each other (1.04, 95% CI 0.95–1.15). Citation rates varied by publication year: Gold/hybrid OA had the highest predicted rate in recent cohorts and Green OA in older cohorts with longer follow-up. Bronze OA had the lowest unadjusted citation counts, largely because it was enriched for lower-cited document types. The higher citation rates for Gold/hybrid and Green OA persisted across all sensitivity analyses. Within subscription-based orthopaedic journals, OA routes are not interchangeable; these findings do not extend to fully OA journals.

## Introduction

Open access (OA) publishing has transformed scientific communication by making research freely available to a global audience. Unlike traditional subscription-based (or Closed Access) models, OA eliminates financial barriers, and it is intended to broaden dissemination of knowledge across multiple disciplines, which may increase research visibility and impact [1]. In medicine, where timely access to new research is thought to influence patient care and clinical decision-making, OA plays an especially critical role in shaping the future outcomes in medical practice by accelerating knowledge translation [1,2].

OA publishing encompasses several models, often categorized by colour-coded labels based on accessibility and licensing. Gold OA refers to journals in which all articles are freely available immediately upon publication, with high article processing charges (APCs) that are typically paid by the author. Gold OA can occur either in fully OA journals, where all content is open, or as “hybrid” OA articles within subscription journals [3]; only the latter occurs in the present sample, and we therefore use the term Gold/hybrid OA throughout. Green OA involves authors self-archiving their work in institutional or subject-specific repositories, typically after an embargo period, often at no cost; although often they may have to follow more stringent guidelines depending on the journal [4]. Bronze OA refers to “free-to-read” articles that are made available on the publisher’s site without a clear open license and often at the publisher’s discretion, meaning access may be temporary and the underlying licensing status ambiguous [5].

Despite its advantages, OA publishing has raised questions regarding cost, transparency, and quality control. For instance, the cost of APCs for top journals has skyrocketed in recent years, with hyperinflated prices often outpacing inflation and journal influence [6,7]. High APCs may deter researchers in low- and middle-income countries from pursuing OA routes despite publisher discounts [8,9]. These costs may inadvertently reinforce the inequities in access that OA was intended to overcome [10,11].

Orthopaedic surgery is a rapidly evolving field, with research continually shaping surgical techniques, rehabilitation strategies, and patient outcomes. However, the adoption of OA publishing within orthopaedics remains inconsistent. A 2014 study by Sabharwal et al. analyzed 63 orthopaedic journals and revealed that 38 journals (60.3%) were fully Closed Access, offering no OA publishing options and restricting content to subscribers via institutional or personal access. Twenty journals (31.7%) operated under a hybrid model, allowing authors to make individual articles OA by paying a publication fee, while only five journals (8%) were fully OA, with all content freely available to readers [12]. This small proportion of fully OA journals likely limits the accessibility of orthopaedic research, particularly for clinicians, trainees, and researchers in low-resource settings who may not have access to institutional subscriptions. Lack of OA may also be associated with lower overall impact and citation counts [5].

Previous work assessing OA publishing in orthopaedic surgery has been limited by small sample sizes, narrow publication windows and lack of individual article analysis [12,13]. This study examines whether the route through which articles become freely readable is associated with citation rate among articles published in high-impact orthopaedic journals that publish predominantly through subscription or hybrid models. We focused on journals that publish both subscription and OA articles, allowing OA routes to be compared within the same journals while accounting for journal-level differences in readership, scope and citation practices. Journals publishing more than 95% of their content as OA were excluded by design; fully OA journals are therefore not represented, and the study does not evaluate OA publishing models in general.

## Materials and methods

### Journal identification

We identified the most highly ranked orthopaedic journals using a combination of SCImago Journal Rank (SJR), Journal Citation Reports (JCR) produced by Clarivate Analytics, and Article Influence Scores (AIS) as proxies for journal quality. SJR values were obtained from SCImago, which ranks journals using multiple citation-based indicators; Quartile Rankings and AIS were obtained from JCR. Quartile Rankings and AIS were obtained from SCImago journal reports, which ranks journals using multiple citation-based indicators. Quartile Rankings (Q1-Q4) divide journals within each subject category into four groups based on Impact Factor, with Q1 representing the top 25% of journals in the field. AIS reflects the average influence of a journal’s articles over their first five years of publication. The complete journal selection process is shown as a flow diagram in S1 Fig, and the per-journal screening log, giving the outcome and reason for every journal assessed, is provided as S1 Table.

Inclusion criteria for journals were: (1) classified under “Orthopaedics and Sports Medicine” in SJR; (2) ranked in JCR Quartile 1 (Q1); and (3) availability of article-level data in Web of Science. Journals were excluded if >95% of their articles were published as OA. This criterion was applied so that OA routes could be compared within the same journal rather than across journals, which reduces the risk of conflating journal-level characteristics with article-level access status; its consequence is that fully OA journals are not represented in the sample and that the Gold OA category consists entirely of hybrid OA articles. One additional influential journal, the Journal *of Bone and Joint Surgery* – American, was included following senior author recommendation on the grounds of its standing in the specialty; it also satisfied all three inclusion criteria independently. A total of 10 journals were included in the final analysis.

### Individual article metadata extraction

We used Web of Science (all databases) to extract article-level data and assess citation-based journal metrics. We focused on publications from 2014 to 2019 to allow for adequate accumulation of citations for bibliometric analysis and for an adequate number of papers per journal. For each article (n *=* 24,321), we extracted journal title, publication year, OA status, total citations, and author information. Articles that initially appeared as early online access but had official publication dates beyond our range (2020–2022) were excluded during data cleaning, as they did not meet the inclusion criteria of publication from 2014–2019 (n *=* 138), leaving (n *=* 24,183) articles for analysis. The Document Type variable encompassed several article types; however, article types with exceptionally low count (n *=* 1–32) were excluded, leaving (n *=* 24,115) articles for calculation and 6 unique document types. Author counts were determined by parsing the author field and counting semicolon-separated names in the author field. AIS values were obtained from the 2023 JCR journal reports and matched to articles by journal title. AIS is a journal-level metric reflecting the average influence of articles over the first five years following publication. AIS values were available for all included journals; therefore, no imputation or substitution was required. Year-specific AIS for each publication year (2014–2019) were not obtainable for all included journals from the available reports. A journal-by-publication-year random intercept was therefore added in a sensitivity analysis to absorb year-specific journal influence non-parametrically (see Statistical analysis). Finally, OA status was obtained from the Web of Science OA indicators. This field is multi-valued: a single article may carry more than one indicator. Articles were therefore assigned to one of four mutually exclusive categories using a fixed order of precedence, hybrid > Green > Bronze > none: Gold/hybrid OA (any hybrid indicator), Green OA (any Green indicator in the absence of a hybrid indicator), Bronze OA (a Bronze indicator alone), and Closed Access (no OA indicator). In this dataset, Gold OA corresponded exclusively to hybrid OA articles, and we therefore use the term Gold/hybrid OA throughout. Because journals in which more than 95% of articles were OA were excluded, fully OA journals did not contribute to the sample. Bronze OA comprised “Free-to-Read” articles that were free to read on the publisher’s site but lacked a specified open license. Green OA combined the “Green-submitted”, “Green-accepted”, and “Green-published” indicators and included self-archived versions of manuscripts in institutional or subject repositories; these three subtypes were retained separately and analysed individually as a sensitivity analysis, with records carrying more than one Green indicator assigned to the most final version (published > accepted > submitted). Definitions of each OA category, as classified in Web of Science, are given in Table 1 [14]. Two properties of this classification should be noted. First, the Web of Science OA field is a snapshot taken at the time of data extraction and carries no information on how long an article has been free to read; the duration and stability of Bronze access therefore could not be determined, and Bronze OA here denotes free-to-read status at extraction rather than a durable access arrangement. More broadly, these are database-derived access labels rather than homogeneous publishing strategies. Second, citation counts are cumulative totals at the date of extraction rather than counts accrued within a fixed window, so articles in earlier cohorts have had longer to accumulate citations than those published later; follow-up ranged from approximately five years for the 2019 cohort to approximately ten years for the 2014 cohort. Publication year is modelled explicitly and a citations-per-year specification is reported as a sensitivity analysis, but a standardized citation window could not be constructed because year-by-year citation counts were not available from the extract.

**Table 1:** Definitions of open access used in this study.

OA category	Subtype (where applicable)	Description*
Closed Access	–	No open license detected. Full text is only available via subscription or pay-per-view, and the article is not classified as OA in Web of Science/Unpaywall.
Gold/hybrid OA	Fully Gold journal — not represented in this study	Article is published in a journal where all content is OA and each article has a Creative Commons (CC) license recorded in Unpaywall; the journal meets the Budapest OA Initiative definition of Gold OA. Journals publishing more than 95% of their content as OA were excluded by design, so no article in this study falls into this subtype.
	Hybrid — all Gold OA articles in this study	Article has a CC license in Unpaywall but is published in a subscription or hybrid journal where only some articles are OA. Classification may be incomplete for newly published content.
Bronze OA	Free-to-Read	Article is free to read on the publisher’s site, but the license is unclear or non-CC. Access may be promotional or time-limited, and the licensing status may change over time. Web of Science records this status only at the time of data extraction, so the duration of free-to-read access cannot be determined.
Green OA	Published Version	Version of record deposited in an institutional or subject repository (e.g., an article in PubMed Central after any embargo period).
	Accepted Manuscript	Author-accepted manuscript deposited in a repository. The content has passed peer review but may not include the publisher’s copy-editing or typesetting.
	Submitted Manuscript	Original submitted version (preprint or pre–peer review manuscript) deposited in a repository prior to formal peer review.

OA: Open Access

*Descriptions adapted from Web of Science OA type documentation and materials [14].

### Statistical analysis

All analyses were performed using R version 4.4.2. The primary analysis employed a negative binomial generalized linear mixed model (GLMM) fitting using glmmTMB (v1.1.11) with emmeans (v1.11.0). Entropy balancing was implemented using WeightIt (v1.5.1) with balance diagnostics generated via cobalt (v4.6.1). Model performance metrics, including marginal and conditional R^2^ were calculated using MuMIn (v1.48.11), and overdispersion testing was conducted using performance (v0.13.0). Model coefficients were extracted using broom.mixed (v0.2.9.6).

### Analytic framework

Results are presented in three explicitly separated layers. Layer 1 comprises unadjusted descriptive comparisons: citation distributions, document-type composition, and non-parametric pairwise tests. These describe what is published under each OA label in this corpus and are not adjusted for confounding. Layer 2 is the primary adjusted analysis: a single entropy-balanced negative binomial GLMM, which is the source of every inferential conclusion reported in the Abstract and Conclusion. Layer 3 comprises sensitivity analyses that vary one modelling decision at a time around the Layer 2 model; these test the stability of the Layer 2 conclusions and are not used to generate new claims. Throughout, “citation rate” denotes the model-estimated expected number of citations for an article and “citation count” denotes observed totals; differences between OA categories are reported as rate ratios. Evaluative terms such as “advantage”, “benefit” and “outperform” are avoided so that a single vocabulary is used throughout.

### Descriptive comparisons of citation count by access type

Citation counts were summarized within each OA category using counts (n), means with standard deviations, medians with interquartile ranges (IQRs), and full ranges. Because citation distributions were strongly right-skewed and contained zeros, raw counts were visualized on a log10(x + 1)-transformed axis, so that zero citation articles are retained, using boxplots (Fig 1A). Pairwise comparisons of citation count between OA categories were conducted with two-sided Wilcoxon rank-sum tests, and p-values were adjusted for multiple comparisons using the Holm method. Median ratios (Group 1/ Group 2) were reported alongside adjusted p-values to convey the direction and magnitude of effects.

**Fig 1:**
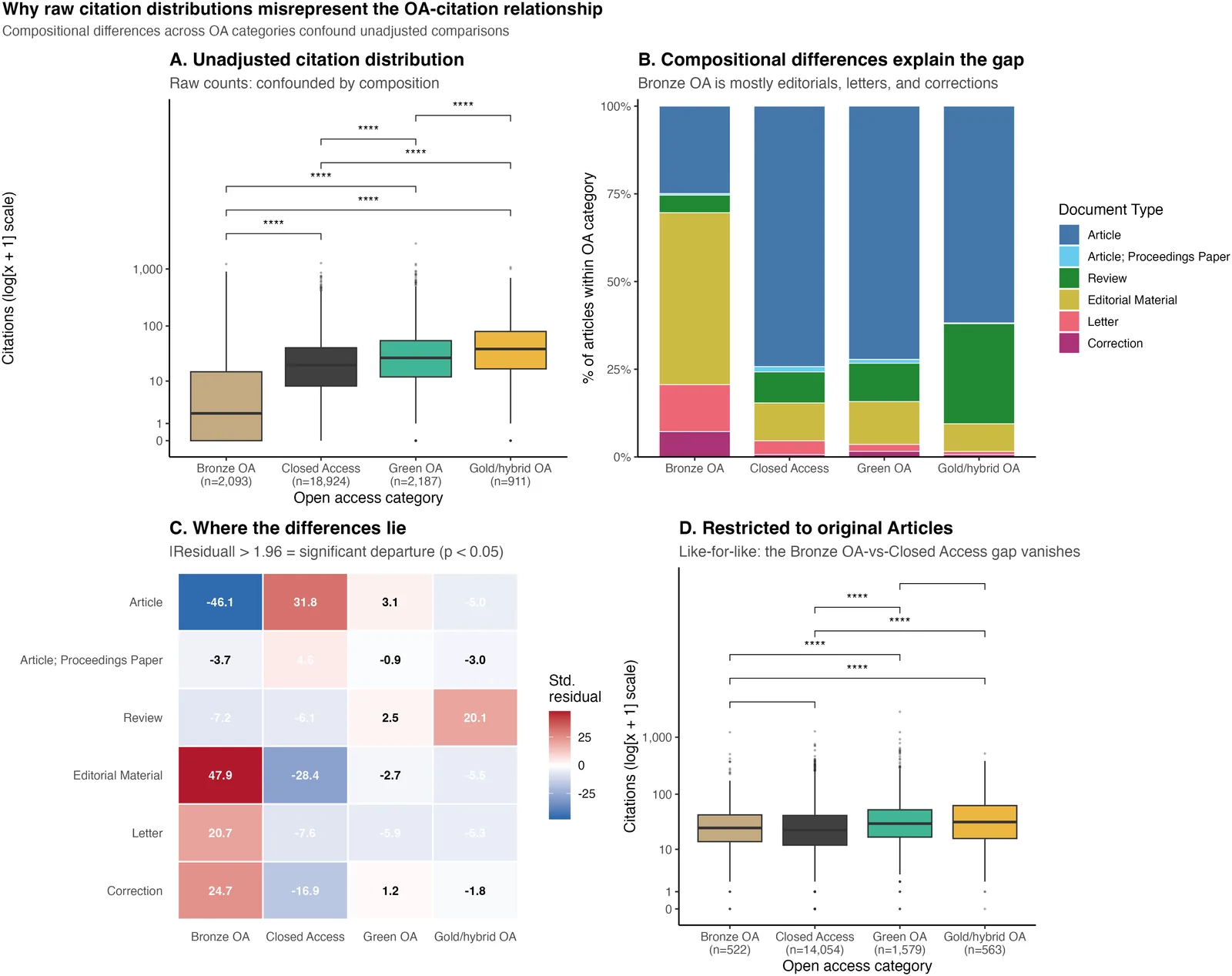
Compositional differences across open access categories explain the lower raw citation counts of Bronze OA. (A) Distribution of citation counts by OA category on a log10(x + 1) axis, so that zero citation articles can be shown. (B) Document-type composition within each OA category. (C) Standardized residuals from the chi-squared test of independence (χ^2^ = 4,142.1, df = 15, p < 0.001; Cramér’s V = 0.24). Red, enrichment; blue, depletion; |z| > 1.96 indicates departure from independence at α = 0.05. Bronze OA is enriched for Editorial Material (z = +47.9), Correction (+24.7), and Letter (+20.7), and depleted for Articles (−46.1); Closed Access shows the inverse. (D) Citations restricted to original Articles (n *=* 16,718); conventions as in (A). The Bronze OA versus Closed Access comparison, the most significant in (A) (median ratio 10.00, p ≈ 9 × 10 ^−^ ^296^), becomes non-significant (median ratio 0.92, p = 0.224); Gold/hybrid OA versus Green OA is likewise non-significant (p = 0.226). Bronze OA, brown; Closed Access, black; Green OA, green; Gold/hybrid OA, gold. **** p < 0.0001; unlabelled brackets, not significant.

### Document type composition analysis

To assess whether compositional differences in document types across OA categories might confound unadjusted comparisons of citation counts, we cross-tabulated document type (Article; “Article, Proceedings Paper”; Review; Editorial Material; Letter; Correction) by OA category (Bronze OA, Closed Access, Green, Gold/Hybrid OA) and tested for independence using Pearson’s chi-squared test. The strength of association was quantified using Cramér’s V, which ranges from 0 (independence) to 1 (perfect association); values of 0.1, 0.3, and 0.5 are conventionally interpreted as small, medium, and large effects, respectively. To identify which cells of the contingency table contributed most to the observed dependence, standardized (Pearson) residuals were computed; cells with absolute values exceeding 1.96 were considered to indicate departure from independence at the α = 0.05 level (two-sided).

### Primary model specification

The primary model used for inference in this study was an entropy-balanced negative binomial GLMM. To account for potential confounding variables inherent in observational bibliometric data, we employed entropy balancing targeting the Average Treatment Effect (ATE) estimate. This method reweights observations to ensure that covariate distributions (publication year, author count, AIS, and document type) are balanced across the four OA groups. Covariate balance was assessed using standardized mean differences; all covariates achieved balance below the threshold of 0.1 following weighting, indicating negligible residual imbalance.

### Model justification

Given the count nature of citation data, we initially considered a Poisson GLMM to analyze the relationship between OA type and citation counts. However, model comparison using likelihood ratio testing revealed significant overdispersion (χ^2^ = 561,206, df = 1, p < 2.2e-16), with the negative binomial model with log link function providing a substantially better fit (AIC = 202,772) compared to the Poisson model (AIC = 763,976).

We additionally evaluated whether zero-inflation required explicit modelling, given that 7.9% of articles overall received no citations (ranging from 1.3% for Gold/hybrid OA to 29.1% for Bronze OA). Comparison of the standard negative binomial model against a zero-inflated negative binomial model revealed no significant improvement in fit (χ^2^ = 0, df = 1, p = 1.0), indicating that the negative binomial distribution adequately captured the zero-citation articles without requiring a separate zero-inflation component.

### Model structure

The final model specification included fixed effects for OA type, standardized author count, standardized AIS, standardized publication year, and document type. An interaction term between publication year and OA type was included to assess how citation accumulation rates varied by access status. Random intercepts and random slopes for publication year were specified by the journal to account for journal-specific baseline citation rates and temporal accumulation patterns. The model explained approximately 53% of variance through fixed effects alone (marginal R^2^ = 0.530) and 61% when including random effects (conditional R^2^ = 0.609).

Between-journal variability in baseline citation rates was modest (σ^2^ = 0.031; standard deviation [SD] = 0.18), as was variability in citation accumulation over time (σ^2^ = 0.003; SD = 0.06). A strong negative correlation between intercepts and slopes (r = −0.87) indicated that journals with higher baseline citation rates exhibited attenuated citation growth over time.

Prior to analysis, continuous predictors were standardized to improve model convergence and facilitate interpretation of effect sizes. Author count (mean *=* 5.27, SD = 5.04) was standardized to analyze the effect of research collaboration on citation impact. AIS (mean *=* 1.80, SD = 1.04) was standardized to control for journal prestige effects. Publication year (mean *=* 2016.6, SD = 1.69) was standardized to account for temporal citation accumulation patterns, as more recent articles have less time to accumulate citations. Bronze OA served as the reference category for the model coefficients, allowing evaluation of the relative citation differences for Gold/hybrid OA (*n =* 911), Green OA (*n =* 2,187), and Closed Access publications (*n =* 18,924) compared to Bronze OA (*n =* 2,093); pairwise comparisons among all four categories were estimated separately, as described below.

To characterize how citation patterns differed across access types over time, we estimated marginal means for each OA type at each publication year (2014–2019) using the emmeans package, with pairwise comparisons adjusted via Tukey’s method. Because publication year was standardized and entered as a continuous predictor, articles from earlier cohorts (e.g., 2014) have had longer citation windows than those from later cohorts (e.g., 2019). The interaction between OA type and publication year, therefore captures whether certain access types accumulate citations at different rates as a function of citation window length. We note that because the model compares cumulative citation counts across publication-year cohorts rather than tracking individual articles longitudinally, inferences about within-article citation trajectories are indirect.

### Sensitivity and descriptive analyses

All Layer 2 inferential conclusions are based on the entropy-balanced negative binomial GLMM described above. Significance of fixed effects was evaluated using Type II Wald chi-square tests. Model coefficients were exponentiated to obtain incidence rate ratios (IRRs) with 95% confidence intervals for interpretability. Post hoc pairwise comparisons between OA categories were conducted using estimated marginal means on the response scale with Tukey adjustment for multiple comparisons; the reported confidence intervals carry the same Tukey adjustment as the accompanying *p*-values. For visualization of temporal trends and author effects, model predictions were generated at mean values of other covariates with 95% confidence intervals calculated from the variance-covariance matrix of fixed effects. All tests were two-tailed with α = 0.05. Five Layer 3 sensitivity analyses were specified. First, because entropy balancing can concentrate weight on a small number of observations, the full weight distribution, the maximum weight, the coefficient of variation and the effective sample size were summarized for each OA category, common support was assessed from generalized propensity scores estimated by multinomial logistic regression, and the primary model was refitted with weights truncated at the 99th, 97.5th and 95th percentiles and at the 1st and 99th percentiles (S2 Table; S2 Fig). Second, the three Green subtypes were entered as separate categories in a six-level treatment variable, re-weighted and refitted (S4 Table). Third, the model was refitted with an offset for the logarithm of years since publication, so that the outcome is the citation rate per year of follow-up rather than the cumulative count (S5 Table). Fourth, a journal-by-publication-year random intercept was added. Because any year-specific journal-level metric is by construction constant within a journal-year cell, this term absorbs year-specific journal influence non-parametrically, and is a more complete adjustment than substituting a year-specific value of a single journal metric (S5 Table). Fifth, the model was refitted without entropy-balancing weights so that the contribution of the weighting itself is visible. Unadjusted non-parametric comparisons are reported as Layer 1 descriptive analyses and are not used for inference. All specifications are compared side by side in S5 Table and S3 Fig. All data processing and statistical analysis code used to generate the results and figures in this manuscript are openly available at https://github.com/williamjdaniels/oa-citation-analysis.

## Results

### Sample characteristics

The final dataset comprised 24,115 articles from 10 orthopaedic surgery journals published between 2014 and 2019. The number of articles per journal averaged 2,412 and ranged from 1,046–3,901. Most articles were Closed Access (78.5%, *n =* 18,924), followed by Green OA (9.1%, *n =* 2,187), Bronze OA (8.7%, *n =* 2,093), and Gold/hybrid OA (3.8%, *n =* 911).

### Weighting, covariate balance, and effective sample size

Raw citation counts showed substantial right skew. Unadjusted median citations range from 2 (Bronze OA) to 39 (Gold/hybrid OA). Entropy balancing achieved exact covariate balance across all treatment groups, with all standardized mean differences reduced to below 0.001 after weighting. This balance came at the cost of reduced effective sample sizes, particularly for Gold/hybrid OA (reduced from 911 to 386, a 58% reduction) and Bronze OA (reduced from 2,093–925, a 56% reduction). Green OA retained 65% of its effective sample size (1,431 of 2,187), while Closed Access articles retained 97% (18,343 of 18,924), reflecting this group’s representativeness of the overall covariate distribution. Zero citations were most common among Bronze OA articles (29.1%) and least common among Gold/hybrid OA articles (1.3%). Articles averaged 5.27 authors (SD = 5.04); mean author count was lowest for Bronze OA (3.39) and highest for Green OA (7.06), with Closed Access (5.25) and Gold/hybrid OA (5.64) intermediate. The distribution of author counts was highly skewed, with most articles having 5–8 authors (n = 12,716), followed by 3–4 authors (n = 5,726), a single author (n = 2,095), 9–32 authors (n = 1,825) and 2 authors (n = 1,648). Sixty records had no named author, and 45 articles had more than 32 authors. The full weight distribution, including maximum.

OA distribution varied substantially by journal. For instance, *Sports Medicine* had the highest percentage of Gold/hybrid OA articles (20.5%), while *Arthroscopy* had the greatest proportion of Bronze OA (40.7%). In contrast, the *Spine Journal* and *Journal of Shoulder and Elbow Surgery* published more than 90% of their articles as Closed Access. Gold/hybrid OA articles were also published in journals with a higher average AIS, with a mean AIS of 2.69 compared to 1.70 for Bronze OA and 1.69 for Closed Access. Additional details on OA distribution, citation metrics, and journal-level characteristics are presented in Table 2.

**Table 2:** Article counts, citation metrics, and open access distribution by journal (2014-2019).

Journal	N	Mean Citations	Median Citations	AIS	Bronze OA (%)	Closed (%)	Green OA (%)	Gold/hybrid OA (%)
*Journal of Arthroplasty*	3,901	28.85	19	1.18	5.5	87.0	6.1	1.4
*Knee Surgery, Sports Traumatology, Arthroscopy*	3,042	26.89	19	1.09	4.1	81.4	7.3	7.2
*Arthroscopy – Journal of Arthroscopic and Related Surgery*	2,697	21.58	12	1.36	40.7	56.6	2.2	0.4
*American Journal of Sports Medicine*	2,542	48.44	32	1.82	6.9	80.6	11.8	0.6
*Spine Journal*	2,395	22.17	12	1.47	0.7	90.9	6.7	1.7
*Journal of Bone and Joint Surgery – American*	2,391	34.43	20	2.06	7.7	78.8	12.8	0.7
*British Journal of Sports Medicine*	2,214	52.79	20	4.63	6.5	54.9	27.4	11.1
*Journal of Shoulder and Elbow Surgery*	2,148	25.54	18	0.89	1.8	91.8	5.4	1.1
*Bone and Joint Journal*	1,739	32.34	22	1.66	2.9	86.0	7.2	3.9
*Sports Medicine*	1,046	81.27	52	3.41	4.6	70.0	5.0	20.5

A summary of the data used in this study, including the number of journals analyzed, total articles included (*n =* 24,115), and journal-level metrics such as mean/median citation counts and AIS. The table also reports the proportion of articles in each OA category for every journal included in the final dataset.

Abbreviations: AIS: Article Influence Score; OA: open access

### Layer 1: unadjusted descriptive findings

#### Unadjusted citation counts differ markedly across OA categories.

In the full analysis sample (n *=* 24,115), unadjusted citation counts varied substantially across OA categories (Fig 1A; Table 4). Bronze OA articles had the lowest median citation count (median 2, IQR 0–15, *n =* 2,093), followed by Closed Access (median 20, IQR 8–41, *n =* 18,924), Green OA (median 27, IQR 12–55, *n =* 2,187), and Gold/hybrid OA (median 39, IQR 17–80, *n =* 911). All pairwise comparisons between OA categories were statistically significant (Wilcoxon rank-sum tests, all Holm-adjusted *p* < 0.001; Table 4). Most strikingly, the median citation count for Closed Access was ten times higher than that for Bronze OA (median ratio 10.00, p ≈ 9 × 10 − 296), suggesting at face value substantially lower citation counts for Bronze OA articles relative to subscription publication. These are unadjusted descriptive comparisons and are not used for inference.

### Document type composition differs strongly across OA categories

To explore the striking differences in citation count between OA categories, document type was investigated, which was significantly associated with OA category (Pearson’s chi-squared test: *χ2* = 4,142.1, *df* = 15, *p* < 0.001; Cramér’s *V* = 0.24, indicating a small-to-medium effect). Bronze entries comprised 24.9% original Articles, 49.0% Editorial Material, 13.4% Letters, 7.2% Corrections, 5.0% Reviews, and 0.4% Article; Proceedings Paper. By contrast, Closed Access entries were dominated by original Articles (74.3%), with Editorial Material (10.8%), Reviews (8.8%), Letters (3.9%), Article; Proceedings Paper (1.5%), and Corrections (0.7%) making up the remainder. Gold/hybrid OA and Green OA showed compositions intermediate between these extremes, with Gold/hybrid OA notably enriched for Reviews (28.5% vs. 5.0%–10.9% in the other categories) (Fig 1B; S3 Table).

Standardized residuals localized these differences (Fig 1C; S3 Table). Bronze OA was strongly enriched for Editorial Material (z = +47.9), Correction (z = +24.7), and Letter (z = +20.7), and strongly depleted for original Articles (z = −46.1). Closed Access showed the inverse pattern, with enrichment for Articles (z = +31.8) and depletion for Editorial Material (z = −28.4) and Corrections (z = −16.9). Gold/hybrid OA was uniquely enriched for Reviews (z = +20.1). All residuals reported here exceeded the two-sided α = 0.05 threshold of |z| > 1.96. In aggregate, non-research items (editorial materials, letters, and corrections combined) accounted for approximately 70% of Bronze OA entries but only 15% of Closed Access entries. Because non-research items are systematically less cited than original research, this compositional imbalance is sufficient to generate the raw Bronze OA versus Closed Access citation gap without requiring difference attributable to access route.

#### Restricting to original articles removes the apparent Bronze OA difference.

When the analysis was restricted to original Articles only (n *=* 16,718; Bronze OA *n =* 522, Closed Access *n =* 14,054, Green OA *n =* 1,579, Gold/hybrid OA *n =* 563), the apparent Bronze OA difference disappeared: Bronze OA and Closed Access no longer differed (median ratio 0.92, Holm-adjusted p = 0.224), while Green OA and Gold/hybrid OA remained higher than Closed Access (Table 3; Table 4). The Bronze OA median rose from 2 to 25 (IQR 14–43), the Closed Access median rose from 20 to 23 (IQR 12–42), and the Bronze-to-Closed median ratio inverted from 10.00 to 0.92. The corresponding pairwise Wilcoxon test, which had been the most highly significant comparison in the unadjusted analysis (p ≈ 9 × 10 − 296), was not significant in the Articles-only subset (Holm-adjusted p = 0.224). The Gold/hybrid OA versus Green OA comparison was likewise no longer significant (median ratio 1.07; p = 0.226). Comparisons involving Green OA and Gold/hybrid OA against Bronze OA and Closed Access remained statistically significant in the Articles-only analysis, but were substantially attenuated, with median ratios of 1.20–1.39 compared with 1.35–19.50 in the unadjusted analysis (Table 4).

**Table 3:** Unadjusted citation counts restricted to original articles only, by open access category.

Open Access Category	*n*	Mean Citations	Median [Q1–Q3]
Bronze OA	522	40.19	25 [14–43]
Closed Access	14,054	33.82	23 [12–42]
Green OA	1,579	50.69	30 [17–53]
Gold/hybrid OA	563	48.72	32 [16–63]

**Table 4:** Pairwise wilcoxon rank-sum comparisons of unadjusted citation counts in the full sample and the articles-only subset.

Subset	Comparison	Median 1	Median 2	Median Ratio	Holm-adjusted p
All articles (raw)	Closed Access vs. Bronze OA	20	2	10.00	< 0.001
All articles (raw)	Green OA vs. Bronze OA	27	2	13.50	< 0.001
All articles (raw)	Gold/hybrid OA vs. Bronze OA	39	2	19.50	< 0.001
All articles (raw)	Green OA vs. Closed Access	27	20	1.35	< 0.001
All articles (raw)	Gold/hybrid OA vs. Closed Access	39	20	1.95	< 0.001
All articles (raw)	Gold/hybrid OA vs. Green OA	39	27	1.44	< 0.001
Articles only	Closed Access vs. Bronze OA	23	25	0.92	0.224
Articles only	Green OA vs. Bronze OA	30	25	1.20	< 0.001
Articles only	Gold/hybrid OA vs. Bronze OA	32	25	1.28	< 0.001
Articles only	Green OA vs. Closed Access	30	23	1.30	< 0.001
Articles only	Gold/hybrid OA vs. Closed Access	32	23	1.39	< 0.001
Articles only	Gold/hybrid OA vs. Green OA	32	30	1.07	0.226

#### Reconciliation of the unadjusted and adjusted findings.

These Layer 1 descriptive findings are consistent with the Layer 2 adjusted model reported below. After adjustment for document type, publication year, author count, and journal AIS, the large unadjusted difference between Bronze OA and Closed Access was no longer present; they showed equivalent citation rates in the 2014 cohort (rate ratio 1.00, 95% CI CI 0.89–1.13, p > 0.99) and a modest difference favouring Bronze OA by the 2019 cohort (rate ratio 1.18, 95% CI 1.06–1.32, *p* < 0.001). This residual difference was small and sensitive to the weighting specification (see Layer 3), and no Bronze OA versus Closed Access difference was present in the Articles-only analysis. Both analytical approaches therefore indicate that the lower unadjusted citation counts of Bronze OA primarily reflect compositional differences across access categories — particularly the predominance of editorials, letters, and corrections among Bronze OA entries — rather than access route. By contrast, the higher citation counts of Gold/hybrid OA and Green OA relative to Closed Access persisted in the Articles-only analysis, and their higher citation rates persisted after adjustment. The unadjusted boxplot (Fig 1A) is therefore best interpreted as a description of what is published under each access label in this corpus, not as an estimate of a citation difference attributable to open access itself.

Because the unadjusted gradient and the adjusted estimates pointed in different directions for Bronze OA, we examined whether the unadjusted gradient reflected systematic differences in the types of items appearing under each access label rather than a difference in citation rate.

### Layer 2: primary adjusted analysis

#### Primary model results.

The weighted negative binomial GLMM provided an excellent fit to the data, explaining 53.0% of the variance associated with fixed effects (Marginal R^2 = 0.530) and 60.9% of the total variance including random effects (Conditional R^2 = 0.609). A likelihood ratio test confirmed that the negative binomial model significantly outperformed a Poisson specification (χ^2^ = 561,206, p < 0.001), indicating strong overdispersion in the citation data. Diagnostics further confirmed that the negative binomial specification adequately handled this overdispersion (Dispersion ratio = 1.10, p = 0.304).

A zero-inflated negative binomial model was also evaluated to assess whether excess zeros required separate modelling. The zero-inflated specification did not improve model fit (χ^2^ = 0, df = 1, p = 1.0), indicating that the standard negative binomial distribution adequately captured the observed zero-citation articles without requiring a separate zero-generating process.

The model included random intercepts and random slopes for publication year across journals to account for between-journal heterogeneity in both baseline citation rates and temporal trends. Between-journal variability in baseline citation rates was modest (σ^2^ = 0.031, SD = 0.18), as was variability in citation accumulation over time (σ^2^ = 0.003, SD = 0.06). A strong negative correlation between random intercepts and slopes (r = −0.87) indicated that journals with higher baseline citation rates showed attenuated citation growth over time, consistent with a regression-to-the-mean or ceiling effect among high-impact journals.

Type II Wald chi-square tests indicated that all predictors were statistically significant (Table 5). Document type contributed most to model fit (χ^2^ = 13,431.0, df = 5, p < 0.001), followed by author count (χ^2^ = 298.3, df = 1, p < 0.001), OA category (χ^2^ = 262.0, df = 3, p < 0.001), the interaction between OA category and year (χ^2^ = 222.6, df = 3, p < 0.001), publication year (χ^2^ = 126.7, df = 1, p < 0.001), and AIS (χ^2^ = 94.3, df = 1, p < 0.001). After adjusting for journal influence, author collaboration, document type, and publication age, and evaluated at the mean publication year (the model includes an OA category × year interaction), Gold/hybrid OA and Green OA had higher citation rates than Bronze OA, while Closed Access had a slightly lower citation rate than Bronze OA. Gold/hybrid OA articles had a citation rate 1.30 times that of Bronze OA (Estimate = 0.26, p < 0.001). Green OA articles had a citation rate 1.24 times that of Bronze OA (Estimate = 0.22, p < 0.001). Closed Access articles had a citation rate 0.92 times that of Bronze OA (Estimate = −0.08, p < 0.001), an 8% lower citation rate relative to Bronze OA; this small difference was not robust across model specifications (see Layer 3).

**Table 5:** Predictors of citation count from negative binomial regression.

Variable	χ^2^	df	p-value
Open access category	261.97	3	< 0.001
Author Count	298.31	1	< 0.001
Article Influence Score	94.30	1	< 0.001
Publication Year	126.66	1	< 0.001
Document Type	13,430.97	5	< 0.001
Open access category × Year	222.457	3	< 0.001

Summary of model results examining the relationship between citation count and six model terms: open access category, author count, AIS, publication year, document type, and the interaction between open access category and year (Type II Wald chi-square tests).. All terms were statistically significant predictors of citation count.

### Author count, journal influence, and document type

Author count was positively associated with citation rate. For every standard deviation increase in author count, the citation rate increased by 23.3% (Estimate = 0.21, p < 0.001). Similarly, journal influence was a strong predictor; a one standard deviation increase in AIS was associated with a 40.7% increase in citation rate (Estimate = 0.34, p < 0.001).

Document type heavily influenced citation frequency. Compared to standard Articles (reference), Reviews had a significantly higher citation rate (Estimate = 0.52, p < 0.001), while Corrections (Estimate = −3.69), Letters (Estimate = −2.41), and Editorial Materials (Estimate = −1.85) gad markedly lower citation rates.

### Temporal patterns and interaction

The publication-year coefficient, which is the slope for the reference category (Bronze OA), was negative (Estimate = −0.14, p < 0.001), consistent with older articles having had more time to accumulate citations (the “citation window” effect).However, the significant interaction between OA category and year (χ^2^ = 222.6, p < 0.001) indicates that the association between OA category and predicted citation rate differed by publication cohort.

Using Bronze OA as the reference category, Green OA showed a significantly steeper decline in predicted citations for more recent years compared to Bronze OA (Interaction Estimate = −0.38, p < 0.001). Gold/hybrid OA also showed a steeper decline compared to Bronze OA (Interaction Estimate = −0.09, p = 0.027). Closed Access showed a slightly steeper decline compared to Bronze OA (Interaction Estimate = −0.06, p = 0.027). Differences between OA categories were therefore more pronounced in older cohorts with longer follow-up, and more compressed in recent cohorts.

For recent publication cohorts with shorter follow-up, Gold/hybrid OA had the highest predicted citation counts, consistent with the immediate visibility conferred by unrestricted access at publication. In the 2019 cohort (~5 years of accumulation), Gold/hybrid OA articles had a predicted mean of 9.65 citations (95% CI: 8.45–11.03), compared to 8.46 for Bronze OA (7.55–9.47), 7.15 for Closed Access (6.60–7.76), and 6.12 for Green OA (5.51–6.80). In this cohort the Gold/hybrid OA citation rate was 58% higher than that of Green OA (Green/Gold ratio = 0.63, z = −6.91, p < 0.001), and the Green OA rate was also below that of Closed Access (Closed/Green ratio = 1.17, z = 4.13, p = 0.0002).

For older cohorts with longer follow-up, this ordering reversed. In the 2014 cohort (~10 years of accumulation), Green OA had the highest predicted citations at 27.90 (95% CI: 23.32–33.38), substantially exceeding Gold/hybrid OA at 18.80 (15.42–22.92; Green/Gold ratio = 1.48, z = 5.68, p < 0.001). Green OA also exceeded Closed Access (12.69; Closed/Green ratio = 0.45, p < 0.001) and Bronze OA (12.66). The ordering of Green OA and Gold/hybrid OA changed around 2016–2017: in 2016, Green OA and Gold/hybrid OA did not differ significantly (ratio = 1.06, p = 0.54), but by 2017 the Gold/hybrid OA rate exceeded the Green OA rate (ratio = 0.89, z = −2.92, p = 0.018) (Fig 2).

**Fig 2:**
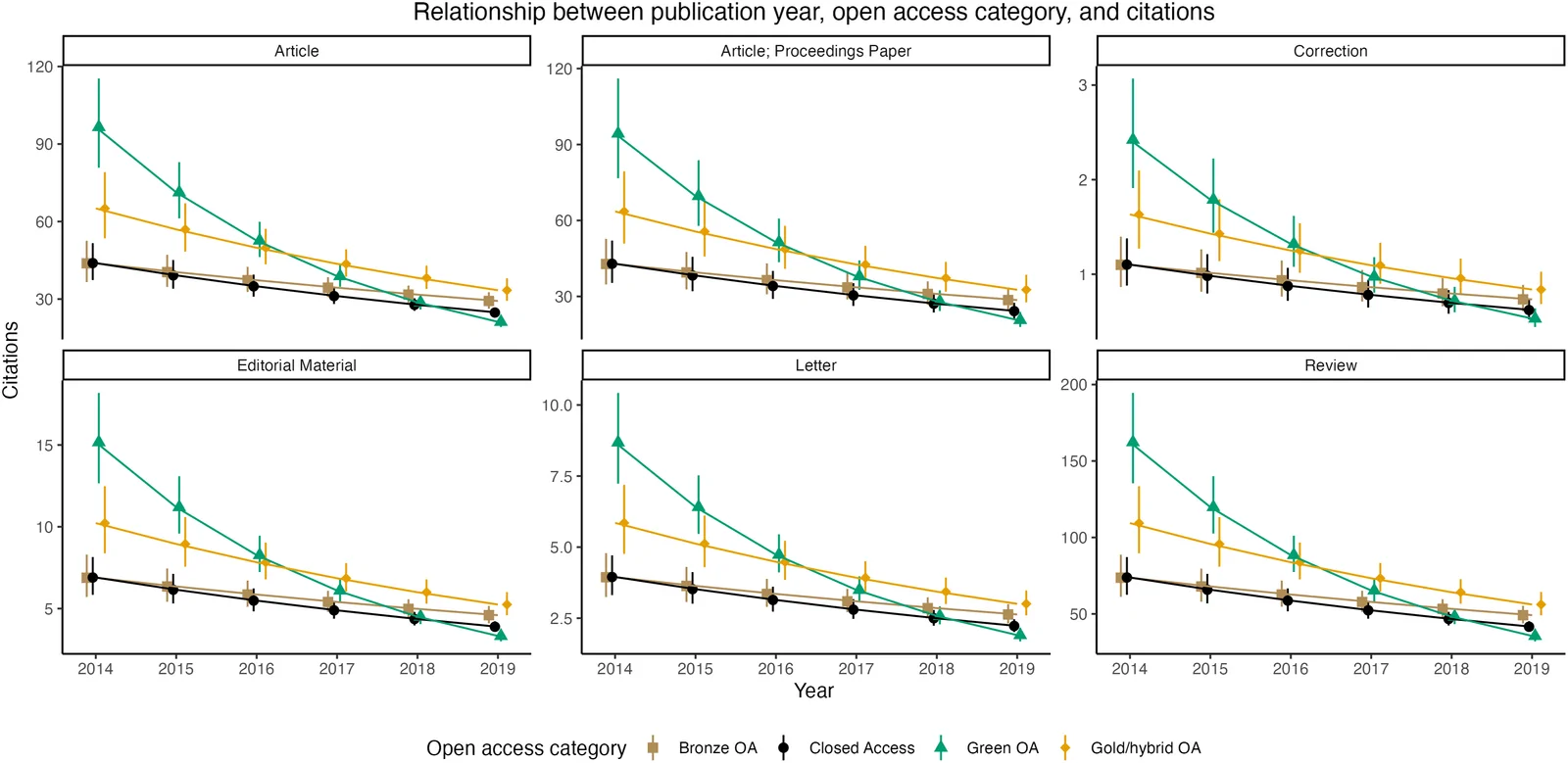
Citations as a function of publication year, stratified by OA category. Predicted mean citations by OA category across publication years. Points represent estimated means from the mixed-effects negative binomial model with error bars showing 95% confidence intervals; OA category is colour-coded as Gold/hybrid OA (gold), Green OA (green), Bronze OA (brown), and Closed Access (black). Citation counts decline across more recent cohorts for all OA categories, reflecting shorter citation accumulation windows. Gold/hybrid OA shows the highest predicted counts in the 2017–2019 cohorts, whereas Green OA leads in the 2014–2016 cohorts, with the relative pattern shifting around 2016–2017. Across document types, reviews accumulate the most citations, followed by original articles, while editorial materials, letters, and corrections receive markedly fewer citations across all OA categories.

### Citation rate by author count

Because the model includes no interaction between OA category and author count, the difference in citation rate between OA categories acts as a constant multiplier across all team sizes. Gold/hybrid OA articles had a citation rate 1.30 times that of Bronze OA (IRR = 1.30, p < 0.001) and 1.41 times as many citations as Closed Access. Green OA similarly maintained a citation rate 1.24 times that of Bronze OA (IRR = 1.24, p < 0.001), regardless of whether the article had a single author or a large collaboration team (Fig 3).

**Fig 3:**
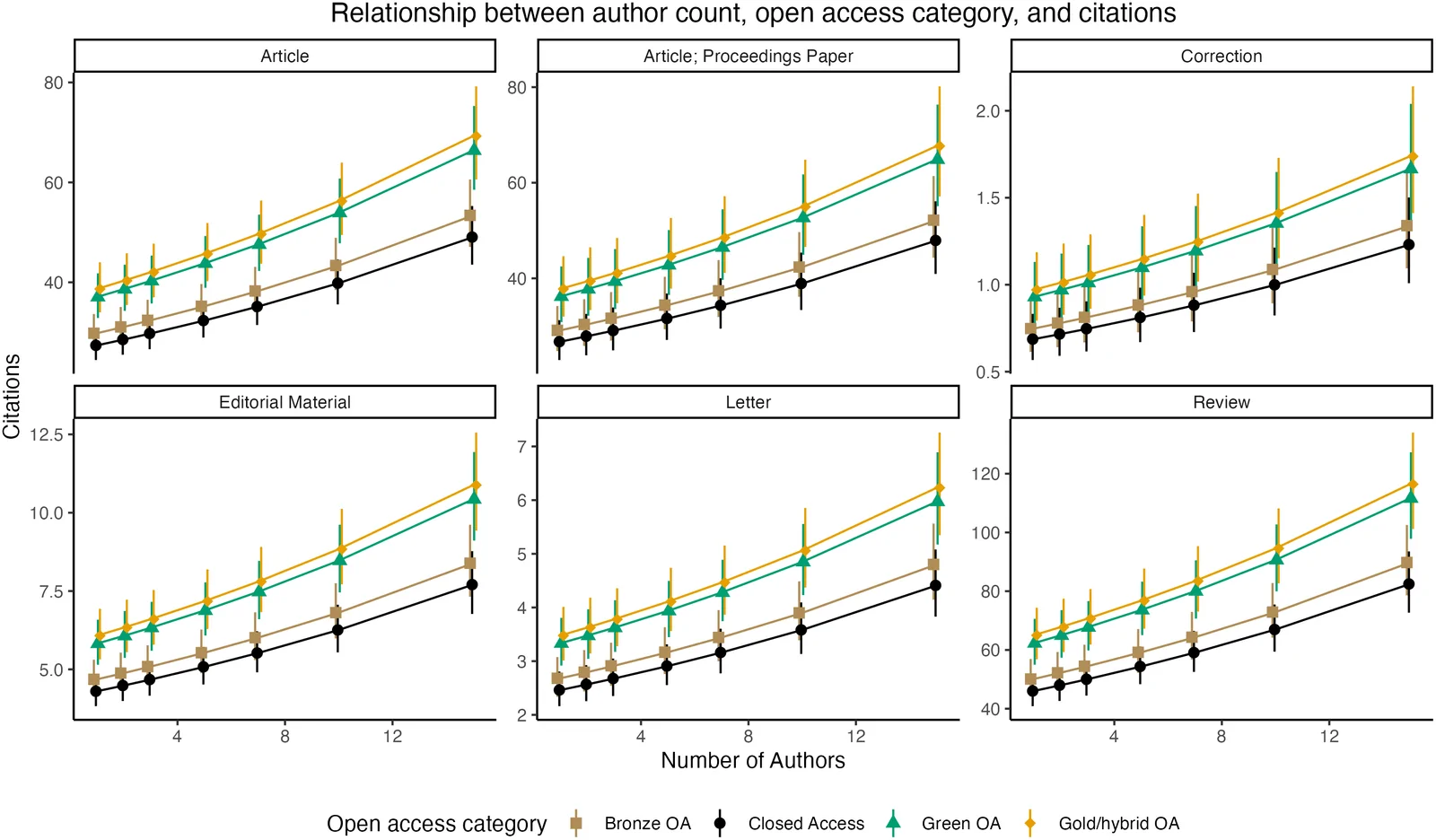
Citations as a function of author count, stratified by OA category. OA category is colour-coded: Gold/hybrid OA (gold), Green OA (green), Bronze OA (brown), and Closed Access (black). Points show predicted mean citations from the mixed-effects model at the mean publication year and AIS, with error bars indicating 95% confidence intervals; panels show each document type. Predicted citations increase with greater collaboration. Because the model contains no OA category × author count interaction, the ratio between OA categories is constant across author counts, so absolute differences widen as author count increases.

### Post hoc comparisons

Pairwise comparisons with Tukey adjustment showed significant differences for 5 of the 6 OA category pairs. Gold/hybrid OA had a citation rate 1.41 times that of Closed Access (95% CI 1.30–1.54, p < 0.001) and 1.30 times that of Bronze OA (95% CI 1.17–1.44, p < 0.001). The Gold/hybrid OA versus Green OA comparison was not significant (rate ratio 1.04, 95% CI 0.95–1.15, p = 0.687). Green OA had a citation rate 1.35 times that of Closed Access (95% CI 1.28–1.43, p < 0.001), and Bronze OA a citation rate 1.09 times that of Closed Access (95% CI 1.02–1.16, p = 0.003). Confidence intervals carry the same Tukey adjustment as the p-values reported with them.

### Layer 3: sensitivity analyses

Entropy balancing produced weights with a mean of 1.00 in every OA category. The maximum weight was 11.06 for Gold/hybrid OA, 4.02 for Bronze OA, 5.76 for Green OA and 3.69 for Closed Access. Weight variability was greatest for Gold/hybrid OA (coefficient of variation 1.17) and Bronze OA (1.12) and smallest for Closed Access (0.18). The most heavily weighted 1% of articles carried 3.7% of the total weight overall and 9.0% of the weight within the Gold/hybrid OA group, so no category was dominated by a small number of observations. The effective sample size was 386 of 911 (42.4%) for Gold/hybrid OA, 925 of 2,093 (44.2%) for Bronze OA, 1,431 of 2,187 (65.4%) for Green OA, and 18,343 of 18,924 (96.9%) for Closed Access. Generalized propensity scores estimated by multinomial logistic regression overlapped substantially across all four categories, but overlap was limited at the extremes of author count: 11 Green OA articles with 93 or more authors had an estimated Green OA propensity above 0.99, with no articles from other categories in that region, and none of the 45 articles with more than 32 authors was Gold/hybrid OA (S2 Table; S2 Fig).

Truncating the weights at the 99th percentile altered 239 weights (1.0% of the sample) and reduced the maximum weight from 11.06 to 2.99; truncation at the 97.5th and 95th percentiles altered 601 (2.5%) and 1,205 (5.0%) weights respectively. Covariate balance, which was exact before truncation, remained good after truncation at the 99th percentile (largest absolute standardized mean difference 0.055) and at the 97.5th percentile (0.086), but truncation at the 95th percentile reintroduced imbalance above the conventional 0.1 threshold for two covariates (largest absolute standardized mean difference 0.158), indicating that the most aggressive truncation reduces the influence of extreme weights at the cost of reintroducing confounding. Across all truncation schemes the citation rate ratios for Gold/hybrid OA versus Closed Access (1.41 untruncated; 1.44, 1.44 and 1.44 after truncation at the 99th, 97.5th and 95th percentiles) and for Green OA versus Closed Access (1.35 untruncated; 1.34, 1.31 and 1.27) were essentially unchanged, and Gold/hybrid OA and Green OA continued not to differ except under 95th-percentile truncation (rate ratio 1.13, p = 0.019). Two-sided truncation at the 1st and 99th percentiles gave results indistinguishable from one-sided truncation at the 99th percentile. The Bronze OA versus Closed Access comparison was the least stable: the rate ratio fell from 1.09 (p = 0.003) to 1.05 (p = 0.268) under 95th-percentile truncation (S2 Table).

When Green OA was separated into its three Web of Science subtypes, Green-submitted comprised 555 articles (2.3% of the sample), Green-accepted 823 (3.4%) and Green-published 809 (3.4%). All three subtypes had higher citation rates than Closed Access (rate ratios 1.58, 1.26 and 1.17 respectively; all p < 0.001). The subtypes differed from one another: Green-submitted had a higher citation rate than Green-accepted (1.25, 95% CI 1.07–1.46, p < 0.001) and Green-published (1.35, 95% CI 1.16–1.58, p < 0.001), while Green-accepted and Green-published did not differ (1.08, p = 0.545). Gold/hybrid OA had a higher citation rate than Green-published (1.22, 95% CI 1.07–1.39, p < 0.001) but did not differ from Green-submitted (p = 0.336) or Green-accepted (p = 0.114). Collapsing the three subtypes reproduced the primary estimates (S4 Table). These categories are not mutually exclusive in the underlying data: 798 of the 911 Gold/hybrid OA articles (87.6%) also carried a Green indicator and 362 of the 2,187 Green OA articles (16.6%) also carried a Bronze indicator, so each category describes the most privileged route available for an article rather than a single, exclusive publishing strategy. Because Web of Science records free-to-read status only at extraction, Bronze OA could not be subdivided by access duration.

Mean citations per year of follow-up were 2.31 for Bronze OA, 4.37 for Closed Access, 6.99 for Green OA and 9.53 for Gold/hybrid OA, preserving the ordering seen in cumulative counts (S5 Table). Refitting the primary model with an offset for the logarithm of years since publication, so that the outcome is the citation rate per year rather than the cumulative count, left the estimates essentially unchanged (Gold/hybrid OA versus Closed Access 1.41; Green OA versus Closed Access 1.36; Gold/hybrid OA versus Green OA 1.04, p = 0.687). Adding a journal-by-publication-year random intercept, which absorbs any journal-level characteristic that varies by year including year-specific journal influence, improved model fit (AIC 202,563 versus 202,772) and yielded a journal-by-year standard deviation of 0.118 across 60 journal-year cells, smaller than the between-journal standard deviation of 0.160; the OA estimates were again essentially unchanged (Gold/hybrid OA versus Closed Access 1.40; Green OA versus Closed Access 1.31; Gold/hybrid OA versus Green OA 1.07, p = 0.278) (S5 Table).

Removing the entropy-balancing weights altered one comparison materially: without weighting, Bronze OA had a lower citation rate than Closed Access (0.93, 95% CI 0.86–0.99, p = 0.026), the reverse of the weighted estimate, while the Gold/hybrid OA and Green OA estimates retained their direction and approximate magnitude (1.37 and 1.27 times Closed Access respectively). All specifications are compared in S5 Table and S3 Fig. Taken together, the higher citation rates of Gold/hybrid OA and Green OA relative to Closed Access were stable across every specification examined, whereas the small difference between Bronze OA and Closed Access was sensitive to both the weighting and the degree of weight truncation and should be interpreted with corresponding caution.

## Discussion

The present study evaluated the citation impact of various OA models within orthopaedic surgery journals. Our findings are consistent with a higher citation rate associated with OA publishing, particularly for Gold/hybrid OA articles, which had a 41% higher citation rate than traditional Closed Access articles. The difference between Bronze OA and Closed Access was small and its direction depended on the model specification, and we therefore do not draw a directional conclusion for that comparison; the higher citation rates of Gold/hybrid OA and Green OA relative to Closed Access were by contrast stable across all specifications. Green OA articles also had higher citation rate than Closed Access (rate ratio 1.35), with an effect estimate similar to Gold/hybrid versus Closed Access (rate ratio 1.41), although Gold/hybrid and Green OA did not differ significantly from one another. Importantly, these associations persisted after adjustment for document type, publication year, collaboration, and journal influence, indicating that both the OA model and article type contribute independently to citation patterns. These findings align with previous research highlighting citation differences associated with OA publishing across multiple disciplines, including the biomedical sciences [5,15,16].

Gold/hybrid OA had the highest citation rate in more recent publication cohorts, consistent with the potential effect of immediate unrestricted availability at the time of publication. This pattern may reflect early visibility, broader readership, and faster citation uptake shortly after publication [17]. By removing access barriers at publication, Gold/hybrid OA may be associated with increased readership when interest in a new article is highest, which may explain its higher citation rate in cohorts with shorter follow-up [18]. However, this pattern did not persist uniformly across older cohorts, in which Green OA showed the higher predicted citation rate.

In contrast to Gold/hybrid OA, Green OA appeared to show a delayed pattern. Because Green OA often involves an embargo period of six or more months before public availability, any difference in citation rate may be less apparent shortly after publication [19]. In our temporal analysis, Green OA had lower predicted citation rates than Gold/hybrid OA in more recent cohorts, but higher predicted rates in older cohorts with longer follow-up. This pattern is consistent with a delayed “catch-up” effect, whereby repository-based access may become increasingly relevant after embargo periods expire and archived versions have had more time to circulate. Our findings therefore suggest that Gold/hybrid OA is associated with a higher citation rate early after publication, whereas Green OA is associated with a higher citation rate over longer follow-up. Separating the Green subtypes refines this picture: Green-submitted, which corresponds to repository deposit of a pre-acceptance version, had the highest citation rate of the three subtypes, whereas Green-published had the lowest. Because subtypes are assigned by the most-final version, this contrast reflects deposit patterns rather than access dates alone. Green-submitted versions are not usually subject to publisher embargoes, yet this subtype also showed the steepest decline in citation rate across publication cohorts (S4 Table), so the higher Green OA citation rate in older cohorts is unlikely to be explained by embargo timing alone..

Bronze OA showed a more nuanced pattern. Although Bronze OA had the lowest citation counts in unadjusted analyses, this appeared largely attributable to its enrichment for lower-cited publication types, including editorials, letters, and corrections. After adjustment, the difference between Bronze OA and Closed Access was small and its direction depended on the specification: Bronze OA had a modestly higher citation rate than Closed Access in the primary weighted model but a modestly lower rate in the unweighted model, and the difference was not significant under the most aggressive weight truncation. We therefore do not draw a directional conclusion about Bronze OA relative to Closed Access. Because Bronze OA articles are free to read but often lack a clear open license and may be made available temporarily or at the publisher’s discretion, and because Web of Science records free-to-read status only at the time of extraction, this category should not be interpreted as equivalent to licensed OA models such as Gold/hybrid OA or repository-based Green OA. This unpredictability may limit long-term access and discoverability and may help explain why Bronze OA did not reach the citation rates of Gold/hybrid OA or Green OA [5].

Beyond access routes, the citation rate increased with the number of authors (Fig 3), consistent with prior work suggesting that collaborative research is associated with a higher citation rate. This aligns with previous findings that suggest larger teams often achieve broader visibility and scholarly recognition [7]. Author count was modelled as a single log-linear term with no interaction with OA category, so collaboration and access route are represented as having additive (on the log scale), rather than competing, associations with citation rate [6,20]; within this specification, Gold/hybrid OA had the highest predicted citations across the full range of author counts. Whether the association between collaboration and citation rate differs by OA route was not tested. These findings suggest that OA route is only one component of citation rate, alongside collaboration, journal influence, publication year, and article type.

Our findings contribute to a growing yet mixed body of literature on OA citation rates in orthopaedics. While some studies, such as that by Zhang et al. in 2021, suggest a higher citation rate for Closed Access publications, others have reported no significant differences between OA and non-OA models [12,13,21]. Cooke and Jain (2021) found similar citation rates between OA and subscription-based journals despite differing impact factors [22]. However, emerging data, such as that from Sidharthan et al. (2024), suggest that OA may be associated with a modest long-term increase in citation rate [23]. While their findings were specific to pediatric orthopaedics, our study extends this evidence to a broader sample of high-impact orthopaedic surgery journals, while noting that our comparison is between OA routes within such journals rather than between OA and subscription journals.

Despite the higher citation rates observed for Gold/hybrid OA articles, these findings should be weighed against the economic considerations related to high APCs, which were not measured in this study. These costs may exclude researchers from lower-resource institutions and regions, potentially reinforcing disparities between institutions and researchers [8,9]. Prior work has also suggested that high APCs do not necessarily correlate with a higher citation rate, suggesting that journals’ prestige and marketing may disproportionately influence citation rates rather than the intrinsic quality of research alone [7]. Similarly, Pfeil et al. (2025) found that hybrid orthopaedic journals charged significantly higher APCs than fully OA journals, despite no meaningful difference in citation rates between the two models [24]. These findings challenge the assumption that higher publishing costs yield a higher citation rate and underscore the importance of cost-effectiveness when choosing an OA route.

Several limitations of our study should be acknowledged. First, because our sample was restricted to top-quartile journals indexed in Web of Science that publish predominantly on a subscription or hybrid basis, our findings describe the choice between OA routes within such journals. They do not compare fully OA journals with subscription journals, and they may not generalize to mid-tier or subspecialty orthopaedic journals, where OA uptake, APC levels, and licensing practices may differ. Second, OA categories were defined using Web of Science indicators, which are database-derived access labels rather than homogeneous publishing strategies. A single article may carry several indicators, and our precedence rule assigns each article to the most privileged route available to it: 798 of the 911 Gold/hybrid OA articles (87.6%) also carried a Green indicator, and 362 of the 2,187 Green OA articles (16.6%) also carried a Bronze indicator. The Web of Science OA field also records free-to-read status only at the time of extraction, so the duration and stability of Bronze access could not be determined. The absence of article-level licensing, embargo, and APC data may therefore have introduced residual misclassification or confounding between access groups.

Third, we intentionally excluded journals in which >95% of articles were published as OA. This design allowed OA routes to be compared within hybrid or subscription-based journals, but it means that fully OA journals are not represented and that the Gold/hybrid OA category consists entirely of hybrid OA articles; our findings should not be read as a comparison of fully OA journals with subscription journals. Fourth, citation counts are cumulative totals at the date of extraction rather than counts accrued within a fixed window, so follow-up ranged from approximately five years for the 2019 cohort to approximately ten years for the 2014 cohort. Publication year is modelled explicitly and a citations-per-year specification gave essentially identical estimates, but a standardized citation window could not be constructed from the available extract. Fifth, journal influence was represented by a single Article Influence Score per journal rather than a year-specific value; adding a journal-by-publication-year random intercept, which absorbs any journal-level characteristic that varies by year, left the estimates essentially unchanged. Sixth, our analysis was restricted to orthopaedic surgery journals; thus, OA policies, adoption, and citation patterns observed may not generalize to other surgical or biomedical specialties. In addition, we were unable to systematically account for journal-, funder-, or institution-level OA mandates and policies, which may influence both OA adoption and citation rate and therefore confound the associations observed. Seventh, the exclusion of direct APC data restricts our ability to provide concrete economic insights into the cost-effectiveness of various OA routes. Finally, because this is an observational bibliometric analysis, all findings represent associations rather than causal effects.

Future research should incorporate APC data, licensing, and embargo data to enable a more comprehensive cost-benefit analysis of OA publishing in orthopaedics. Linking articles to repository deposit dates and to the duration of free-to-read availability would allow the Green subtypes and Bronze access to be characterised as publishing strategies rather than as database-derived labels. Expanding analyses to include a broader range of journal tiers, subspecialties, fully OA journals, and recent publication data post-2019 would further clarify evolving OA trends and their sustainability. Finally, studies comparing sustained OA availability with temporary free-to-read access may provide valuable insight into the long-term citation patterns associated with different OA routes.

## Conclusion

Within ten high-impact orthopaedic journals that publish predominantly on a subscription or hybrid basis, citation rates differed by the OA route through which an article became freely readable. In the primary adjusted analysis, Gold/hybrid OA articles had a citation rate 1.41 times that of Closed Access articles and 1.30 times that of Bronze OA articles, and Green OA articles had a citation rate 1.35 times that of Closed Access articles; Gold/hybrid OA and Green OA did not differ from one another. These differences were stable across all sensitivity analyses. Bronze OA had the lowest citation counts in unadjusted analyses; however, this appeared largely attributable to document-type composition, as Bronze OA was enriched for editorials, letters, and corrections. After adjustment the difference between Bronze OA and Closed Access was small and depended on the specification, and we therefore do not draw a directional conclusion for that comparison.

Citation rates also differed by publication year, article type, and author collaboration. Gold/hybrid OA had the highest predicted citation rate in more recent publication cohorts with shorter follow-up, whereas Green OA had the highest predicted rate in older cohorts with longer follow-up. Reviews and multi-author articles had higher citation rates. These findings describe the choice between OA routes within subscription-based and hybrid orthopaedic journals; because journals publishing more than 95% of their content as OA were excluded by design, they do not speak to the comparison between fully OA and subscription journals. For orthopaedic researchers and practicing orthopaedic surgeons, they may nonetheless help inform decisions about publication and dissemination strategies within the journals in which most orthopaedic research currently appears.

## Supporting information

S1 Fig Journal and article selection flow diagram. (TIFF)

S2 Fig Entropy-balancing weight distribution and common support. (TIFF)

S3 Fig Pairwise citation rate ratios across all model specifications. (TIFF)

S1 Table Journal screening log: outcome and reason for every journal assessed. (CSV)

S2 Table Weighting diagnostics. (A) Entropy-balancing weight distribution and effective sample size by open access category. (B) Common support: generalised propensity score distribution. (C) Weight truncation: weight diagnostics and covariate balance.
(CSV)

S3 Table Standardized residuals from the chi-squared test of open access category by document type. (CSV)

S4 Table Green open access subtypes. (A) Descriptive statistics. (B) Effective sample size after weighting. (C) Incidence rate ratios. (D) Pairwise comparisons. (E) Overlap between Web of Science open access designations.
(CSV)

S5 Table Model specifications. (A) Pairwise citation rate ratios across all specifications. (B) Model fit. (C) Citations per year of follow-up. (D) Follow-up window by publication cohort. (E) Journal-by-publication-year variance components.
(CSV)

S1 File Processed citation data. (ZIP)
