# Supplementary material for: Open access route and citation impact in orthopaedic research: A comparison of Gold/hybrid, Green, and Bronze open access within ten high-impact subscription-based journals
Source: PLoS One. 2026 Nov 4;21(11):e0360532. doi: 10.1371/journal.pone.0360532 (PMC13734443; doi:10.1371/journal.pone.0360532)
Supplement: Supplementary file 4 [file pone.0360532.s013.docx]

May 22^nd^, 2026

**Manuscript ID:** PONE-D-25-46173

**Manuscript Title:** Is OA the way? A comparison of different open access classifications and their benefits in orthopaedic research

Dear Editor and Reviewers at *PLOS ONE*,

We sincerely thank the reviewers for their thoughtful and constructive feedback on our manuscript, “Is OA the way? A comparison of different open access classifications and their benefits in orthopaedic research”. We appreciate the opportunity to revise our work. In response to the reviewers’ comments, we have clarified the source and handling of Article Influence Score, improved consistency across the Abstract, Results, Discussion, figure captions, and Conclusion, expanded the interpretation of Bronze OA, strengthened the statistical methods and reporting, and added additional limitations regarding APC data and the observational nature of the study. We have also performed a line-by-line review to improve clarity, grammar, formatting, and consistency throughout the manuscript.

All changes have been made in the revised manuscript using tracked changes.

Sincerely,

Michelle Cruickshank, MD (candidate)

Corresponding author: [cruicm5@mcmaster.ca](mailto:cruicm5@mcmaster.ca)

On behalf of co-authors:

William Daniels, MD (candidate), Taylor Dejong, MD (candidate), Michelle Ghert, MD, FRCSC

**Reviewer #1:**

**Comment:**

**My main concern is that the description of AIS is still contradictory across the manuscript and the rebuttal. In the Methods, AIS is first described in connection with JCR, whereas the article-level metadata section states that AIS values were obtained from the 2024 SJR. In the response to reviewers, the authors again describe AIS as coming from SCImago and state that no imputation was performed. These descriptions need to be reconciled so that the manuscript provides a single, definitive, and internally consistent account of the AIS source, timing, and handling.**

Response:

Thank you for identifying this important inconsistency. We agree that the prior wording created confusion regarding the source and handling of AIS. We have revised the manuscript to provide a single, consistent description throughout. AIS is now consistently described as a journal-level metric obtained from the 2024 SCImago journal reports and matched to articles by journal title. We also clarified that AIS values were available for all included journals and that no imputation or substitution was performed.

Revision made:

We revised the Journal Identification and Individual Article Metadata Extraction sections to consistently state the source, timing, and handling of AIS. We also reviewed the manuscript to ensure that AIS is no longer described inconsistently across sections.

**Comment:**

**A second concern is that the interpretation of the open-access categories remains only partially aligned across sections. The adjusted model results indicate that Bronze OA modestly outperformed Closed Access. In contrast, the abstract conclusion still states that Gold was followed by Green and Closed Access and that Bronze underperformed. Those statements are not fully compatible with the adjusted comparisons presented in the main Results. The manuscript would be strengthened by ensuring that the abstract, Results, Discussion, figure captions, and conclusion all reflect the same hierarchy of findings and do not mix adjusted and unadjusted interpretations.**

Response:

Thank you for highlighting this important inconsistency. We revised the Abstract, Results, Discussion, figure captions, and Conclusion to distinguish more clearly between unadjusted and adjusted findings. In unadjusted analyses, Bronze OA had the lowest citation counts; however, additional document-type analyses showed that Bronze OA was enriched for lower-cited publication types, including editorial materials, letters, and corrections. After adjustment for document type and other covariates, the apparent Bronze OA citation deficit was substantially attenuated, and Bronze OA performed similarly to or modestly better than Closed Access while remaining below Gold and Green OA. We also clarified that Gold and Green OA did not differ significantly overall, although their relative performance varied by publication cohort. Specifically, Gold OA showed higher predicted citation counts in more recent cohorts, whereas Green OA surpassed Gold OA in older cohorts with longer citation windows.Revision made:

We revised the Abstract, Results, Discussion, Figure 1 caption, and Conclusion to distinguish clearly between unadjusted and adjusted findings. We also expanded Figure 1 and the accompanying Results text to describe document-type composition across OA categories and the articles-only analysis.

**Comment:**

**The presentation of the temporal interaction also still needs tightening. The main Results section appropriately describes a crossover between Green and Gold OA around 2016 to 2017, with Green performing better in older cohorts and Gold in more recent cohorts. However, the wording around this pattern is not yet fully polished, and the relevant figure caption remains awkward and internally unclear. This should be revised so that the temporal interpretation is stated once, clearly and consistently, without ambiguity.**

Response:

Thank you for this helpful suggestion. We agree that the temporal interaction required clearer and more consistent wording. We revised the Results, Discussion, Figure 2 caption, and Conclusion to describe the crossover between Green and Gold OA more clearly. The revised manuscript now states that Green OA had higher predicted citation counts in older publication cohorts, whereas Gold OA had higher predicted citation counts in more recent cohorts, with the relative advantage shifting around 2016–2017.

We also clarified that this analysis compares cumulative citation counts across publication-year cohorts rather than following individual articles longitudinally. Therefore, the temporal interaction should be interpreted as an association across cohorts rather than direct evidence of within-article citation accumulation over time.

Revision made:

We revised the temporal interaction section of the Results, the Figure 2 caption, the Discussion, and the Conclusion to ensure that the Green-versus-Gold crossover is described consistently and without ambiguity.

**Comment:**

**In addition, the manuscript still requires a careful language and formatting review before publication. There are multiple visible residual problems, including typographical errors, grammar issues, awkward or incomplete sentences, formatting artifacts, and malformed reporting in the post hoc comparison section. Because PLOS ONE does not copyedit accepted manuscripts, these issues should be corrected carefully at revision. I strongly encourage the authors to perform one final line-by-line review of the abstract, Methods, Results, figure captions, and discussion to ensure that the manuscript is not only statistically coherent but also clear and publication-ready in its presentation.**

Response:

Thank you for this important comment. We agree that the manuscript required additional language and formatting refinement prior to publication. We performed a detailed line-by-line review of the Abstract, Methods, Results, figure captions, Discussion, and Conclusion. We reviewed typographical errors, grammar issues, formatting artifacts, and incomplete or awkward sentences. We also revised the post hoc comparison section to improve clarity and ensure that effect estimates, confidence intervals, and p-values are reported consistently.

Revision made:

We performed a manuscript-wide language and formatting review and revised the Abstract, Methods, Results, figure captions, Discussion, Conclusion, and post hoc comparison section for clarity, consistency, and publication readiness.

**Reviewer #2**

**Comment:**

**• Bronze OA requires more clarity.**

Response:

Thank you for this helpful comment. We agree that the Bronze OA category required additional clarification. We revised the Methods to define Bronze OA more clearly as free-to-read articles available on the publisher’s website without a clear open license. We also expanded the Results and Discussion to explain that Bronze OA is heterogeneous and may include articles made freely available temporarily or at the publisher’s discretion.

In addition, we added new analyses showing that Bronze OA was disproportionately composed of lower-cited publication types, including editorial materials, letters, and corrections. This helped explain why Bronze OA had the lowest citation counts in unadjusted analyses despite modestly outperforming Closed Access in the adjusted model.

Revision made:

We clarified the definition of Bronze OA in the Methods and Table 1, expanded the Results to describe Bronze OA document-type composition, and revised the Discussion and Conclusion to distinguish the unadjusted and adjusted Bronze OA findings.

**• There is no APC data to help in cost benefit analysis, please include this in recommendations for future research.**

Response:

Thank you for this point. We agree that the lack of article-level APC data limits our ability to assess cost-effectiveness across OA models. We have added this point to both the Limitations and Future Directions sections. We state that future research should incorporate article-level APC, licensing, and embargo data to enable a more comprehensive cost-benefit analysis of OA publishing in orthopaedics.

Revision made:

We added the absence of article-level APC data as a limitation and revised the Future Directions paragraph to recommend incorporation of APC data in future studies.

**• The study has not established causality-this should be included in the limitations.**

Response:

Thank you for this important comment. We agree. Because this study is observational and bibliometric in design, it cannot establish causal relationships between OA status and citation counts. We have added this explicitly to the Limitations section and revised language throughout the manuscript to avoid causal interpretation.

Revision made:

We added a final sentence to the Limitations section stating that, because this was an observational bibliometric analysis, all findings should be interpreted as associations rather than causal effects. We also revised causal wording throughout the manuscript where appropriate.

**• The work has not clearly stated the distribution-Poisson/Negative Binomial .It requires more explanation.**

Response:

Thank you for this helpful comment. We agree that the model distribution and rationale required clearer explanation. We expanded the Statistical Analysis section to explain that citation counts are overdispersed count data. Although a Poisson model was initially considered, likelihood ratio testing showed substantial overdispersion and demonstrated that the negative binomial model provided a substantially better fit. We also clarified that a zero-inflated negative binomial model was assessed but did not improve model fit, indicating that the standard negative binomial model adequately captured the observed zero-citation articles.

Revision made:

We expanded the Statistical Analysis section to include model justification, comparison of Poisson and negative binomial models, assessment of zero inflation, and rationale for the final negative binomial GLMM.

**• IRRs require more explanation.**

Response:

Thank you for this suggestion. We revised the Statistical Analysis and Results sections to clarify that model coefficients were exponentiated and reported as incidence rate ratios. We also added explanatory language indicating that IRRs represent multiplicative differences in expected citation rates between groups, after adjustment for model covariates.

Revision made:

We clarified the interpretation of IRRs in the Statistical Analysis section and revised the Results to report IRRs in a more reader-friendly manner.

**• Correlations between predictors have not been made clear.**

Response:

We thank you for raising this point. We clarified the model structure and interpretation of random effects, including the correlation between journal-specific random intercepts and publication-year slopes. We now report that the strong negative correlation between intercepts and slopes indicates that journals with higher baseline citation rates showed attenuated citation growth over time. We also clarified that continuous predictors were standardized prior to analysis to improve model convergence and facilitate interpretation.

Revision made:

We revised the Model Structure and Advanced Model Results sections to clarify the correlation between random intercepts and slopes and to improve explanation of standardized predictors.

**• More explanation of statistical results is required.**

Response:

Thank you for this comment. We expanded the Statistical Analysis and Results sections to improve the explanation of the model and its findings. Specifically, we added clearer descriptions of entropy balancing, model fit, overdispersion testing, zero-inflation assessment, Type II Wald chi-square tests, post hoc pairwise comparisons, and interpretation of the OA type x publication year interaction.

Revision made:

We revised the Statistical Analysis and Results sections to provide additional explanation of model selection, model diagnostics, covariate balance, IRRs, post hoc comparisons, and temporal interaction findings.

**• Figures require more explanatory captions.**

Response:

We thank you for this suggestion. We revised the figure captions to make them more self-contained and explanatory. Figure 1 now describes the unadjusted citation distributions, document-type composition across OA categories, standardized residuals from the chi-square analysis, and the articles-only analysis. Figure 2 now more clearly describes citation counts by publication year and OA category, including the shift in relative performance between Gold and Green OA across publication cohorts. Figure 3 now describes the association between author count and citation counts across OA categories.

Revision made:

We expanded and clarified the captions for Figures 1-3 to improve interpretability and to better align each figure with the corresponding Results text.
