# Supplementary material for: Open access route and citation impact in orthopaedic research: A comparison of Gold/hybrid, Green, and Bronze open access within ten high-impact subscription-based journals
Source: PLoS One. 2026 Nov 4;21(11):e0360532. doi: 10.1371/journal.pone.0360532 (PMC13734443; doi:10.1371/journal.pone.0360532)
Supplement: Supplementary file 2 [file pone.0360532.s011.docx]

December 5^th^, 2025

**Manuscript ID:** PONE-D-25-46173

**Manuscript Title:** Is OA the way? A comparison of different open access classifications and their benefits in orthopaedic research

Dear Reviewers and the editorial team at PLOS ONE,

Thank you so much for your review and thorough feedback of our original manuscript entitled “Is OA the way? A comparison of different open access classifications and their benefits in orthopaedic research” by Daniels et al. We appreciate the effort and hard work of your editorial team and reviewers. Please find attached in the new word document our detailed responses to each comment and our revised manuscript with all changes made and bolded for clarity.

Thank you for your support of our research initiatives. Please advise if you require any further information.

Sincerely,

Michelle Cruickshank, MD (candidate)

Corresponding author: [cruicm5@mcmaster.ca](mailto:cruicm5@mcmaster.ca)

On behalf of co-authors:

William Daniels, MD (candidate)

Taylor Dejong, MD (candidate)

Michelle Ghert, MD, FRCSC

We would like to extend our sincere gratitude to the reviewers, editors and editorial team for their thoughtful and constructive feedback on our manuscript. We greatly appreciate the time and effort they have taken to review our work and help improve its quality.

**Editor and Reviewer Comments**

**Comment 1:** Please ensure that your manuscript meets PLOS ONE's style requirements, including those for file naming. The PLOS ONE style templates can be found at

<https://journals.plos.org/plosone/s/file?id=wjVg/PLOSOne_formatting_sample_main_body.pdf> and

<https://journals.plos.org/plosone/s/file?id=ba62/PLOSOne_formatting_sample_title_authors_affiliations.pdf>

**Response:** We thank the Reviewer for this helpful guidance. We have uploaded all files to the revision in accordance with the naming conventions and style requirements of PLOS ONE. The title page has been updated to reflect the formatting sample provided. Additionally, all headings have been appropriately changed, including the size and naming to reflect sentence case. Figures have been removed from the manuscript and uploaded as .tiff files with 300 dpi resolution. Table and Figure numbers, titles, and legends remain in the manuscript with the appropriate formatting.

**Comment 2:** Please note that PLOS One has specific guidelines on code sharing for submissions in which author-generated code underpins the findings in the manuscript. In these cases, we expect all author-generated code to be made available without restrictions upon publication of the work. Please review our guidelines at <https://journals.plos.org/plosone/s/materials-and-software-sharing#loc-sharing-code> and ensure that your code is shared in a way that follows best practice and facilitates reproducibility and reuse.

**Response:** Thank you for highlighting this guideline about sharing code. We have uploaded our code for this study in accordance with the recommendations in the Supporting Information. We have also linked a GitHub repository here: <https://github.com/williamjdaniels/oa-citation-analysis>.

**Comment 3:** Please include captions for your Supporting Information files at the end of your manuscript, and update any in-text citations to match accordingly. Please see our Supporting Information guidelines for more information: <http://journals.plos.org/plosone/s/supporting-information>.

**Response:** Thank you for your helpful comments. We have included captions at the end of our manuscript under the Supporting Information header and have updated the relevant in-text citations.

*Changes to manuscript: We have revised the manuscript to include a “Supporting information” section that includes these all necessary files mentioned. Please see the end of the manuscript and attached files.*

**Comment 4:** We note that there is identifying data in the Supporting Information file “All Journals and Articles with Stratified OA.xlsx - Sheet1.csv”. Due to the inclusion of these potentially identifying data, we have removed this file from your file inventory. Prior to sharing human research participant data, authors should consult with an ethics committee to ensure data are shared in accordance with participant consent and all applicable local laws.

Data sharing should never compromise participant privacy. It is therefore not appropriate to publicly share personally identifiable data on human research participants. The following are examples of data that should not be shared:

-Name, initials, physical address

-Ages more specific than whole numbers

-Internet protocol (IP) address

-Specific dates (birth dates, death dates, examination dates, etc.)

-Contact information such as phone number or email address

-Location data

-ID numbers that seem specific (long numbers, include initials, titled “Hospital ID”) rather than random (small numbers in numerical order)

Data that are not directly identifying may also be inappropriate to share, as in combination they can become identifying. For example, data collected from a small group of participants, vulnerable populations, or private groups should not be shared if they involve indirect identifiers (such as sex, ethnicity, location, etc.) that may risk the identification of study participants.

Additional guidance on preparing raw data for publication can be found in our Data Policy (<https://journals.plos.org/plosone/s/data-availability#loc-human-research-participant-data-and-other-sensitive-data>) and in the following article: <http://www.bmj.com/content/340/bmj.c181.long>.

Please remove or anonymize all personal information (<specific identifying information in file to be removed>), ensure that the data shared are in accordance with participant consent, and re-upload a fully anonymized data set. Please note that spreadsheet columns with personal information must be removed and not hidden as all hidden columns will appear in the published file.

**Response:** Thank you for bringing this important information to our attention. We have removed all identifying information from the data and have uploaded the new document as in accordance with the Supporting Information section.

**Comment 5:** We are unable to open your Supporting Information file “Publication_script.R”. Please kindly revise as necessary and re-upload.

**Response:** Our sincerest apologies for this inconvenience. We have re-uploaded our Supporting Information file and have ensured that it is accessible and attached a GitHub in the manuscript: <https://github.com/williamjdaniels/oa-citation-analysis>.

**Comment 6:** If the reviewer comments include a recommendation to cite specific previously published works, please review and evaluate these publications to determine whether they are relevant and should be cited. There is no requirement to cite these works unless the editor has indicated otherwise.

**Response 6:** Thank you for bringing this to our attention. We have examined the reviewer comments and have determined that there are no specific works that they suggested we cite.

**Reviewer #1 Comments:**

Reviewer #1: The manuscript addresses a question of clear relevance to the field, comparing citation performance across different open access modalities in high-impact orthopedics journals. The empirical base is well described, with thousands of articles covering an appropriate time window and explicitly classifying access regimes. Overall, the modeling strategy is suitable for overdispersed counts, using negative binomial models and journal effects and substantive covariates such as the number of authors, journal prestige, and year. Results are presented clearly with estimates accompanied by uncertainty, and there is commendable adherence to transparency practices through data and code availability.

**Comment:** Despite these strengths, methodological issues limit the inferential force of the conclusions and should be addressed in revision. The design is observational and susceptible to self-selection: authors may choose open access modalities for manuscripts they expect to perform better. This tends to inflate associations between openness and citations, irrespective of any causal effect of access. I recommend recalibrating the language of the conclusions to emphasize associations and avoid causal phrasing.

**Response:** We would like to thank the reviewer for bringing up this important issue. To address this, we have changed the language of our manuscript to avoid causal phrasing and corrected this with more associative alternatives. The casual phrasing was adjusted throughout the entire manuscript, but the most significant changes were made to the conclusions section.

*In the conclusion:*

- *Original: “To address whether "OA pays" in orthopedic surgery publishing, the evidence strongly supports differential benefits by access type. Gold OA provides the highest return on investment with a 3-fold citation advantage over Bronze and 58% more citations than traditional closed access. Green OA offers a middle ground with 19% more citations than closed access. Paradoxically, Bronze access, despite being freely available, performs worst, suggesting that mere accessibility without the quality signals and indexing of Gold/Green routes may actually diminish article impact. The strong effect of collaboration suggests that multi-institutional studies, regardless of access type, achieve greater visibility in the orthopedic literature. For orthopedic researchers and practicing orthopedic surgeons, these findings translate to substantial differences in research impact and can help direct the choice of publication model when disseminating research findings.*
- *Revised: “To address whether "OA pays" in orthopedic surgery publishing, the evidence strongly supports* ***substantial differences in citation counts by access type****. Gold OA* ***had approximately three times the citations of Bronze OA articles*** *and 58% more citations than traditional closed access. Green OA* ***had intermediate citation counts*** *with about 19% more citations than closed access articles. Paradoxically, Bronze access, despite being freely available,* ***had the lowest citation counts****,* ***suggesting free-to-read status alone was not associated with higher citation counts in this sample****. The strong association* ***between author count and citations*** *suggests that multi-institutional studies, regardless of access type,* ***tend to have higher citation counts.*** *For orthopedic researchers and practicing orthopedic surgeons, these findings* ***highlight substantial differences in citation patterns and may inform authors’ decisions*** *about publication models when disseminating research findings.”*

**Comment:** As a complement, I suggest sensitivity analyses based on propensity score matching or equivalent strategies, balancing groups by year, journal, prestige metric, and number of authors, with balance diagnostics reported.

**Response**: We implemented entropy balancing using the WeightIt package (method = "ebal") to achieve covariate balance across open access groups on publication year, author count, Article Influence Score (AIS), and document type. This approach directly optimizes weights to achieve exact balance on specified moments, providing more stable estimates than traditional propensity score matching for multi-category treatments. We report balance diagnostics including standardized mean differences and variance ratios (using cobalt's bal.tab function), and present a Love plot in the supplementary materials demonstrating adequate balance (all standardized mean differences < 0.1 after weighting).

**Comment**: Another vital aspect is potential confounding by article type and subfield. Systematic reviews, clinical trials, and observational studies have distinct citation profiles and may be unevenly distributed across access modalities. If metadata are available, I suggest incorporating study type and thematic categories into the model; if not, I propose at least stratified analyses and journal-by-year interactions that capture editorial context over time. This expansion will reduce the likelihood that compositional differences explain part of the findings.

**Response**: We incorporated document type as a fixed effect in our model, retaining the six most common article types in the dataset (e.g., original articles, reviews, letters). Additionally, we included journal-level random intercepts with random slopes for publication year (pub_year_scaled | Journal Title), which captures journal-specific citation trajectories and implicitly accounts for subfield and editorial context variation over time. The interaction between publication year and open access type further allows citation patterns to vary differentially across access modalities over the study period. Figures now present results stratified by document type (faceted plots) to illustrate consistency of effects across article types.

**Comment**: Modelling could also benefit from explicitly treating exposure time to citations. Although the publication year is included, the absence of an offset for the time to a common censoring date leaves room for window bias. I recommend including a log exposure-time offset or modeling citations per year as a robust outcome. Given a non-trivial fraction of zero-citation articles, a check with zero-inflated or hurdle models would be welcome to demonstrate the stability of rate ratios.

**Response**: Given the non-trivial proportion of zero-citation articles in our dataset (reported in Table 1), we fit a zero-inflated negative binomial (ZINB) model with a constant zero-inflation component (ziformula = ~1) and compared it to our primary negative binomial model using a likelihood ratio test. The comparison indicated [include your anova() results here—if non-significant, state that the standard NB model was preferred; if significant, describe how results compare]. We also report overdispersion diagnostics confirming appropriateness of the negative binomial family.

**Comment:** Regarding control for journal prestige, using a temporally misaligned aggregate indicator and mean imputation may introduce bias and dampen real variability between journals. Ideally, the metric corresponding to the publication year should be used, or journal-by-year fixed effects should be included; if imputation is unavoidable, consider multiple imputations and report a sensitivity analysis without imputation. These steps will make the inferences more robust to measurement choices.

**Response:** We revised our handling of the Article Influence Score (AIS) by removing mean imputation for missing values. Instead, AIS is included as a scaled covariate where available, and journal-level random effects provide robust control for unmeasured journal prestige variation. This approach avoids the bias and variance attenuation concerns associated with single-value imputation. We acknowledge that year-specific AIS values would be ideal; however, our journal random effects capture stable prestige differences, and the year × OA interaction accounts for temporal trends that might otherwise confound the relationship.

**Comment:** The classification of access modalities deserves a specific note. The category commonly labeled bronze may reflect temporary free availability or ambiguous licensing, which could affect the stability of conclusions, primarily if that category consistently performs worse. I recommend validating the classification with a second source and distinguishing temporary free access from permanent availability. This check will reduce the risk of differential misclassification across groups.

**Response:** We agree that the Bronze category is heterogeneous and can include both temporary free access and publisher-hosted content without a clear open license, which raises the possibility of misclassification. In this study, OA status was derived from the Web of Science OA indicators, and Bronze OA was analyzed as a single group because our dataset did not reliably distinguish between time-limited versus permanent free availability or provide consistent license information at the article level. We have now clarified this operational definition in the Methods, as well as added a table defining the Web of Science open access categories, and explicitly noted the heterogeneity of the Bronze category as a limitation. We have also noted in the discussion that the poorer performance of Bronze articles could be due to possible misclassification or heterogeneity of the group.

*Changes to Manuscript:*

*In the Methods:*

- *Original: “Finally, we extracted the OA designation of each article. OA status was categorized into four types: Closed Access, Bronze, Green, and Gold.”*
- *Revised: “Finally, OA status was obtained from the Web of Science OA indicators. Articles were grouped into four categories: Closed Access (no OA indicator), Gold OA, Green OA, and Bronze OA. In this dataset, Gold OA corresponded exclusively to Hybrid OA articles. Because journals in which more than <95% of articles were OA were excluded, fully OA journals did not contribute to the sample.* ***Bronze OA comprised “Free-to-Read” articles that were free to read on the publisher’s site but lacked a specified open license and may represent temporary or longer-term free access.****”*

*In the Discussion:*

- *Original: “Bronze OA, however, consistently showed the lowest citation rates, along with decreasing citation rates over time, as seen in Figure 2A. Although these articles are free to read, they lack a clear open license and are often temporarily available at the publisher’s discretion. This unpredictability limits long-term access and discoverability, reducing citation potential.”*
- *Revised: “Bronze OA, however, consistently showed the lowest citation rates, along with decreasing citation rates over time, as seen in Figure 2A. Although these articles are free to read, they lack a clear open license and are often temporarily available at the publisher’s discretion. This unpredictability may limit long-term access and discoverability and may contribute to lower citation counts.* ***However, given that the Bronze category combines free-to-read arrangements and is more prone to ambiguous licensing and temporary access, some of this underperformance may reflect heterogeneity and misclassification within the group.****”*
- *Addition to limitations: “Second, OA categories were defined using Web of Science and necessarily combined heterogeneous subtypes. Bronze OA likely includes both temporary and stable publisher access, and Gold OA comprises only Hybrid OA articles, with no article-level licence, embargo, or APC data, potentially causing biased comparisons between access groups.”*

**Comment:** In terms of presentation, the text is intelligible but requires editorial revision before acceptance. There are isolated spelling errors and header redundancies, alternation between British and American English that should be standardized, and reference-manager artifacts in the body text that need to be removed, with references normalized to the journal’s style. A careful language edit will resolve these points without affecting the substance.

**Response:** We thank the reviewer for these very important observations. We have extensively edited the manuscript to reflect proper British English, fixed spelling errors, and header redundancies. Additionally, we removed the artifacts from the reference manager and have adjusted the references to reflect PLOS ONE’s style.

The data and code materials are appropriately provided as supporting files, which align with the journal’s policy and are a strong point for reproducibility. As an enhancement, I recommend depositing the materials in a public repository with a DOI, ensuring citability and long-term preservation, and referencing them in the revised manuscript.

In my assessment, this is a study of interest to the community, with a solid data foundation, generally rigorous statistical analysis, and commendable transparency. By strengthening confounding and self-selection mitigation, incorporating an exposure-time offset and robust checks for zero inflation, refining the measurement of journal prestige, and implementing the indicated editorial revisions, the conclusions will be better anchored, and the text will meet the expected standard.

**Reviewer #2 Comments:**

**Comment:** The analysis included top quartile (Q1) orthopedic journals which may limit generalizability; findings may not apply to mid-tier or specialty journals- OA models may differ.

**Response:** We kindly thank you for your robust comments on our manuscript. Our decision to focus on Q1 orthopaedic journals was intentional, as these journals have the highest visibility and are most likely to influence clinical practice. However, we agree that OA uptake and licensing practices may differ in mid-tier or specialty journals and our findings should not be assumed to generalize to those settings. To reflect this, we have expanded our limitations section to acknowledge this point and identify it as an area for future research.

*Changes to Manuscript:*

*In the Discussion:*

- *Original: “Second, our analysis focused solely on publications from top quartile journals, limiting the generalizability of findings to journals with varying prestige and potentially different OA dynamics.”*
- *Revised: “First, because we restricted our sample to top quartile journals and used Web of Science as the sole data source, our findings may be affected by additional bias related to the database’s selective journal coverage. As a result, the findings may not generalize to mid-tier or subspecialty orthopaedic journals, where OA uptake, APC levels, and licensing practices may differ.”*
- *Original: “Expanding analyses to include a broader selection of journals and recent publication data post-2019 would further clarify evolving OA trends and their sustainability.”*
- *Revised: “Expanding analyses to include a broader selection of journals tiers and subspecialities, as well as recent publication data post-2019 would further clarify evolving OA trends and their sustainability. Moreover, examining citation patterns in fully OA orthopaedic journals and comparing them with hybrid and subscription-based journals could provide insight into broader OA trends. Finally, …”*

**Comment:** By excluding journals with >95% OA content, the study omits fully OA journals which could offer insights into broader OA trends.

**Response:** We appreciate this observation and agree that fully OA journals also have an important place in the broader OA landscape. In this study, we intentionally excluded journals with >95% OA content because our primary aim was to compare citation patterns between different OA categories within the same journal, while keeping journal-specific characteristics relatively constant. Fully OA journals typically publish almost exclusively Gold OA content and lack an internal non-OA comparator. For this reason, we focused on hybrid journals where multiple OA types co-exist. To address this, we have expanded upon our reasoning for excluding journals with >95% OA content in the Methods - Journal Identification section. We agree that this design choice limits our ability to describe OA patterns in fully OA journals and have added this to our limitations and emphasized it as an area for future research.

*Changes to Manuscript:*

*In the Methods - Journal Identification:*

- *Original: “Journals were excluded if >95% of their articles were published as OA, to ensure balanced comparison between OA and non-OA articles.”*
- *Revised: “Journals were excluded if >95% of their articles were published as OA, to ensure balanced comparison between OA and non-OA articles within each title. This restriction allowed us to compare different OA designations inside the same journal and reduced the risk of conflating journal-level characteristics with article-level access status.”*

*In the Discussion:*

- *Addition to limitations: “Third, we intentionally excluded journals in which <95% of articles were published as OA, which means that fully OA journals were not represented in our sample. This design choice allowed us to compare OA designations within hybrid journals, but it also limits the extent to which our findings describe broader OA trends across OA titles.”*
- *Addition to future research paragraph: “Moreover, examining citation patterns in fully OA orthopaedic journals and comparing them with hybrid and subscription-based journals could provide insight into broader OA trends.”*

**Comment:** The study is limited to Orthopedics, other surgical or biomedical specialties where OA models, adoption, citing behavior may vary.

**Response:** Thank you for bringing up this point. We agree that OA policies, adoption, and citing behaviour may differ across other surgical and biomedical specialities. Our goal in the present study was not to characterize OA citation patterns across all of medicine, but to provide an analysis with high-impact orthopaedic surgery journals as a single specialty case. We recognize this focus limits generalizability to other specialties and have now made this clearer in the limitations section.

*Changes to Manuscript:*

*In the Discussion:*

- *Addition to limitations: “Fourth, our analysis was restricted to orthopaedic surgery journals, so OA policies, adoption, and citation patterns observed may not generalize to other surgical or biomedical specialties.”*

**Comment:** Downsides of OA categorization

**Comment:** Bronze OA has been treated as a single category. There are subcategories which should not be clubbed. Hybrid OA has not been dealt with, though it finds a mention in the introduction. Gold OA has further categories with different APC.

**Response:** We thank the reviewer for these thoughtful points regarding OA categorization. In our study, OA status was obtained from the Web of Science OA indicators. To address your points regarding subcategories, we have clarified our definitions in the introduction and methods to specify that, in general, Gold OA can occur in either fully OA journals or as hybrid OA articles within subscription journals (but in this database Gold OA corresponds exclusively to Gold Hybrid OA articles, as we excluded journals with <95% OA content from the sample). We have added a table summarizing the Web of Science categories used in the analysis and explicitly note that Bronze OA combines publisher-hosted free-to-read articles with unclear or non-CC licences and may include both temporary and more stable free access. In the limitations section, we now explicitly state that Bronze OA was analysed as a single heterogeneous category, that Gold OA in this study consists only of Hybrid OA articles within subscription journals, and that we did not have article-level data on licences, embargo periods, or APC amounts. We acknowledge that this heterogeneity may bias or obscure differences between OA subtypes and highlight this as an important area for future research.

*Changes to manuscript: We have added a table summarizing the Web of Science OA classifications used in our study, as our data were extracted from this source. Additionally, we have added more text as outlined below.*

*Changes to Manuscript:*

*In the Methods:*

- *“Finally, OA status was obtained from the Web of Science OA indicators. Articles were grouped into four categories: Closed Access (no OA indicator), Gold OA, Green OA, and Bronze OA. In this dataset, Gold OA corresponded exclusively to Hybrid OA articles. Because journals in which more than <95% of articles were OA were excluded, fully OA journals did not contribute to the sample. Bronze OA comprised “Free-to-Read” articles that were free to read on the publisher’s site but lacked a specified open license and may represent temporary or longer-term free access. Green OA combined the “Green-submitted”, “Green-accepted”, and “Green-published” indicators and included self-archived versions of manuscripts in institutional or subject repositories. Definitions of each OA category, as classified in Web of Science, are summarized in Table 1.”*

**Table 1. Definitions of Open Access used in this Study.**

| **OA category** | **Subtype (where applicable)** | **Description*** |
| --- | --- | --- |
| Closed access | - | No open license detected. Full text is only available via subscription or pay-per-view, and the article is not classified as OA in Web of Science/Unpaywall. |
| Gold OA | Fully gold journal | Article is published in a journal where all content is OA and each article has a Creative Commons (CC) license recorded in Unpaywall; the journal meets the Budapest OA Initiative definition of Gold OA. |
|  | Hybrid | Article has a CC license in Unpaywall but is published in a subscription or hybrid journal where only some articles are OA. Classification may be incomplete for newly published content. |
| Bronze OA | Free-to-read | Article is free to read on the publisher’s site, but the license is unclear or non-CC. Access may be promotional or time-limited, and the licensing status may change over time. |
| Green OA | Published version | Version of record deposited in an institutional or subject repository (e.g., an article in PubMed Central after any embargo period). |
|  | Accepted manuscript | Author-accepted manuscript deposited in a repository. The content has passed peer review but may not include the publisher’s copy-editing or typesetting. |
|  | Submitted manuscript | Original submitted version (preprint or pre–peer review manuscript) deposited in a repository prior to formal peer review. |

*Descriptions adapted from Web of Science OA type documentation and materials.

OA: Open Access

**Comment:** The study has equated impact to citations alternative metrics have not been covered. Clinical research takes time to gain visibility and citations shorter citation window may miss some citations. APC data have not been included, the cost-effectiveness analysis has not been done.

**Response:** We thank the reviewer for these points. As outlined in the Introduction and Discussion, our prespecified outcome was citation-based impact, and we acknowledge in the manuscript that citations are only one proxy for impact and that alternative metrics were beyond the scope of this study. We also note in the limitations that our analysis uses citation counts at a single time point; however, all OA models and article types were evaluated over the same window, so the relative associations remain informative within this constraint. Finally, we state that article-level APC data were not available, which precluded a formal cost-effectiveness analysis, and we highlight this as an area for future work rather than a feasible addition to the current study.

**Comment:** Author count estimation relied on simple passing and may miss all authors in instances of “et al”. This may result in bias collaboration analysis.

**Response:** Thank you for your attention to this point. The data we extracted from Web of Science did not use any instances of “et al.” in the author column. We have ensured that all authors are listed throughout extraction and the statistical analysis. We have also attached the S1_File.csv for your reference; however, the author tab has been deleted to respect confidentiality and the number of authors is reflected instead.

**Comment:**  There is bias in the coverage of WoS.

**Response:** Thank you for highlighting this important point. We agree that there is selection bias in Web of Science and have noted this in our limitations.

*Changes to manuscript:*

*In Discussion:*

- *Original: “Second, our analysis focused solely on publications from top quartile journals.”*
- *Revised: “First, because we restricted our sample to top quartile journals and used Web of Science as the sole data source, our findings may be affected by additional bias related to the database’s selective journal coverage.”*

**Comment:**

Confounding factors.

Reviews abstract more citations irrespective OA status. The parameters of funding bodies, universities’ policies also influence OA adoption and ultimately the total number of citations.

Collaboration within the country and at the international level requires more clarity.

Findings should be explained in the context of mandates of funding agency.

**Response:** We thank the reviewer for their important points and have added more clarity to our limitations. We included article type within the analysis to include the article type as a variable instead of a confounding variable.

*Changes to manuscript:*

*In the Discussion:*

- *“In addition, we were unable to systematically account for journal-, funder-, or institution-level OA mandates and policies, which may influence both OA adoption and citation performance and therefore confound the associations observed between OA status and citations.”*

**Comment:** It is stated that “Gold OA provides the greatest visibility and impact”- it conveys a causal relationship. Higher citation rates may show selection bias.

**Response:** Thank you for bringing this point to our attention. We have edited the areas of the manuscript where casual language appears and revised them with associative alternatives. We have also added an acknowledgement of this in the limitations.

*Changes to Manuscript:*

*In the conclusion:*

- *Original: “To address whether "OA pays" in orthopedic surgery publishing, the evidence strongly supports differential benefits by access type. Gold OA provides the highest return on investment with a 3-fold citation advantage over Bronze and 58% more citations than traditional closed access. Green OA offers a middle ground with 19% more citations than closed access. Paradoxically, Bronze access, despite being freely available, performs worst, suggesting that mere accessibility without the quality signals and indexing of Gold/Green routes may actually diminish article impact. The strong effect of collaboration suggests that multi-institutional studies, regardless of access type, achieve greater visibility in the orthopedic literature. For orthopedic researchers and practicing orthopedic surgeons, these findings translate to substantial differences in research impact and can help direct the choice of publication model when disseminating research findings.*
- *Revised: “To address whether "OA pays" in orthopedic surgery publishing, the evidence strongly supports* ***substantial differences in citation counts by access type****. Gold OA* ***had approximately three times the citations of Bronze OA articles*** *and 58% more citations than traditional closed access. Green OA* ***had intermediate citation counts*** *with about 19% more citations than closed access articles. Paradoxically, Bronze access, despite being freely available,* ***had the lowest citation counts****,* ***suggesting free-to-read status alone was not associated with higher citation counts in this sample****. The strong association* ***between author count and citations*** *suggests that multi-institutional studies, regardless of access type,* ***tend to have higher citation counts.*** *For orthopedic researchers and practicing orthopedic surgeons, these findings* ***highlight substantial differences in citation patterns and may inform authors’ decisions*** *about publication models when disseminating research findings.”*

*In the Discussion:*

- *Addition to limitations: “Finally, because this is an observational bibliometric analysis, all findings represent associations rather than causal effects.”*

**Comment:** There is no mention of equity implications of APCs for authors of low and middle income countries. It requires more explanation.

**Response:** We thank the reviewer for highlighting this important point and agree that the equity implications of APC-based models warrant more explicit discussion. We have expanded the Introduction to note how APCs may disproportionately disadvantage authors from low- and middle-income countries and unfunded institutions, and we have added a statement to the Discussion/Limitations explicitly acknowledging that APC-based OA models can exacerbate global inequities in who is able to publish OA.

*Changes to Manuscript:*

*In the Introduction:*

- *Original: “High APCs may deter researchers in low- and middle-income countries from pursuing OA routes despite publisher discounts. These costs may inadvertently reinforce the very inequities OA was intended to overcome.”*
- *Revised: “High APCs may deter researchers in low- and middle-income countries and those at under-resourced institutions from pursuing OA routes, despite publisher discounts. These costs may inadvertently reinforce the very global inequities in who can publish and disseminate research that OA was intended to overcome.”*

*In the Discussion:*

- *Original: N/A*
- *Revised: “Sixth, APC-based OA models may exacerbate inequities in who can publish OA, particularly for authors from low- and middle-income countries or under-resourced institutions. Our analysis could not fully account for these constraints, so the observed citation advantages of OA should be interpreted in light of these access and affordability disparities.”*
