# Supplementary material for: Open access route and citation impact in orthopaedic research: A comparison of Gold/hybrid, Green, and Bronze open access within ten high-impact subscription-based journals
Source: PLoS One. 2026 Nov 4;21(11):e0360532. doi: 10.1371/journal.pone.0360532 (PMC13734443; doi:10.1371/journal.pone.0360532)
Supplement: Supplementary file 3 [file pone.0360532.s012.docx]

February 26^th^, 2026

**Manuscript ID:** PONE-D-25-46173

**Manuscript Title:** Is OA the way? A comparison of different open access classifications and their benefits in orthopaedic research

Dear Reviewers and the editorial team at PLOS ONE,

Thank you so much for your review and thorough feedback of our original manuscript entitled “Is OA the way? A comparison of different open access classifications and their benefits in orthopaedic research” by Daniels et al. We appreciate the effort and hard work of your editorial team and reviewers. Please find attached in the new word document our detailed responses to each comment and our revised manuscript with all changes made and bolded for clarity.

Thank you for your support of our research initiatives. Please advise if you require any further information.

Sincerely,

Michelle Cruickshank, MD (candidate)

Corresponding author: cruicm5@mcmaster.ca

On behalf of co-authors:

William Daniels, MD (candidate)

Taylor Dejong, MD (candidate)

Michelle Ghert, MD, FRCSC

Reviewer #1: Thank you for the revised submission. The manuscript has been improved, and several key points raised in the previous round appear to have been addressed. The statistical approach remains generally appropriate for the study's aims, including the count modeling framework and the diagnostics and robustness checks described. The overall direction of the findings is plausible given the dataset and design.

**Comment:**

A thorough language and formatting review is still needed before publication. The revised files contain visible word processing artifacts, such as “Formatted: …”, typographical errors, and at least one placeholder value in the Methods. Please remove any remaining artifacts, correct typographical and grammar issues, ensure that no placeholders remain in either the manuscript or the response to reviewers document, and confirm that the Methods and Results text is internally consistent and coherent throughout, as PLOS ONE does not copyedit accepted manuscripts.

**Response:**

We thank you for highlighting this important point. We have gone through the manuscript and removed remaining artifacts and fixed the typographical errors previously present. Extensive changes have been made throughout the manuscript and can be seen in the tracked changes document.

**Comment:**

At the same time, I am not yet able to consider the manuscript entirely acceptable as presented because internal inconsistencies and presentation issues remain. The reporting of results should be reconciled across sections so that effect estimates and their interpretation are consistent between the Abstract, the main Results narrative, the Tables and Figures, and the Discussion. The manuscript still reads as if multiple model specifications, such as weighted versus unweighted analyses or primary versus sensitivity analyses, are discussed without a clearly stated hierarchy. Please make explicit which model constitutes the primary basis for inference, clearly label alternative specifications as sensitivity or robustness analyses, and align the narrative conclusions accordingly.

**Response:**

We thank the reviewer for this important clarification. We have revised the Statistical Analysis section to explicitly state that the entropy-balanced negative binomial generalized linear mixed model (GLMM) constitutes the primary basis for inference in this study. The revised manuscript now clearly labels this model as the primary analysis and distinguishes alternative specifications, including unweighted models and non-parametric comparisons, as sensitivity or descriptive analyses only.

We have also ensured that all inferential conclusions throughout the Abstract, Results, Tables, Figures, and Discussion are based exclusively on the weighted negative binomial GLMM. The hierarchy of analyses is now clearly defined in the Methods, and figure captions have been revised where necessary to reflect this distinction. We believe this revision resolves the ambiguity regarding model specification and strengthens the internal consistency of the manuscript.

**Comment:**

The handling and description of AIS and missingness also require more precise and internally consistent reporting. The revised text contains conflicting statements regarding the AIS source and temporal reference, as well as whether missing AIS values were imputed. Please ensure the manuscript and rebuttal are aligned and provide one definitive description of the AIS data used, how missing AIS values were handled, including whether any imputation was applied, and include a brief sensitivity check or an explicit robustness statement showing that the main conclusions do not hinge on the AIS handling choices.

**Response:**

Thank you for bringing the matter of AIS to our attention, we have incorrectly reported in previous versions of this manuscript that AIS were imputed. We can confidently say that AIS values were taken from Scimago for each journal and then joined to the dataframe to make calculations. This means no imputation or assumptions were taken.

**Comment:**

Regarding data availability, the statement indicates that the underlying data are available via supporting information and or a public repository. Please verify that the actual datasets required to reproduce the analyses, not just scripts and a README, are accessible without restriction. If not, clearly specify any limitations and provide an appropriate publicly accessible alternative, such as anonymized datasets deposited in a stable repository with a versioned release or DOI. This is particularly important because the public repository documentation suggests the analysis relies on external input datasets. I have no additional concerns regarding dual publication, research, or publication ethics based on the materials provided. Once the reporting inconsistencies, AIS, and missing data description, presentation artifacts, and data access details are corrected, the manuscript should be suitable for publication.

**Response:**

The data for AIS and for data for the main scripts calculations (along with the .rds that were generated) have been added to the github. The github README was also updated to take into account the new script layout. The new data objects are “AIS2023 … csv”, “min_data.csv”, and “final_weighted_model.rds”. The script required the removal of author names to personal identification purposes so the [main.sh](http://main.sh) will bypass the “01_data_prep.R” script to execute the main analysis “02_analysis.R” script with the given data.

**Comment:**

Reviewer #2: All concerns have been addressed . Language check is required.Links of the datasets should be checked.

**Response:**

Thank you for your time you took to review our manuscript.
