# Supplementary material for: Open access route and citation impact in orthopaedic research: A comparison of Gold/hybrid, Green, and Bronze open access within ten high-impact subscription-based journals
Source: PLoS One. 2026 Nov 4;21(11):e0360532. doi: 10.1371/journal.pone.0360532 (PMC13734443; doi:10.1371/journal.pone.0360532)
Supplement: Supplementary file 5 [file pone.0360532.s014.docx]

Response to Reviewers: PONE-D-25-46173R3 (revised)

**Manuscript:** PONE-D-25-46173R3
**Revised title:** Open access route and citation impact in orthopaedic research: a comparison of Gold/hybrid, Green, and Bronze open access within ten high-impact subscription-based journals
**Journal:** PLOS ONE

Dear Dr. Mukherjee,

Thank you for reviewing out manuscript, we appreciate your time with this paper. We have addressed all ten points.

A short summary of what was changed:

- The paper is now explicitly about comparing OA routes within predominantly subscription/hybrid journals, not about OA publishing in general. This runs through the title, abstract, introduction, discussion, and conclusion.
- The Gold category is called Gold/hybrid OA everywhere now, including every remaining in-text mention in the Methods, Results, and Discussion, and the abstract says that fully OA journals were not represented.
- We added a journal selection flow diagram and a screening log.
- We added the weighting diagnostics that were missing, weight distribution, maximum weights, effective sample sizes, overlap, plus a trimmed-weights sensitivity analysis.
- We split Green OA into its three subtypes and analysed them separately.
- We added a citations-per-year analysis, and said plainly that our main outcome is a cumulative count with unequal follow-up.
- We added a journal-by-year random effect to deal with year-specific journal influence, and now state why year-specific journal metrics could not be substituted directly.
- We picked one set of words and removed the last evaluative terms ("advantage", "performance", "deficit", "benefit").
- The Results are now split into three labelled layers: unadjusted, primary adjusted, and sensitivity.
- We corrected several internal inconsistencies uncovered during this revision (see "A few other things we changed").

Three supplementary figures and four supplementary tables were added. We kept the supplement compact and grouped related results into panels within a single file, rather than splitting every result into its own item. None of our conclusions changed. Section names below refer to the revised manuscript. Everything is visible in the tracked-changes version.

## **Academic Editor**

**Requirement 1: citing specific published works.** Reviewer #2 did not ask us to cite anything in particular, so we have not added citations on that basis.

**Requirement 2: reference list and retracted articles.** We went back through the reference list. It is complete and correctly formatted, none of the 24 references is retracted, and we did not add, remove, or replace any references in this revision.

## **Reviewer #2**

**1. Please provide a reproducible journal selection flowchart showing all candidate journals, and reasons for exclusion, and an explicit rationale for the 2 mainly added journals.**

Thank you for your comment.

We added S1 Fig, a flow diagram covering the whole selection process: journals screened, how many were excluded at each step and why, then the article-level flow. We also added S1 Table, a screening log with one row per journal assessed, giving the SJR, JCR quartile, AIS, percentage OA, whether it was included, and why. Both are generated by a script that ships with the analysis code, so the numbers come straight from the data rather than being typed in by hand.

The Methods previously said two journals were added on senior author recommendation: the *Journal of Bone and Joint Surgery (JBJS-American)* and *Clinical Orthopaedics and Related Research (CORR)*. When we checked that against the actual dataset, it was wrong. Only one journal was added: the *Journal of Bone and Joint Surgery - American*. *CORR* is not in the dataset at all and contributes no articles to any analysis. We have corrected the Methods and we apologize for the error.

*JBJS-American* was suggested by the senior author because of its standing in the specialty, and it also met all three inclusion criteria on its own. So it was not an exception to our criteria, and we now say so. It appears as its own box in S1 Fig.

*Where:* Methods, "Journal identification"; S1 Fig; S1 Table.

**2. …please use "Gold/hybrid OA or hybrid Gold OA" … and write in the abstract that fully OA journals were not represented.**

Thank you for pointing this out. The category is Gold/hybrid OA everywhere now including the title, abstract, results, figures, tables, discussion, and conclusion.

The abstract now says it in the third sentence: "Journals in which more than 95% of articles were OA were excluded by design; fully OA journals were therefore not represented, and every Gold OA article in this corpus was published as hybrid OA within a subscription journal (hereafter Gold/hybrid OA)." And the last sentence returns to it: "Within subscription-based orthopaedic journals, OA routes are not interchangeable; these findings do not extend to fully OA journals." The same point is made in the methods, the limitations, and conclusion.

*Where:* throughout.

**3. Please frame the research question more narrowly as comparing OA routes within predominantly subscription/hybrid high-impact journals.**

Thank you for bringing out attention to this point. We have made the changes accordingly.

The last paragraph of the Introduction now states: "among articles published in high-impact orthopaedic journals that operate on a predominantly subscription or hybrid basis, is the OA route by which an article becomes freely readable associated with its citation rate?" It then says what the study is not: "The study therefore does not compare fully OA journals with subscription journals, and it does not evaluate OA publishing models in general."

The title carries the same restriction. The conclusion opens with "Within ten high-impact orthopaedic journals that publish predominantly on a subscription or hybrid basis…" and closes by saying these findings do not speak to the fully-OA-versus-subscription question. The first limitations also report the same statement.

*Where:* Title; Abstract; Introduction, final paragraph; Discussion; Limitations; Conclusion.

**4. Please report weight distribution, maximum weights, and an overlap … A sensitivity analysis using trimmed/truncated weights would also be useful.**

We have added weight diagnostics, a common-support assessment, and truncated-weight sensitivity analyses.

Weights (S2 Table panel A; S2 Fig A–B). Entropy balancing sets the mean weight to 1.00 in every group. Maximum weights were 11.06 (Gold/hybrid OA), 5.76 (Green OA), 4.02 (Bronze OA), and 3.71 (Closed Access); variability was greatest in Gold/hybrid OA (CV 1.17) and Bronze OA (1.12) and least in Closed Access (0.18). The heaviest 1% of articles carried 3.7% of total weight overall and 9.0% within Gold/hybrid OA. Effective sample size was 19,546 of 24,115 overall (81.1%); by group, 386 of 911 (42.4%) for Gold/hybrid OA, 925 of 2,093 (44.2%) for Bronze OA, 1,431 of 2,187 (65.4%) for Green OA, and 18,343 of 18,924 (96.9%) for Closed Access.

Overlap (S2 Table panel B; S2 Fig C). We assessed common support using generalized propensity scores from a multinomial logistic regression on the covariates used for weighting. Support was good but not complete: 23,011 of 24,115 articles (95.4%) fell within the region where all four categories were represented. Of the 1,104 (4.6%) outside, most (885) lay outside on the Gold/hybrid dimension, 880 of them with a predicted probability of Gold/hybrid OA below the minimum observed within that group. These were low-propensity tails rather than broad separation between groups.

Trimming (S2 Table panel C). We refit the primary model with weights truncated at the 99th, 97.5th, and 95th percentiles and at the 1st/99th. Truncation at the 99th altered 239 weights (1.0%) and reduced the maximum from 11.06 to 2.99. Balance was exact before truncation and remained acceptable at the 99th (largest absolute SMD 0.055) and 97.5th (0.086); at the 95th, two covariates exceeded 0.1 (largest 0.158), so the most aggressive truncation reduces weight variability at the cost of reintroducing imbalance.

Gold/hybrid OA and Green OA versus Closed Access were stable: 1.41 → 1.44, 1.44, 1.44 and 1.35 → 1.34, 1.31, 1.28. The Gold–Green contrast narrowed progressively (p = 0.689, 0.210, 0.065, 0.019), reaching significance only under the 95th-percentile specification, the one with compromised balance. The two-tailed 1st / 99th specification matched the 99th throughout.

Bronze OA versus Closed Access was more sensitive, moving from 1.09 (p = 0.003) to 1.05 (p = 0.269) at the 95th percentile. We report this openly and have removed the directional Bronze claim from the Discussion and Conclusion (see point 10).

***Where***: Methods, "Sensitivity and descriptive analyses"; Results, "Layer 3," paragraphs 1–2; S2 Table; S2 Fig.

**5. If possible, separate Green subtypes and distinguish bronze access duration. If not, mention that the analysis evaluates broad database-derived OA classification rather than homogeneous publishing strategies.**

Thank you for bringing up this point. We were able to do the Green subtypes.

*Green subtypes (S4 Table).* The Green-submitted, Green-accepted, and Green-published flags are still in the raw Web of Science export, so we pulled them back out, re-ran the entropy balancing with a six-level treatment variable, and refit. Green-submitted was 555 articles (2.3%), Green-accepted 823 (3.4%), Green-published 809 (3.4%).

All three had higher citation rates than Closed Access (1.58, 1.26, and 1.17; all p < 0.001), but they were not the same as each other. Green-submitted was higher than Green-accepted (1.25, 95% CI 1.07–1.46) and Green-published (1.35, 95% CI 1.16–1.58), while Green-accepted and Green-published did not differ (p = 0.544). Gold/hybrid OA was higher than Green-published (1.22, 95% CI 1.07–1.39) but did not differ from Green-submitted (p = 0.337) or Green-accepted (p = 0.115).

The ordering is consistent with the embargo account in our Discussion, though we note the subtypes are assigned by most-final version, so Green-published records may also have had earlier repository versions; the contrast is therefore between deposit patterns rather than strictly between access dates. Raw means run in the opposite order, reflecting the higher author counts and journal influence of Green-published articles, which the weighting adjusts for. Collapsing the three subtypes reproduced the original estimates to within ~3%.

*Bronze duration.* Unfortunately, it was not possible with our data, and we now say this plainly in both the Methods and the limitations. The Web of Science OA field is genuinely a one point snapshot from the moment we pulled the data, it does not record how long anything has been free to read. So Bronze OA here means "free to read when we looked", not "durably free", and we could not subdivide it by how long access lasted.

*Database-derived labels.* We took the reviewer's wording directly. The Methods now describe these as "database-derived access labels rather than homogeneous publishing strategies", and the limitations repeat it with numbers. The flags are not mutually exclusive, and our precedence rule (hybrid > Green > Bronze > none, now spelled out in the Methods) puts each article in the most privileged bucket it qualifies for. Concretely: 798 of the 911 Gold/hybrid OA articles (87.6%) also carry a Green flag, and 362 of the 2,187 Green OA articles (16.6%) also carry a Bronze flag. Reporting those overlaps seemed like the most honest way to make the reviewer's point.

*Where:* Methods, "Individual article metadata extraction"; Results, "Layer 3", paragraph 3; Discussion; Limitations; S4 Table.

**6. Consider citation rate per year … or write that the current analysis includes cumulative citation counts with unequal follow-up periods.**

Thank you for bringing this to our attention. We implemented the first suggestion, and for the second we did what the reviewer suggested and stated the limitation.

*Citations per year (S5 Table panels C-D).* Mean citations per year of follow-up were 2.31 for Bronze OA, 4.37 for Closed Access, 6.99 for Green OA, and 9.53 for Gold/hybrid OA — same ordering as the cumulative counts. We also refit the primary model with an offset for log(years since publication), which turns the outcome into a per-year rate rather than a running total. Almost nothing moved: Gold/hybrid OA versus Closed Access 1.41, Green OA versus Closed Access 1.36, Gold/hybrid OA versus Green OA 1.04 (p = 0.689). S5 Table also gives the follow-up window for each cohort.

*Standardised window.* We are unable to build one. The Web of Science export gives a single cumulative citation total per article, not citations year by year, so there is no way to reconstruct something like "citations within three years of publication". We say this in the Methods and again in the limitations, in the terms the reviewer suggested: the counts are cumulative totals at the extraction date, and follow-up ran from roughly five years for the 2019 cohort to roughly ten years for the 2014 cohort. Worth noting that publication year is in the model, and the OA-by-year interaction is one of the paper's actual findings, so the uneven follow-up is modelled rather than ignored.

*Where:* Methods, "Individual article metadata extraction" and "Sensitivity and descriptive analyses"; Results, "Layer 3", paragraph 4; Limitations; S5 Table.

**7. Please use year-specific journal influence metrics corresponding to each publication year.**

Thank you for highlighting this point. We were unable to obtain year-specific Article Influence Scores for all ten journals across 2014-2019, and we now state this explicitly in the Methods to explain why we took a different route rather than year-matching a single index. Rather than swap in a partial or approximate set, we used an approach that we think answers the underlying concern better.

*Journal-by-year random intercept (S5 Table panel E).* We added a random intercept for each journal-year cell. Any year-specific journal metric, AIS, impact factor, SJR, whichever is constant within a journal-year cell by definition, so this term soaks all of them up at once, along with anything else about a journal that changes from year to year (editorial turnover, indexing changes, a themed volume). It is a broader adjustment than plugging in a year-specific value for one index.

It improved fit (AIC 202,564 versus 202,773). The journal-by-year SD was 0.118 across 60 cells, smaller than the between-journal SD of 0.160, which suggests year-to-year movement in journal influence is modest next to the differences between journals. The OA estimates barely moved: Gold/hybrid OA versus Closed Access 1.40, Green OA versus Closed Access 1.31, Gold/hybrid OA versus Green OA 1.07 (p = 0.279).

It is also worth saying that the primary model already had a random slope for publication year by journal, so journal-specific citation trends over time were in there from the start.

We added the single-value AIS to the limitations, along with this result, so a reader sees both the shortcoming and the evidence that it does not drive the findings.

*Where:* Methods, "Individual article metadata extraction" and "Sensitivity and descriptive analyses"; Results, "Layer 3", paragraph 4; Limitations; S5 Table.

**8. Title should be changed. Please mention "citation impact/visibility" rather than general benefits, or include additional impact metrics.**

Thank you for bringing up this point. We have changed it from:

Is OA the way? A comparison of different open access classifications and their benefits in orthopaedic research

It is now:

Open access route and citation impact in orthopaedic research: a comparison of Gold/hybrid, Green, and Bronze open access within ten high-impact subscription-based journals

The new title describes the outcome instead of claiming a benefit, names the categories, and carries the subscription-journal restriction from point 3. We also cut the "Is OA the way?" hook and the "OA pays" line that opened the conclusion to prevent over-generalisation.

*Where:* Title

**9. Please use the same words throughout - instead of advantage, outperform, benefit or impact.**

Thank you for highlighting this point. We picked one vocabulary and said so in the Methods so the reader knows what we mean.

The new "Analytic framework" section states: "Throughout, 'citation rate' denotes the model-estimated expected number of citations for an article and 'citation count' denotes observed totals; differences between OA categories are reported as rate ratios. Evaluative terms such as 'advantage', 'benefit' and 'outperform' are avoided so that a single vocabulary is used throughout."

We made changes throughout the manuscript: "Citation advantage", "citation benefit", "citation performance", "outperformed", "underperformed", "superior citation performance", "the advantage conferred by", "greater citation impact", "apparent Bronze OA deficit", and "OA pays" are all gone, replaced with "had a higher/lower citation rate than" or "had a citation rate x times that of". In this revision we specifically corrected the Discussion opening ("citation advantage" → "higher citation rate"; "citation performance" → "citation rate") and the Layer 1 subheading ("deficit" → "difference"). "Citation impact" survives in the title only, as the general idea the reviewer asked us to name in point 8; inside the paper it is "citation rate" throughout. We fixed the headings to match ("Open access effects by author count" became "Citation rate by author count"; "Effects of collaboration, impact, and document type" became "Author count, journal influence, and document type"), and swapped "journal prestige" for "journal influence" so the AIS is described the same way every time. ("Outperformed" is retained only where it describes model fit of the negative binomial versus the Poisson specification, which is not an OA comparison.)

*Where:* throughout.

**10. Please distinguish between three layers — unadjusted descriptive findings, primary adjusted findings, and sensitivity.**

Thank you for bringing this point to our attention. The layers are defined in the methods, signposted in the results, and carried into the supplement.

The new "Analytic framework" section defines them. Layer 1 is the unadjusted descriptive work, which "describes what is published under each OA label in this corpus and is not adjusted for confounding". Layer 2 is "a single prespecified entropy-balanced negative binomial GLMM, which is the source of every inferential conclusion reported in the Abstract and Conclusion". Layer 3 is the sensitivity analyses, which "vary one modelling decision at a time around the Layer 2 model … and are not used to generate new claims".

The Results now carry three headings to match, and each descriptive paragraph describes the unadjusted citation comparison, for instance, ends "These are unadjusted descriptive comparisons and are not used for inference". The abstract states the structure up front. S5 Table and S3 Fig line up all nine specifications side by side with the primary one marked, so it is obvious at a glance which findings are robust and which are not.

Across all nine specifications, Gold/hybrid OA and Green OA having higher citation rates than Closed Access was stable. Bronze OA versus Closed Access was not: 1.09 in the primary model, 1.05 and non-significant under 95th-percentile truncation, and 0.93, the other direction, with no weighting at all. We removed the directional claim about Bronze from the Discussion and the Conclusion and replaced it with a statement that the direction depends on the specification.

*Where:* Abstract; Methods, "Analytic framework"; Results, three new layer headings plus a five-paragraph "Layer 3" section; Discussion; Conclusion; S5 Table, S3 Fig.

## **A few other things we changed**

*Confidence intervals on the post hoc comparisons.* While building the sensitivity tables we noticed the post hoc CIs were computed with an unadjusted critical value while the p-values next to them were Tukey-adjusted. They now both come from the Tukey-adjusted contrasts. Point estimates and p-values are unchanged; the intervals are a bit wider (Bronze OA versus Closed Access is 1.02-1.16 rather than 1.04-1.14), and nothing about the conclusions changes. One comparison had also been printed with a missing upper limit, which we fixed.

*A directional contradiction in the Discussion.* The first paragraph of the Discussion had retained a definitive statement that Closed Access received 8% fewer citations than Bronze OA (rate ratio 0.92), which contradicted the specification-dependent conclusion stated in the abstract, Layer 3, and Conclusion. We rewrote that sentence so the Discussion, like the rest of the paper, declines to assign a direction to the Bronze OA versus Closed Access comparison.

*Mis-referenced tables and incomplete sentences in Layer 1.* Two Layer 1 cross-references pointed to the wrong tables (the OA-definitions table and the journal-level table) and three sentences had been left incomplete during earlier restructuring, the unadjusted pairwise-test sentence, the articles-only sentence preceding Table 4, and the Fig 1 caption. All cross-references now point to the correct descriptive/composition tables, and all three sentences are complete.

*Figure captions aligned to figure content.* The Fig 2 caption referred to stratification "by document type" that did not match the figure as plotted; the caption now matches the figure. The Fig 1 caption now describes both panels (A: citation distribution by category; B: document-type composition).

*Two Results paragraphs that contradicted each other.* Two consecutive paragraphs in "Reconciliation with the adjusted model" were leftovers from an earlier section order — the first referred in the future tense to model results "reported above" that actually appear below, and the second said the opposite of the first. Merged into one paragraph that makes sense.

*Small grammar fixes,* including an incomplete sentence in the Methods ("Definitions of each OA category, as classified in Web of Science (Table 1)." now reads "Definitions of each OA category, as classified in Web of Science, are given in Table 1.").

*Supporting Information list.* It previously listed only two items and did not match what was actually cited in the text. It now lists everything the paper refers to: three figures, five tables, and the data file. Related results are grouped into lettered panels inside a single table rather than split across separate files, so the supplement stays manageable. The standardised-residual table kept its existing S3 Table label so the in-text cross-reference still works.

Thank you again for your review and time with this manuscript. We hope we can publish this manuscript soon.

Kind regards,
Michelle Cruickshank, on behalf of all authors
