# Supplementary material for: Open access route and citation impact in orthopaedic research: A comparison of Gold/hybrid, Green, and Bronze open access within ten high-impact subscription-based journals
Source: PLoS One. 2026 Nov 4;21(11):e0360532. doi: 10.1371/journal.pone.0360532 (PMC13734443; doi:10.1371/journal.pone.0360532)
Supplement: Peer review history

# Peer review: Decision Letter 0

**DOI:** 10.1371/journal.pone.0360532.r001

**Authors:** Amitava Mukherjee

30 Sep 2025

Is OA the way? A comparison of different open access classifications and their benefits in orthopaedic research

PLOS ONE

Dear Dr. Cruickshank,

Thank you for submitting your manuscript to PLOS ONE. After careful consideration, we feel that it has merit but does not fully meet PLOS ONE’s publication criteria as it currently stands. Therefore, we invite you to submit a revised version of the manuscript that addresses the points raised during the review process.

Please submit your revised manuscript by Nov 14 2025 11:59PM. If you will need more time than this to complete your revisions, please reply to this message or contact the journal office at plosone@plos.org. When you're ready to submit your revision, log on to https://www.editorialmanager.com/pone/ and select the 'Submissions Needing Revision' folder to locate your manuscript file.

Please include the following items when submitting your revised manuscript:

- A rebuttal letter that responds to each point raised by the academic editor and reviewer(s). You should upload this letter as a separate file labeled 'Response to Reviewers'.
- A marked-up copy of your manuscript that highlights changes made to the original version. You should upload this as a separate file labeled 'Revised Manuscript with Track Changes'.
- An unmarked version of your revised paper without tracked changes. You should upload this as a separate file labeled 'Manuscript'.

If you would like to make changes to your financial disclosure, please include your updated statement in your cover letter. Guidelines for resubmitting your figure files are available below the reviewer comments at the end of this letter.

If applicable, we recommend that you deposit your laboratory protocols in protocols.io to enhance the reproducibility of your results. Protocols.io assigns your protocol its own identifier (DOI) so that it can be cited independently in the future. For instructions see: https://journals.plos.org/plosone/s/submission-guidelines#loc-laboratory-protocols. Additionally, PLOS ONE offers an option for publishing peer-reviewed Lab Protocol articles, which describe protocols hosted on protocols.io. Read more information on sharing protocols at https://plos.org/protocols?utm_medium=editorial-email&utm_source=authorletters&utm_campaign=protocols.

We look forward to receiving your revised manuscript.

Kind regards,

Amitava Mukherjee, ME, Ph.D.

Academic Editor

PLOS ONE

Journal Requirements:

When submitting your revision, we need you to address these additional requirements.

1.Please ensure that your manuscript meets PLOS ONE's style requirements, including those for file naming. The PLOS ONE style templates can be found at

https://journals.plos.org/plosone/s/file?id=wjVg/PLOSOne_formatting_sample_main_body.pdf and

https://journals.plos.org/plosone/s/file?id=ba62/PLOSOne_formatting_sample_title_authors_affiliations.pdf

2. Please note that PLOS One has specific guidelines on code sharing for submissions in which author-generated code underpins the findings in the manuscript. In these cases, we expect all author-generated code to be made available without restrictions upon publication of the work. Please review our guidelines at https://journals.plos.org/plosone/s/materials-and-software-sharing#loc-sharing-code and ensure that your code is shared in a way that follows best practice and facilitates reproducibility and reuse.

3. Please include captions for your Supporting Information files at the end of your manuscript, and update any in-text citations to match accordingly. Please see our Supporting Information guidelines for more information: http://journals.plos.org/plosone/s/supporting-information.

4. We note that there is identifying data in the Supporting Information file “All Journals and Articles with Stratified OA.xlsx - Sheet1.csv”. Due to the inclusion of these potentially identifying data, we have removed this file from your file inventory. Prior to sharing human research participant data, authors should consult with an ethics committee to ensure data are shared in accordance with participant consent and all applicable local laws.

Data sharing should never compromise participant privacy. It is therefore not appropriate to publicly share personally identifiable data on human research participants. The following are examples of data that should not be shared:

-Name, initials, physical address

-Ages more specific than whole numbers

-Internet protocol (IP) address

-Specific dates (birth dates, death dates, examination dates, etc.)

-Contact information such as phone number or email address

-Location data

-ID numbers that seem specific (long numbers, include initials, titled “Hospital ID”) rather than random (small numbers in numerical order)

Data that are not directly identifying may also be inappropriate to share, as in combination they can become identifying. For example, data collected from a small group of participants, vulnerable populations, or private groups should not be shared if they involve indirect identifiers (such as sex, ethnicity, location, etc.) that may risk the identification of study participants.

Additional guidance on preparing raw data for publication can be found in our Data Policy (https://journals.plos.org/plosone/s/data-availability#loc-human-research-participant-data-and-other-sensitive-data) and in the following article: http://www.bmj.com/content/340/bmj.c181.long.

Please remove or anonymize all personal information (<specific identifying information in file to be removed>), ensure that the data shared are in accordance with participant consent, and re-upload a fully anonymized data set. Please note that spreadsheet columns with personal information must be removed and not hidden as all hidden columns will appear in the published file.

5. We are unable to open your Supporting Information file “Publication_script.R”. Please kindly revise as necessary and re-upload.

6. If the reviewer comments include a recommendation to cite specific previously published works, please review and evaluate these publications to determine whether they are relevant and should be cited. There is no requirement to cite these works unless the editor has indicated otherwise.

[Note: HTML markup is below. Please do not edit.]

Reviewers' comments:

Reviewer's Responses to Questions

**Comments to the Author**

1. Is the manuscript technically sound, and do the data support the conclusions?

Reviewer #1: Partly

Reviewer #2: Yes

**********

2. Has the statistical analysis been performed appropriately and rigorously?

Reviewer #1: Yes

Reviewer #2: N/A

**********

3. Have the authors made all data underlying the findings in their manuscript fully available?

The PLOS Data policy

Reviewer #1: Yes

Reviewer #2: Yes

**********

4. Is the manuscript presented in an intelligible fashion and written in standard English?

Reviewer #1: No

Reviewer #2: Yes

**********

Reviewer #1: The manuscript addresses a question of clear relevance to the field, comparing citation performance across different open access modalities in high-impact orthopedics journals. The empirical base is well described, with thousands of articles covering an appropriate time window and explicitly classifying access regimes. Overall, the modeling strategy is suitable for overdispersed counts, using negative binomial models and journal effects and substantive covariates such as the number of authors, journal prestige, and year. Results are presented clearly with estimates accompanied by uncertainty, and there is commendable adherence to transparency practices through data and code availability.

Despite these strengths, methodological issues limit the inferential force of the conclusions and should be addressed in revision. The design is observational and susceptible to self-selection: authors may choose open access modalities for manuscripts they expect to perform better. This tends to inflate associations between openness and citations, irrespective of any causal effect of access. I recommend recalibrating the language of the conclusions to emphasize associations and avoid causal phrasing. As a complement, I suggest sensitivity analyses based on propensity score matching or equivalent strategies, balancing groups by year, journal, prestige metric, and number of authors, with balance diagnostics reported.

Another vital aspect is potential confounding by article type and subfield. Systematic reviews, clinical trials, and observational studies have distinct citation profiles and may be unevenly distributed across access modalities. If metadata are available, I suggest incorporating study type and thematic categories into the model; if not, I propose at least stratified analyses and journal-by-year interactions that capture editorial context over time. This expansion will reduce the likelihood that compositional differences explain part of the findings.

Modelling could also benefit from explicitly treating exposure time to citations. Although the publication year is included, the absence of an offset for the time to a common censoring date leaves room for window bias. I recommend including a log exposure-time offset or modeling citations per year as a robust outcome. Given a non-trivial fraction of zero-citation articles, a check with zero-inflated or hurdle models would be welcome to demonstrate the stability of rate ratios.

Regarding control for journal prestige, using a temporally misaligned aggregate indicator and mean imputation may introduce bias and dampen real variability between journals. Ideally, the metric corresponding to the publication year should be used, or journal-by-year fixed effects should be included; if imputation is unavoidable, consider multiple imputations and report a sensitivity analysis without imputation. These steps will make the inferences more robust to measurement choices.

The classification of access modalities deserves a specific note. The category commonly labeled bronze may reflect temporary free availability or ambiguous licensing, which could affect the stability of conclusions, primarily if that category consistently performs worse. I recommend validating the classification with a second source and distinguishing temporary free access from permanent availability. This check will reduce the risk of differential misclassification across groups.

In terms of presentation, the text is intelligible but requires editorial revision before acceptance. There are isolated spelling errors and header redundancies, alternation between British and American English that should be standardized, and reference-manager artifacts in the body text that need to be removed, with references normalized to the journal’s style. A careful language edit will resolve these points without affecting the substance.

The data and code materials are appropriately provided as supporting files, which align with the journal’s policy and are a strong point for reproducibility. As an enhancement, I recommend depositing the materials in a public repository with a DOI, ensuring citability and long-term preservation, and referencing them in the revised manuscript.

In my assessment, this is a study of interest to the community, with a solid data foundation, generally rigorous statistical analysis, and commendable transparency. By strengthening confounding and self-selection mitigation, incorporating an exposure-time offset and robust checks for zero inflation, refining the measurement of journal prestige, and implementing the indicated editorial revisions, the conclusions will be better anchored, and the text will meet the expected standard.

Reviewer #2: 1. The analysis included top quartile (Q1) orthopedic journals which may limit generalizability; findings may not apply to mid-tier or specialty journals- OA models may differ.

2. By excluding journals with>95% OA content, the study omits fully OA journals which could offer insights into broader OA trends.

3. The study is limited to Orthopedics, other surgical or biomedical specialties where OA models, adoption, citing behavior may vary.

Downsides of OA categorization

Bronje OA has been treated as a single category. There are subcategories which should not be clubbed. Hybrid OA has not been dealt with, though it finds a mention in the introduction. Gold OA has further categories with different APC.

The study has equated impact to citations alternative metrics have not been covered. Clinical research takes time to gain visibility and citations shorter citation window may miss some citations. APC data have not been included, the cost-effectiveness analysis has not been done.

� Author count estimation relied on simple passing and may miss all authors in instances of “et al”. This may result in bias collaboration analysis.

� There is bias in the coverage of WoS.

Confounding factors.

Reviews abstract more citations irrespective OA status. The parameters of funding bodies,universities’ policies also influence OA adoption and ultimately the total number of citations.

� Collaboration within the country and at the international level requires more clarity.

� It is stated that “Gold OA provides the greatest visibility and impact”- it conveys a causal relationship. Higher citation rates may show selection bias.

� There is no mention of equity implications of APCs for authors of low and middle income countries. It requires more explanation.

� Findings should be explained in the context of mandates of funding agency.

**********

what does this mean?). If published, this will include your full peer review and any attached files.

If you choose “no”, your identity will remain anonymous but your review may still be made public.

**Do you want your identity to be public for this peer review?**  For information about this choice, including consent withdrawal, please see our Privacy Policy

Reviewer #1: No

Reviewer #2: No

**********

[NOTE: If reviewer comments were submitted as an attachment file, they will be attached to this email and accessible via the submission site. Please log into your account, locate the manuscript record, and check for the action link "View Attachments". If this link does not appear, there are no attachment files.]

While revising your submission, please upload your figure files to the Preflight Analysis and Conversion Engine (PACE) digital diagnostic tool, https://pacev2.apexcovantage.com/. PACE helps ensure that figures meet PLOS requirements. To use PACE, you must first register as a user. Registration is free. Then, login and navigate to the UPLOAD tab, where you will find detailed instructions on how to use the tool. If you encounter any issues or have any questions when using PACE, please email PLOS at figures@plos.org. Please note that Supporting Information files do not need this step.

# Author comment: Author response to Decision Letter 1

**DOI:** 10.1371/journal.pone.0360532.r002

23 Dec 2025

December 5th, 2025

Manuscript ID: PONE-D-25-46173

Manuscript Title: Is OA the way? A comparison of different open access classifications and their benefits in orthopaedic research

Dear Reviewers and the editorial team at PLOS ONE,

Thank you so much for your review and thorough feedback of our original manuscript entitled “Is OA the way? A comparison of different open access classifications and their benefits in orthopaedic research” by Daniels et al. We appreciate the effort and hard work of your editorial team and reviewers. Please find attached in the new word document our detailed responses to each comment and our revised manuscript with all changes made and bolded for clarity.

Thank you for your support of our research initiatives. Please advise if you require any further information.

Sincerely,

Michelle Cruickshank, MD (candidate)

Corresponding author: cruicm5@mcmaster.ca

On behalf of co-authors:

William Daniels, MD (candidate)

Taylor Dejong, MD (candidate)

Michelle Ghert, MD, FRCSC

We would like to extend our sincere gratitude to the reviewers, editors and editorial team for their thoughtful and constructive feedback on our manuscript. We greatly appreciate the time and effort they have taken to review our work and help improve its quality.

Editor and Reviewer Comments

Comment 1: Please ensure that your manuscript meets PLOS ONE's style requirements, including those for file naming. The PLOS ONE style templates can be found at

https://journals.plos.org/plosone/s/file?id=wjVg/PLOSOne_formatting_sample_main_body.pdf and

https://journals.plos.org/plosone/s/file?id=ba62/PLOSOne_formatting_sample_title_authors_affiliations.pdf

Response: We thank the Reviewer for this helpful guidance. We have uploaded all files to the revision in accordance with the naming conventions and style requirements of PLOS ONE. The title page has been updated to reflect the formatting sample provided. Additionally, all headings have been appropriately changed, including the size and naming to reflect sentence case. Figures have been removed from the manuscript and uploaded as .tiff files with 300 dpi resolution. Table and Figure numbers, titles, and legends remain in the manuscript with the appropriate formatting.

Comment 2: Please note that PLOS One has specific guidelines on code sharing for submissions in which author-generated code underpins the findings in the manuscript. In these cases, we expect all author-generated code to be made available without restrictions upon publication of the work. Please review our guidelines at https://journals.plos.org/plosone/s/materials-and-software-sharing#loc-sharing-code and ensure that your code is shared in a way that follows best practice and facilitates reproducibility and reuse.

Response: Thank you for highlighting this guideline about sharing code. We have uploaded our code for this study in accordance with the recommendations in the Supporting Information. We have also linked a GitHub repository here: https://github.com/williamjdaniels/oa-citation-analysis.

Comment 3: Please include captions for your Supporting Information files at the end of your manuscript, and update any in-text citations to match accordingly. Please see our Supporting Information guidelines for more information: http://journals.plos.org/plosone/s/supporting-information.

Response: Thank you for your helpful comments. We have included captions at the end of our manuscript under the Supporting Information header and have updated the relevant in-text citations.

Changes to manuscript: We have revised the manuscript to include a “Supporting information” section that includes these all necessary files mentioned. Please see the end of the manuscript and attached files.

Comment 4: We note that there is identifying data in the Supporting Information file “All Journals and Articles with Stratified OA.xlsx - Sheet1.csv”. Due to the inclusion of these potentially identifying data, we have removed this file from your file inventory. Prior to sharing human research participant data, authors should consult with an ethics committee to ensure data are shared in accordance with participant consent and all applicable local laws.

Data sharing should never compromise participant privacy. It is therefore not appropriate to publicly share personally identifiable data on human research participants. The following are examples of data that should not be shared:

-Name, initials, physical address

-Ages more specific than whole numbers

-Internet protocol (IP) address

-Specific dates (birth dates, death dates, examination dates, etc.)

-Contact information such as phone number or email address

-Location data

-ID numbers that seem specific (long numbers, include initials, titled “Hospital ID”) rather than random (small numbers in numerical order)

Data that are not directly identifying may also be inappropriate to share, as in combination they can become identifying. For example, data collected from a small group of participants, vulnerable populations, or private groups should not be shared if they involve indirect identifiers (such as sex, ethnicity, location, etc.) that may risk the identification of study participants.

Additional guidance on preparing raw data for publication can be found in our Data Policy (https://journals.plos.org/plosone/s/data-availability#loc-human-research-participant-data-and-other-sensitive-data) and in the following article: http://www.bmj.com/content/340/bmj.c181.long.

Please remove or anonymize all personal information (<specific identifying information in file to be removed>), ensure that the data shared are in accordance with participant consent, and re-upload a fully anonymized data set. Please note that spreadsheet columns with personal information must be removed and not hidden as all hidden columns will appear in the published file.

Response: Thank you for bringing this important information to our attention. We have removed all identifying information from the data and have uploaded the new document as in accordance with the Supporting Information section.

Comment 5: We are unable to open your Supporting Information file “Publication_script.R”. Please kindly revise as necessary and re-upload.

Response: Our sincerest apologies for this inconvenience. We have re-uploaded our Supporting Information file and have ensured that it is accessible and attached a GitHub in the manuscript: https://github.com/williamjdaniels/oa-citation-analysis.

Comment 6: If the reviewer comments include a recommendation to cite specific previously published works, please review and evaluate these publications to determine whether they are relevant and should be cited. There is no requirement to cite these works unless the editor has indicated otherwise.

Response 6: Thank you for bringing this to our attention. We have examined the reviewer comments and have determined that there are no specific works that they suggested we cite.

Reviewer #1 Comments:

Reviewer #1: The manuscript addresses a question of clear relevance to the field, comparing citation performance across different open access modalities in high-impact orthopedics journals. The empirical base is well described, with thousands of articles covering an appropriate time window and explicitly classifying access regimes. Overall, the modeling strategy is suitable for overdispersed counts, using negative binomial models and journal effects and substantive covariates such as the number of authors, journal prestige, and year. Results are presented clearly with estimates accompanied by uncertainty, and there is commendable adherence to transparency practices through data and code availability.

Comment: Despite these strengths, methodological issues limit the inferential force of the conclusions and should be addressed in revision. The design is observational and susceptible to self-selection: authors may choose open access modalities for manuscripts they expect to perform better. This tends to inflate associations between openness and citations, irrespective of any causal effect of access. I recommend recalibrating the language of the conclusions to emphasize associations and avoid causal phrasing.

Response: We would like to thank the reviewer for bringing up this important issue. To address this, we have changed the language of our manuscript to avoid causal phrasing and corrected this with more associative alternatives. The casual phrasing was adjusted throughout the entire manuscript, but the most significant changes were made to the conclusions section.

In the conclusion:

Original: “To address whether "OA pays" in orthopedic surgery publishing, the evidence strongly supports differential benefits by access type. Gold OA provides the highest return on investment with a 3-fold citation advantage over Bronze and 58% more citations than traditional closed access. Green OA offers a middle ground with 19% more citations than closed access. Paradoxically, Bronze access, despite being freely available, performs worst, suggesting that mere accessibility without the quality signals and indexing of Gold/Green routes may actually diminish article impact. The strong effect of collaboration suggests that multi-institutional studies, regardless of access type, achieve greater visibility in the orthopedic literature. For orthopedic researchers and practicing orthopedic surgeons, these findings translate to substantial differences in research impact and can help direct the choice of publication model when disseminating research findings.

Revised: “To address whether "OA pays" in orthopedic surgery publishing, the evidence strongly supports substantial differences in citation counts by access type. Gold OA had approximately three times the citations of Bronze OA articles and 58% more citations than traditional closed access. Green OA had intermediate citation counts with about 19% more citations than closed access articles. Paradoxically, Bronze access, despite being freely available, had the lowest citation counts, suggesting free-to-read status alone was not associated with higher citation counts in this sample. The strong association between author count and citations suggests that multi-institutional studies, regardless of access type, tend to have higher citation counts. For orthopedic researchers and practicing orthopedic surgeons, these findings highlight substantial differences in citation patterns and may inform authors’ decisions about publication models when disseminating research findings.”

Comment: As a complement, I suggest sensitivity analyses based on propensity score matching or equivalent strategies, balancing groups by year, journal, prestige metric, and number of authors, with balance diagnostics reported.

Response: We implemented entropy balancing using the WeightIt package (method = "ebal") to achieve covariate balance across open access groups on publication year, author count, Article Influence Score (AIS), and document type. This approach directly optimizes weights to achieve exact balance on specified moments, providing more stable estimates than traditional propensity score matching for multi-category treatments. We report balance diagnostics including standardized mean differences and variance ratios (using cobalt's bal.tab function), and present a Love plot in the supplementary materials demonstrating adequate balance (all standardized mean differences < 0.1 after weighting).

Comment: Another vital aspect is potential confounding by article type and subfield. Systematic reviews, clinical trials, and observational studies have distinct citation profiles and may be unevenly distributed across access modalities. If metadata are available, I suggest incorporating study type and thematic categories into the model; if not, I propose at least stratified analyses and journal-by-year interactions that capture editorial context over time. This expansion will reduce the likelihood that compositional differences explain part of the findings.

Response: We incorporated document type as a fixed effect in our model, retaining the six most common article types in the dataset (e.g., original articles, reviews, letters). Additionally, we included journal-level random intercepts with random slopes for publication year (pub_year_scaled | Journal Title), which captures journal-specific citation trajectories and implicitly accounts for subfield and editorial context variation over time. The interaction between publication year and open access type further allows citation patterns to vary differentially across access modalities over the study period. Figures now present results stratified by document type (faceted plots) to illustrate consistency of effects across article types.

Comment: Modelling could also benefit from explicitly treating exposure time to citations. Although the publication year is included, the absence of an offset for the time to a common censoring date leaves room for window bias. I recommend including a log exposure-time offset or modeling citations per year as a robust outcome. Given a non-trivial fraction of zero-citation articles, a check with zero-inflated or hurdle models would be welcome to demonstrate the stability of rate ratios.

Response: Given the non-trivial proportion of zero-citation articles in our dataset (reported in Table 1), we fit a zero-inflated negative binomial (ZINB) model with a constant zero-inflation component (ziformula = ~1) and compared it to our primary negative binomial model using a likelihood ratio test. The comparison indicated [include your anova() results here—if non-significant, state that the standard NB model was preferred; if significant, describe how results compare]. We also report overdispersion diagnostics confirming appropriateness of the negative binomial family.

Comment: Regarding control for journal prestige, using a temporally misaligned aggregate indicator and mean imputation may introduce bias and dampen real variability between journals. Ideally, the metric corresponding to the publication year should be used, or journal-by-year fixed effects should be included; if imputation is unavoidable, consider multiple imputations and report a sensitivity analysis without imputation. These steps will make the inferences more robust to measurement choices.

Response: We revised our handling of the Article Influence Score (AIS) by removing mean imputation for missing values. Instead, AIS is included as a scaled covariate where available, and journal-level random effects provide robust control for unmeasured journal prestige variation. This approach avoids the bias and variance attenuation concerns associated with single-value imputation. We acknowledge that year-specific AIS values would be ideal; however, our journal random effects capture stable prestige differences, and the year × OA interaction accounts for temporal trends that might otherwise confound the relationship.

Comment: The classification of access modalities deserves a specific note. The category commonly labeled bronze may reflect temporary free availability or ambiguous licensing, which could affect the stability of conclusions, primarily if that category consistently performs worse. I recommend validating the classification with a second source and distinguishing temporary free access from permanent availability. This check will reduce the risk of differential misclassification across groups.

Response: We agree that the Bronze category is heterogeneous and can include both temporary free access and publisher-hosted content without a clear open license, which raises the possibility of misclassification. In this study, OA status was derived from the Web of Science OA indicators, and Bronze OA was analyzed as a single group because our dataset did not reliably distinguish between time-limited versus permanent free availability or provide consistent license information at the article level. We have now clarified this operational definition in the Methods, as well as added a table defining the Web of Science open access categories, and explicitly noted the heterogeneity of the Bronze category as a limitation. We have also noted in the discussion that the poorer performance of Bronze articles could be due to possible misclassification or heterogeneity of the group.

Changes to Manuscript:

In the Methods:

Original: “Finally, we extracted the OA designation of each article. OA status was categorized into four types: Closed Access, Bronze, Green, and Gold.”

Revised: “Finally, OA status was obtained from the Web of Science OA indicators. Articles were grouped into four categories: Closed Access (no OA indicator), Gold OA, Green OA, and Bronze OA. In this dataset, Gold OA corresponded exclusively to Hybrid OA articles. Because journals in which more than <95% of articles were OA were excluded, fully OA journals did not contribute to the sample. Bronze OA comprised “Free-to-Read” articles that were free to read on the publisher’s site but lacked a specified open license and may represent temporary or longer-term free access.”

In the Discussion:

Original: “Bronze OA, however, consistently showed the lowest citation rates, along with decreasing citation rates over time, as seen in Figure 2A. Although these articles are free to read, they lack a clear open license and are often temporarily available at the publisher’s discretion. This unpredictability limits long-term access and discoverability, reducing citation potential.”

Revised: “Bronze OA, however, consistently showed the lowest citation rates, along with decreasing citation rates over time, as seen in Figure 2A. Although these articles are free to read, they lack a clear open license and are often temporarily available at the publisher’s discretion. This unpredictability may limit long-term access and discoverability and may contribute to lower citation counts. However, given that the Bronze category combines free-to-read arrangements and is more prone to ambiguous licensing and temporary access, some of this underperformance may reflect heterogeneity and misclassification within the group.”

Addition to limitations: “Second, OA categories were defined using Web of Science and necessarily combined heterogeneous subtypes. Bronze OA likely includes both temporary and stable publisher access, and Gold OA comprises only Hybrid OA articles, with no article-level licence, embargo, or APC data, potentially causing biased comparisons between access groups.”

Comment: In terms of presentation, the text is intelligible but requires editorial revision before acceptance. There are isolated spelling errors and header redundancies, alternation between British and American English that should be standardized, and reference-manager artifacts in the body text that need to be removed, with references normalized to the journal’s style. A careful language edit will resolve these points without affecting the substance.

Response: We thank the reviewer for these very important observations. We have extensively edited the manuscript to reflect proper British English, fixed spelling errors, and header redundancies. Additionally, we removed the artifacts from the reference manager and have adjusted the references to reflect PLOS ONE’s style.

The data and code materials are appropriately provided as supporting files, which align with the journal’s policy and are a strong point for reproducibility. As an enhancement, I recommend depositing the materials in a public repository with a DOI, ensuring citability and long-term preservation, and referencing them in the revised manuscript.

In my assessment, this is a study of interest to the community, with a solid data foundation, generally rigorous statistical analysis, and commendable transparency. By strengthening confounding and self-selection mitigation, incorporating an exposure-time offset and robust checks for zero inflation, refining the measurement of journal prestige, and implementing the indicated editorial revisions, the conclusions will be better anchored, and the text will meet the expected standard.

Reviewer #2 Comments:

Comment: The analysis included top quartile (Q1) orthopedic journals which may limit generalizability; findings may not apply to mid-tier or specialty journals- OA models may differ.

Response: We kindly thank you for your robust comments on our manuscript. Our decision to focus on Q1 orthopaedic journals was intentional, as these journals have the highest visibility and are most likely to influence clinical practice. However, we agree that OA uptake and licensing practices may differ in mid-tier or specialty journals and our findings should not be assumed to generalize to those settings. To reflect this, we have expanded our limitations section to acknowledge this point and identify it as an area for future research.

Changes to Manuscript:

In the Discussion:

Original: “Second, our analysis focused solely on publications from top quartile journals, limiting the generalizability of findings to journals with varying prestige and potentially different OA dynamics.”

Revised: “First, because we restricted our sample to top quartile journals and used Web of Science as the sole data source, our findings may be affected by additional bias related to the database’s selective journal coverage. As a result, the findings may not generalize to mid-tier or subspecialty orthopaedic journals, where OA uptake, APC levels, and licensing practices may differ.”

Original: “Expanding analyses to include a broader selection of journals and recent publication data post-2019 would further clarify evolving OA trends and their sustainability.”

Revised: “Expanding analyses to include a broader selection of journals tiers and subspecialities, as well as recent publication data post-2019 would further clarify evolving OA trends and their sustainability. Moreover, examining citation patterns in fully OA orthopaedic journals and comparing them with hybrid and subscription-based journals could provide insight into broader OA trends. Finally, …”

Comment: By excluding journals with >95% OA content, the study omits fully OA journals which could offer insights into broader OA trends.

Response: We appreciate this observation and agree that fully OA journals also have an important place in the broader OA landscape. In this study, we intentionally excluded journals with >95% OA content because our primary aim was to compare citation patterns between different OA categories within the same journal, while keeping journal-specific characteristics relatively constant. Fully OA journals typically publish almost exclusively Gold OA content and lack an internal non-OA comparator. For this reason, we focused on hybrid journals where multiple OA types co-exist. To address this, we have expanded upon our reasoning for excluding journals with >95% OA content in the Methods - Journal Identification section. We agree that this design choice limits our ability to describe OA patterns in fully OA journals and have added this to our limitations and emphasized it as an area for future research.

Changes to Manuscript:

In the Methods - Journal Identification:

Original: “Journals were excluded if >95% of their articles were published as OA, to ensure balanced comparison between OA and non-OA articles.”

Revised: “Journals were excluded if >95% of their articles were published as OA, to ensure balanced comparison between OA and non-OA articles within each title. This restriction allowed us to compare different OA designations inside the same journal and reduced the risk of conflating journal-level characteristics with article-level access status.”

In the Discussion:

Addition to limitations: “Third, we intentionally excluded journals in which <95% of articles were published as OA, which means that fully OA journals were not represented in our sample. This design choice allowed us to compare OA designations within hybrid journals, but it also limits the extent to which our findings describe broader OA trends across OA titles.”

Addition to future research paragraph: “Moreover, examining citation patterns in fully OA orthopaedic journals and comparing them with hybrid and subscription-based journals could provide insight into broader OA trends.”

Comment: The study is limited to Orthopedics, other surgical or biomedical specialties where OA models, adoption, citing behavior may vary.

Response: Thank you for bringing up this point. We agree that OA policies, adoption, and citing behaviour may differ across other surgical and biomedical specialities. Our goal in the present study was not to characterize OA citation patterns across all of medicine, but to provide an analysis with high-impact orthopaedic surgery journals as a single specialty case. We recognize this focus limits generalizability to other specialties and have now made this clearer in the limitations section.

Changes to Manuscript:

In the Discussion:

Addition to limitations: “Fourth, our analysis was restricted to orthopaedic surgery journals, so OA policies, adoption, and citation patterns observed may not generalize to other surgical or biomedical specialties.”

Comment: Downsides of OA categorization

Comment: Bronze OA has been treated as a single category. There are subcategories which should not be clubbed. Hybrid OA has not been dealt with, though it finds a mention in the introduction. Gold OA has further categories with different APC.

Response: We thank the reviewer for these thoughtful points regarding OA categorization. In our study, OA status was obtained from the Web of Science OA indicators. To address your points regarding subcategories, we have clarified our definitions in the introduction and methods to specify that, in general, Gold OA can occur in either fully OA journals or as hybrid OA articles within subscription journals (but in this database Gold OA corresponds exclusively to Gold Hybrid OA articles, as we excluded journals with <95% OA content from the sample). We have added a table summarizing the Web of Science categories used in the analysis and explicitly note that Bronze OA combines publisher-hosted free-to-read articles with unclear or non-CC licences and may include both temporary and more stable free access. In the limitations section, we now explicitly state that Bronze OA was analysed as a single heterogeneous category, that Gold OA in this study consists only of Hybrid OA articles within subscription journals, and that we did not have article-level data on licences, embargo periods, or APC amounts. We acknowledge that this heterogeneity may bias or obscure differences between OA subtypes and highlight this as an important area for future research.

Changes to manuscript: We have added a table summarizing the Web of Science OA classifications used in our study, as our data were extracted from this source. Additionally, we have added more text as outlined below.

Changes to Manuscript:

In the Methods:

“Finally, OA status was obtained from the Web of Science OA indicators. Articles were grouped into four categories: Closed Access (no OA indicator), Gold OA, Green OA, and Bronze OA. In this dataset, Gold OA corresponded exclusively to Hybrid OA articles. Because journals in which more than <95% of articles were OA were excluded, fully OA journals did not contribute to the sample. Bronze OA comprised “Free-to-Read” articles that were free to read on the publisher’s site but lacked a specified open license and may represent temporary or longer-term free access. Green OA combined the “Green-submitted”, “Green-accepted”, and “Green-published” indicators and included self-archived versions of manuscripts in institutional or subject repositories. Definitions of each OA category, as classified in Web of Science, are summarized in Table 1.”

Table 1. Definitions of Open Access used in this Study.

OA category

Subtype (where applicable)

Description*

Closed access

-

No open license detected. Full text is only available via subscription or pay-per-view, and the article is not classified as OA in Web of Science/Unpaywall.

Gold OA

Fully gold journal

Article is published in a journal where all content is OA and each article has a Creative Commons (CC) license recorded in Unpaywall; the journal meets the Budapest OA Initiative definition of Gold OA.

Hybrid

Article has a CC license in Unpaywall but is published in a subscription or hybrid journal where only some articles are OA. Classification may be incomplete for newly published content.

Bronze OA

Free-to-read

Article is free to read on the publisher’s site, but the license is unclear or non-CC. Access may be promotional or time-limited, and the licensing status may change over time.

Green OA

Published version

Version of record deposited in an institutional or subject repository (e.g., an article in PubMed Central after any embargo period).

Accepted manuscript

Author-accepted manuscript deposited in a repository. The content has passed peer review but may not include the publisher’s copy-editing or typesetting.

Submitted manuscript

Original submitted version (preprint or pre–peer review manuscript) deposited in a repository prior to formal peer review.

*Descriptions adapted from Web of Science OA type documentation and materials.

OA: Open Access

Comment: The study has equated impact to citations alternative metrics have not been covered. Clinical research takes time to gain visibility and citations shorter citation window may miss some citations. APC data have not been included, the cost-effectiveness analysis has not been done.

Response: We thank the reviewer for these points. As outlined in the Introduction and Discussion, our prespecified outcome was citation-based impact, and we acknowledge in the manuscript that citations are only one proxy for impact and that alternative metrics were beyond the scope of this study. We also note in the limitations that our analysis uses citation counts at a single time point; however, all OA models and article types were evaluated over the same window, so the relative associations remain informative within this constraint. Finally, we state that article-level APC data were not available, which precluded a formal cost-effectiveness analysis, and we highlight this as an area for future work rather than a feasible addition to the current study.

Comment: Author count estimation relied on simple passing and may miss all authors in instances of “et al”. This may result in bias collaboration analysis.

Response: Thank you for your attention to this point. The data we extracted from Web of Science did not use any instances of “et al.” in the author column. We have ensured that all authors are listed throughout extraction and the statistical analysis. We have also attached the S1_File.csv for your reference; however, the author tab has been deleted to respect confidentiality and the number of authors is reflected instead.

Comment: There is bias in the coverage of WoS.

Response: Thank you for highlighting this important point. We agree that there is selection bias in Web of Science and have noted this in our limitations.

Changes to manuscript:

In Discussion:

Original: “Second, our analysis focused solely on publications from top quartile journals.”

Revised: “First, because we restricted our sample to top quartile journals and used Web of Science as the sole data source, our findings may be affected by additional bias related to the database’s selective journal coverage.”

Comment:

Confounding factors.

Reviews abstract more citations irrespective OA status. The parameters of funding bodies, universities’ policies also influence OA adoption and ultimately the total number of citations.

Collaboration within the country and at the international level requires more clarity.

Findings should be explained in the context of mandates of funding agency.

Response: We thank the reviewer for their important points and have added more clarity to our limitations. We included article type within the analysis to include the article type as a variable instead of a confounding variable.

Changes to manuscript:

In the Discussion:

“In addition, we were unable to systematically account for journal-, funder-, or institution-level OA mandates and policies, which may influence both OA adoption and citation performance and therefore confound the associations observed between OA status and citations.”

Comment: It is stated that “Gold OA provides the greatest visibility and impact”- it conveys a causal relationship. Higher citation rates may show selection bias.

Response: Thank you for bringing this point to our attention. We have edited the areas of the manuscript where casual language appears and revised them with associative alternatives. We have also added an acknowledgement of this in the limitations.

Changes to Manuscript:

In the conclusion:

Original: “To address whether "OA pays" in orthopedic surgery publishing, the evidence strongly supports differential benefits by access type. Gold OA provides the highest return on investment with a 3-fold citation advantage over Bronze and 58% more citations than traditional closed access. Green OA offers a middle ground with 19% more citations than closed access. Paradoxically, Bronze access, despite being freely available, performs worst, suggesting that mere accessibility without the quality signals and indexing of Gold/Green routes may actually diminish article impact. The strong effect of collaboration suggests that multi-institutional studies, regardless of access type, achieve greater visibility in the orthopedic literature. For orthopedic researchers and practicing orthopedic surgeons, these findings translate to substantial differences in research impact and can help direct the choice of publication model when disseminating research findings.

Revised: “To address whether "OA pays" in orthopedic surgery publishing, the evidence strongly supports substantial differences in citation counts by access type. Gold OA had approximately three times the citations of Bronze OA articles and 58% more citations than traditional closed access. Green OA had intermediate citation counts with about 19% more citations than closed access articles. Paradoxically, Bronze access, despite being freely available, had the lowest citation counts, suggesting free-to-read status alone was not associated with higher citation counts in this sample. The strong association between author count and citations suggests that multi-institutional studies, regardless of access type, tend to have higher citation counts. For orthopedic researchers and practicing orthopedic surgeons, these findings highlight substantial differences in citation patterns and may inform authors’ decisions about publication models when disseminating research findings.”

In the Discussion:

Addition to limitations: “Finally, because this is an observational bibliometric analysis, all findings represent associations rather than causal effects.”

Comment: There is no mention of equity implications of APCs for authors of low and middle income countries. It requires more explanation.

Response: We thank the reviewer for highlighting this important point and agree that the equity implications of APC-based models warrant more explicit discussion. We have expanded the Introduction to note how APCs may disproportionately disadvantage authors from low- and middle-income countries and unfunded institutions, and we have added a statement to the Discussion/Limitations explicitly acknowledging that APC-based OA models can exacerbate global inequities in who is able to publish OA.

Changes to Manuscript:

In the Introduction:

Original: “High APCs may deter researchers in low- and middle-income countries from pursuing OA routes despite publisher discounts. These costs may inadvertently reinforce the very inequities OA was intended to overcome.”

Revised: “High APCs may deter researchers in low- and middle-income countries and those at under-resourced institutions from pursuing OA routes, despite publisher discounts. These costs may inadvertently reinforce the very global inequities in who can publish and disseminate research that OA was intended to overcome.”

In the Discussion:

Original: N/A

Revised: “Sixth, APC-based OA models may exacerbate inequities in who can publish OA, particularly for authors from low- and middle-income countries or under-resourced institutions. Our analysis could not fully account for these constraints, so the observed citation advantages of OA should be interpreted in light of these access and affordability disparities.”

Attachment Submitted filename: Response to Reviewers .docx

# Peer review: Decision Letter 1

**DOI:** 10.1371/journal.pone.0360532.r003

**Authors:** Amitava Mukherjee

12 Jan 2026

PONE-D-25-46173R1

Is OA the way? A comparison of different open access classifications and their benefits in orthopaedic research

PLOS One?

Dear Dr. Cruickshank,

Thank you for submitting your manuscript to PLOS ONE. After careful consideration, we feel that it has merit but does not fully meet PLOS ONE’s publication criteria as it currently stands. Therefore, we invite you to submit a revised version of the manuscript that addresses the points raised during the review process.

Please submit your revised manuscript by Feb 26 2026 11:59PM. If you will need more time than this to complete your revisions, please reply to this message or contact the journal office at plosone@plos.org. When you're ready to submit your revision, log on to https://www.editorialmanager.com/pone/ and select the 'Submissions Needing Revision' folder to locate your manuscript file.

- A letter that responds to each point raised by the academic editor and reviewer(s). You should upload this letter as a separate file labeled 'Response to Reviewers'.
- A marked-up copy of your manuscript that highlights changes made to the original version. You should upload this as a separate file labeled 'Revised Manuscript with Track Changes'.
- An unmarked version of your revised paper without tracked changes. You should upload this as a separate file labeled 'Manuscript'.

If you would like to make changes to your financial disclosure, please include your updated statement in your cover letter. Guidelines for resubmitting your figure files are available below the reviewer comments at the end of this letter.

https://journals.plos.org/plosone/s/submission-guidelines#loc-laboratory-protocols. Additionally, PLOS ONE offers an option for publishing peer-reviewed Lab Protocol articles, which describe protocols hosted on protocols.io. Read more information on sharing protocols at https://plos.org/protocols?utm_medium=editorial-email&utm_source=authorletters&utm_campaign=protocols.

We look forward to receiving your revised manuscript.

Kind regards,

Amitava Mukherjee, ME, Ph.D.

Academic Editor

PLOS One

Journal Requirements:

1. If the reviewer comments include a recommendation to cite specific previously published works, please review and evaluate these publications to determine whether they are relevant and should be cited. There is no requirement to cite these works unless the editor has indicated otherwise.

2. Please review your reference list to ensure that it is complete and correct. If you have cited papers that have been retracted, please include the rationale for doing so in the manuscript text, or remove these references and replace them with relevant current references. Any changes to the reference list should be mentioned in the rebuttal letter that accompanies your revised manuscript. If you need to cite a retracted article, indicate the article’s retracted status in the References list and also include a citation and full reference for the retraction notice.

[Note: HTML markup is below. Please do not edit.]

Reviewers' comments:

Reviewer's Responses to Questions

**Comments to the Author**

Reviewer #1: (No Response)

Reviewer #2: All comments have been addressed

**********

2. Is the manuscript technically sound, and do the data support the conclusions?

Reviewer #1: Partly

Reviewer #2: Yes

**********

3. Has the statistical analysis been performed appropriately and rigorously?

Reviewer #1: Yes

Reviewer #2: Yes

**********

4. Have the authors made all data underlying the findings in their manuscript fully available?

The PLOS Data policy

Reviewer #1: No

Reviewer #2: Yes

**********

5. Is the manuscript presented in an intelligible fashion and written in standard English?

Reviewer #1: No

Reviewer #2: Yes

**********

Reviewer #1: Thank you for the revised submission. The manuscript has been improved, and several key points raised in the previous round appear to have been addressed. The statistical approach remains generally appropriate for the study's aims, including the count modeling framework and the diagnostics and robustness checks described. The overall direction of the findings is plausible given the dataset and design.

At the same time, I am not yet able to consider the manuscript entirely acceptable as presented because internal inconsistencies and presentation issues remain. The reporting of results should be reconciled across sections so that effect estimates and their interpretation are consistent between the Abstract, the main Results narrative, the Tables and Figures, and the Discussion. The manuscript still reads as if multiple model specifications, such as weighted versus unweighted analyses or primary versus sensitivity analyses, are discussed without a clearly stated hierarchy. Please make explicit which model constitutes the primary basis for inference, clearly label alternative specifications as sensitivity or robustness analyses, and align the narrative conclusions accordingly.

The handling and description of AIS and missingness also require more precise and internally consistent reporting. The revised text contains conflicting statements regarding the AIS source and temporal reference, as well as whether missing AIS values were imputed. Please ensure the manuscript and rebuttal are aligned and provide one definitive description of the AIS data used, how missing AIS values were handled, including whether any imputation was applied, and include a brief sensitivity check or an explicit robustness statement showing that the main conclusions do not hinge on the AIS handling choices.

A thorough language and formatting review is still needed before publication. The revised files contain visible word processing artifacts, such as “Formatted: …”, typographical errors, and at least one placeholder value in the Methods. Please remove any remaining artifacts, correct typographical and grammar issues, ensure that no placeholders remain in either the manuscript or the response to reviewers document, and confirm that the Methods and Results text is internally consistent and coherent throughout, as PLOS ONE does not copyedit accepted manuscripts.

Regarding data availability, the statement indicates that the underlying data are available via supporting information and or a public repository. Please verify that the actual datasets required to reproduce the analyses, not just scripts and a README, are accessible without restriction. If not, clearly specify any limitations and provide an appropriate publicly accessible alternative, such as anonymized datasets deposited in a stable repository with a versioned release or DOI. This is particularly important because the public repository documentation suggests the analysis relies on external input datasets. I have no additional concerns regarding dual publication, research, or publication ethics based on the materials provided. Once the reporting inconsistencies, AIS, and missing data description, presentation artifacts, and data access details are corrected, the manuscript should be suitable for publication.

Reviewer #2: All concerns have been addressed . Language check is required.Links of the datasets should be checked.

**********

what does this mean?). If published, this will include your full peer review and any attached files.

If you choose “no”, your identity will remain anonymous but your review may still be made public.

**Do you want your identity to be public for this peer review?**  For information about this choice, including consent withdrawal, please see our Privacy Policy

Reviewer #1: No

Reviewer #2: No

**********

[NOTE: If reviewer comments were submitted as an attachment file, they will be attached to this email and accessible via the submission site. Please log into your account, locate the manuscript record, and check for the action link "View Attachments". If this link does not appear, there are no attachment files.]

To ensure your figures meet our technical requirements, please review our figure guidelines: https://journals.plos.org/plosone/s/figures

You may also use PLOS’s free figure tool, NAAS, to help you prepare publication quality figures: https://journals.plos.org/plosone/s/figures#loc-tools-for-figure-preparation.

NAAS will assess whether your figures meet our technical requirements by comparing each figure against our figure specifications.

# Author comment: Author response to Decision Letter 2

**DOI:** 10.1371/journal.pone.0360532.r004

26 Feb 2026

February 26th, 2026

Manuscript ID: PONE-D-25-46173

Manuscript Title: Is OA the way? A comparison of different open access classifications and their benefits in orthopaedic research

Dear Reviewers and the editorial team at PLOS ONE,

Thank you so much for your review and thorough feedback of our original manuscript entitled “Is OA the way? A comparison of different open access classifications and their benefits in orthopaedic research” by Daniels et al. We appreciate the effort and hard work of your editorial team and reviewers. Please find attached in the new word document our detailed responses to each comment and our revised manuscript with all changes made and bolded for clarity.

Thank you for your support of our research initiatives. Please advise if you require any further information.

Sincerely,

Michelle Cruickshank, MD (candidate)

Corresponding author: cruicm5@mcmaster.ca

On behalf of co-authors:

William Daniels, MD (candidate)

Taylor Dejong, MD (candidate)

Michelle Ghert, MD, FRCSC

Reviewer #1: Thank you for the revised submission. The manuscript has been improved, and several key points raised in the previous round appear to have been addressed. The statistical approach remains generally appropriate for the study's aims, including the count modeling framework and the diagnostics and robustness checks described. The overall direction of the findings is plausible given the dataset and design.

Comment:

A thorough language and formatting review is still needed before publication. The revised files contain visible word processing artifacts, such as “Formatted: …”, typographical errors, and at least one placeholder value in the Methods. Please remove any remaining artifacts, correct typographical and grammar issues, ensure that no placeholders remain in either the manuscript or the response to reviewers document, and confirm that the Methods and Results text is internally consistent and coherent throughout, as PLOS ONE does not copyedit accepted manuscripts.

Response:

We thank you for highlighting this important point. We have gone through the manuscript and removed remaining artifacts and fixed the typographical errors previously present. Extensive changes have been made throughout the manuscript and can be seen in the tracked changes document.

Comment:

At the same time, I am not yet able to consider the manuscript entirely acceptable as presented because internal inconsistencies and presentation issues remain. The reporting of results should be reconciled across sections so that effect estimates and their interpretation are consistent between the Abstract, the main Results narrative, the Tables and Figures, and the Discussion. The manuscript still reads as if multiple model specifications, such as weighted versus unweighted analyses or primary versus sensitivity analyses, are discussed without a clearly stated hierarchy. Please make explicit which model constitutes the primary basis for inference, clearly label alternative specifications as sensitivity or robustness analyses, and align the narrative conclusions accordingly.

Response:

We thank the reviewer for this important clarification. We have revised the Statistical Analysis section to explicitly state that the entropy-balanced negative binomial generalized linear mixed model (GLMM) constitutes the primary basis for inference in this study. The revised manuscript now clearly labels this model as the primary analysis and distinguishes alternative specifications, including unweighted models and non-parametric comparisons, as sensitivity or descriptive analyses only.

We have also ensured that all inferential conclusions throughout the Abstract, Results, Tables, Figures, and Discussion are based exclusively on the weighted negative binomial GLMM. The hierarchy of analyses is now clearly defined in the Methods, and figure captions have been revised where necessary to reflect this distinction. We believe this revision resolves the ambiguity regarding model specification and strengthens the internal consistency of the manuscript.

Comment:

The handling and description of AIS and missingness also require more precise and internally consistent reporting. The revised text contains conflicting statements regarding the AIS source and temporal reference, as well as whether missing AIS values were imputed. Please ensure the manuscript and rebuttal are aligned and provide one definitive description of the AIS data used, how missing AIS values were handled, including whether any imputation was applied, and include a brief sensitivity check or an explicit robustness statement showing that the main conclusions do not hinge on the AIS handling choices.

Response:

Thank you for bringing the matter of AIS to our attention, we have incorrectly reported in previous versions of this manuscript that AIS were imputed. We can confidently say that AIS values were taken from SCImago for each journal and then joined to the dataframe to make calculations. This means no imputation or assumptions were taken.

Comment:

Regarding data availability, the statement indicates that the underlying data are available via supporting information and or a public repository. Please verify that the actual datasets required to reproduce the analyses, not just scripts and a README, are accessible without restriction. If not, clearly specify any limitations and provide an appropriate publicly accessible alternative, such as anonymized datasets deposited in a stable repository with a versioned release or DOI. This is particularly important because the public repository documentation suggests the analysis relies on external input datasets. I have no additional concerns regarding dual publication, research, or publication ethics based on the materials provided. Once the reporting inconsistencies, AIS, and missing data description, presentation artifacts, and data access details are corrected, the manuscript should be suitable for publication.

Response:

The data for AIS and for data for the main scripts calculations (along with the .rds that were generated) have been added to the github. The github README was also updated to take into account the new script layout. The new data objects are “AIS2023 … csv”, “min_data.csv”, and “final_weighted_model.rds”. The script required the removal of author names to personal identification purposes so the main.sh will bypass the “01_data_prep.R” script to execute the main analysis “02_analysis.R” script with the given data.

Comment:

Reviewer #2: All concerns have been addressed . Language check is required.Links of the datasets should be checked.

Response:

Thank you for your time you took to review our manuscript.

Attachment Submitted filename: Response to Reviewers (3).docx

# Peer review: Decision Letter 2

**DOI:** 10.1371/journal.pone.0360532.r005

**Authors:** Amitava Mukherjee

30 Mar 2026

PONE-D-25-46173R2

Is OA the way? A comparison of different open access classifications and their benefits in orthopaedic research

PLOS One?

Dear Dr. Cruickshank,

Thank you for submitting your manuscript to PLOS ONE. After careful consideration, we feel that it has merit but does not fully meet PLOS ONE’s publication criteria as it currently stands. Therefore, we invite you to submit a revised version of the manuscript that addresses the points raised during the review process.

Please submit your revised manuscript by May 14 2026 11:59PM. If you will need more time than this to complete your revisions, please reply to this message or contact the journal office at plosone@plos.org. When you're ready to submit your revision, log on to https://www.editorialmanager.com/pone/ and select the 'Submissions Needing Revision' folder to locate your manuscript file.

- A letter that responds to each point raised by the academic editor and reviewer(s). You should upload this letter as a separate file labeled 'Response to Reviewers'.
- A marked-up copy of your manuscript that highlights changes made to the original version. You should upload this as a separate file labeled 'Revised Manuscript with Track Changes'.
- An unmarked version of your revised paper without tracked changes. You should upload this as a separate file labeled 'Manuscript'.

If you would like to make changes to your financial disclosure, please include your updated statement in your cover letter. Guidelines for resubmitting your figure files are available below the reviewer comments at the end of this letter.

If applicable, we recommend that you deposit your laboratory protocols in protocols.io to enhance the reproducibility of your results. Protocols.io assigns your protocol its own identifier (DOI) so that it can be cited independently in the future. For instructions see: https://journals.plos.org/plosone/s/submission-guidelines#loc-laboratory-protocols. Additionally, PLOS ONE offers an option for publishing peer-reviewed Lab Protocol articles, which describe protocols hosted on protocols.io. Read more information on sharing protocols at https://plos.org/protocols?utm_medium=editorial-email&utm_source=authorletters&utm_campaign=protocols.

As the corresponding author, your ORCID iD is verified in the submission system and will appear in the published article. PLOS supports the use of ORCID, and we encourage all coauthors to register for an ORCID iD and use it as well. Please encourage your coauthors to verify their ORCID iD within the submission system before final acceptance, as unverified ORCID iDs will not appear in the published article. *Only*  the individual author can complete the verification step; PLOS staff *cannot*  verify ORCID iDs on behalf of authors.

We look forward to receiving your revised manuscript.

Kind regards,

Amitava Mukherjee, ME, Ph.D.

Academic Editor

PLOS One

Journal Requirements:

If the reviewer comments include a recommendation to cite specific previously published works, please review and evaluate these publications to determine whether they are relevant and should be cited. There is no requirement to cite these works unless the editor has indicated otherwise.

Please review your reference list to ensure that it is complete and correct. If you have cited papers that have been retracted, please include the rationale for doing so in the manuscript text, or remove these references and replace them with relevant current references. Any changes to the reference list should be mentioned in the rebuttal letter that accompanies your revised manuscript. If you need to cite a retracted article, indicate the article’s retracted status in the References list and also include a citation and full reference for the retraction notice.

[Note: HTML markup is below. Please do not edit.]

Reviewers' comments:

Reviewer's Responses to Questions

**Comments to the Author**

Reviewer #1: (No Response)

Reviewer #2: All comments have been addressed

**********

2. Is the manuscript technically sound, and do the data support the conclusions?

Reviewer #1: Partly

Reviewer #2: Yes

**********

3. Has the statistical analysis been performed appropriately and rigorously?

Reviewer #1: Yes

Reviewer #2: Yes

**********

4. Have the authors made all data underlying the findings in their manuscript fully available?

The PLOS Data policy

Reviewer #1: Yes

Reviewer #2: Yes

**********

5. Is the manuscript presented in an intelligible fashion and written in standard English?

Reviewer #1: No

Reviewer #2: Yes

**********

Reviewer #1: Thank you for the revised submission. The manuscript has improved, and several of the concerns raised in the previous round appear to have been addressed. In particular, the primary inferential model is now more clearly identified, the overall statistical framework is easier to follow, and the study remains relevant to ongoing discussions about open-access publishing in orthopaedic research. The analytical approach appears generally appropriate for the dataset and research question, and the main direction of the findings is plausible.

At the same time, the manuscript is not fully ready for acceptance in its current form, because several issues of internal consistency and presentation remain unresolved. My main concern is that the description of AIS is still contradictory across the manuscript and the rebuttal. In the Methods, AIS is first described in connection with JCR, whereas the article-level metadata section states that AIS values were obtained from the 2024 SJR. In the response to reviewers, the authors again describe AIS as coming from SCImago and state that no imputation was performed. These descriptions need to be reconciled so that the manuscript provides a single, definitive, and internally consistent account of the AIS source, timing, and handling.

A second concern is that the interpretation of the open-access categories remains only partially aligned across sections. The adjusted model results indicate that Bronze OA modestly outperformed Closed Access. In contrast, the abstract conclusion still states that Gold was followed by Green and Closed Access and that Bronze underperformed. Those statements are not fully compatible with the adjusted comparisons presented in the main Results. The manuscript would be strengthened by ensuring that the abstract, Results, Discussion, figure captions, and conclusion all reflect the same hierarchy of findings and do not mix adjusted and unadjusted interpretations.

The presentation of the temporal interaction also still needs tightening. The main Results section appropriately describes a crossover between Green and Gold OA around 2016 to 2017, with Green performing better in older cohorts and Gold in more recent cohorts. However, the wording around this pattern is not yet fully polished, and the relevant figure caption remains awkward and internally unclear. This should be revised so that the temporal interpretation is stated once, clearly and consistently, without ambiguity.

In addition, the manuscript still requires a careful language and formatting review before publication. There are multiple visible residual problems, including typographical errors, grammar issues, awkward or incomplete sentences, formatting artifacts, and malformed reporting in the post hoc comparison section. Because PLOS ONE does not copyedit accepted manuscripts, these issues should be corrected carefully at revision. I strongly encourage the authors to perform one final line-by-line review of the abstract, Methods, Results, figure captions, and discussion to ensure that the manuscript is not only statistically coherent but also clear and publication-ready in its presentation.

I appreciate that the data availability information appears to have been strengthened, and I do not have additional concerns regarding research ethics or publication ethics based on the materials provided. My remaining concerns are primarily about internal consistency, reporting precision, and language quality. Once those points are corrected, the manuscript will be in a much stronger position for acceptance.

Reviewer #2: • Bronze OA requires more clarity.

• There is no APC data to help in cost benefit analysis, please include this in recommendations for future research.

• The study has not established causality-this should be included in the limitations.

• The work has not clearly stated the distribution-Poisson/Negative Binomial .It requires more explanation.

• IRRs require more explanation.

• Correlations between predictors have not been made clear.

• More explanation of statistical results is required.

• Figures require more explanatory captions.

**********

what does this mean?). If published, this will include your full peer review and any attached files.

If you choose “no”, your identity will remain anonymous but your review may still be made public.

**Do you want your identity to be public for this peer review?**  For information about this choice, including consent withdrawal, please see our Privacy Policy

Reviewer #1: No

Reviewer #2: No

**********

[NOTE: If reviewer comments were submitted as an attachment file, they will be attached to this email and accessible via the submission site. Please log into your account, locate the manuscript record, and check for the action link "View Attachments". If this link does not appear, there are no attachment files.]

To ensure your figures meet our technical requirements, please review our figure guidelines: https://journals.plos.org/plosone/s/figures

You may also use PLOS’s free figure tool, NAAS, to help you prepare publication quality figures: https://journals.plos.org/plosone/s/figures#loc-tools-for-figure-preparation.

NAAS will assess whether your figures meet our technical requirements by comparing each figure against our figure specifications.

# Author comment: Author response to Decision Letter 3

**DOI:** 10.1371/journal.pone.0360532.r006

12 Jun 2026

May 7th, 2026

Manuscript ID: PONE-D-25-46173

Manuscript Title: Is OA the way? A comparison of different open access classifications and their benefits in orthopaedic research

Dear Editor and Reviewers at PLOS ONE,

We sincerely thank the reviewers for their thoughtful and constructive feedback on our manuscript, “Is OA the way? A comparison of different open access classifications and their benefits in orthopaedic research”. We appreciate the opportunity to revise our work. In response to the reviewers’ comments, we have clarified the source and handling of Article Influence Score, improved consistency across the Abstract, Results, Discussion, figure captions, and Conclusion, expanded the interpretation of Bronze OA, strengthened the statistical methods and reporting, and added additional limitations regarding APC data and the observational nature of the study. We have also performed a line-by-line review to improve clarity, grammar, formatting, and consistency throughout the manuscript.

All changes have been made in the revised manuscript using tracked changes. Our point-by-point responses are provided below.

Sincerely,

Michelle Cruickshank, MD (candidate)

Corresponding author: cruicm5@mcmaster.ca

On behalf of co-authors:

William Daniels, MD (candidate), Taylor Dejong, MD (candidate), Michelle Ghert, MD, FRCSC

Reviewer #1:

Comment:

My main concern is that the description of AIS is still contradictory across the manuscript and the rebuttal. In the Methods, AIS is first described in connection with JCR, whereas the article-level metadata section states that AIS values were obtained from the 2024 SJR. In the response to reviewers, the authors again describe AIS as coming from SCImago and state that no imputation was performed. These descriptions need to be reconciled so that the manuscript provides a single, definitive, and internally consistent account of the AIS source, timing, and handling.

Response:

Thank you for identifying this important inconsistency. We agree that the prior wording created confusion regarding the source and handling of AIS. We have revised the manuscript to provide a single, consistent description throughout. AIS is now consistently described as a journal-level metric obtained from the 2024 SCImago journal reports and matched to articles by journal title. We also clarified that AIS values were available for all included journals and that no imputation or substitution was performed.

Revision made:

We revised the Journal Identification and Individual Article Metadata Extraction sections to consistently state the source, timing, and handling of AIS. We also reviewed the manuscript to ensure that AIS is no longer described inconsistently across sections.

Comment:

A second concern is that the interpretation of the open-access categories remains only partially aligned across sections. The adjusted model results indicate that Bronze OA modestly outperformed Closed Access. In contrast, the abstract conclusion still states that Gold was followed by Green and Closed Access and that Bronze underperformed. Those statements are not fully compatible with the adjusted comparisons presented in the main Results. The manuscript would be strengthened by ensuring that the abstract, Results, Discussion, figure captions, and conclusion all reflect the same hierarchy of findings and do not mix adjusted and unadjusted interpretations.

Response:

We thank you for also highlighting this important inconsistency in our work. The Abstract, Results, and Conclusion has been updated to reflect this more clearly. At first glance, the boxplot (Fig 1) indicates that there is a clear statistically significant difference between all open-access categories from ascending order of (Bronze -> Closed Access -> Green -> Gold). Upon further analysis using the entropy-balanced negative binomial GLMM, which incorporates publication year and document type as covariates (Fig 2), the temporal dynamics between open access categories become clearer. Specifically, Gold OA demonstrated higher predicted citation counts than Green OA in more recent publication cohorts (e.g., 2019), consistent with the advantage of immediate accessibility. However, in earlier cohorts with longer citation windows (e.g., 2014), this relationship reversed, with Green OA surpassing Gold OA in predicted citation counts. This crossover pattern reflects the significant interaction between OA type and publication year observed in the primary model.

The relationship between Bronze and Closed Access articles also exhibited a temporal pattern. In more recent cohorts, Bronze OA showed modestly higher predicted citation counts than Closed Access. However, this advantage diminished over time, and in earlier cohorts the two categories demonstrated comparable predicted citation levels across document types. These findings underscore that citation performance varies not only by access type but also by citation window length and temporal accumulation dynamics.

To investigate the apparent citation advantage of Closed Access over Bronze OA in the unadjusted boxplot, we examined document-type composition across access categories. Bronze OA contained a higher proportion of lower-cited document types (e.g., editorial materials) and fewer standard articles; after adjusting for document type in the primary model, the difference between Bronze and Closed Access was no longer statistically significant.

Revision made:

We revised the Abstract, Results, Discussion, Figure 1 caption, and Conclusion to distinguish clearly between unadjusted and adjusted findings. We also expanded Figure 1 and the accompanying Results text to describe document-type composition across OA categories and the articles-only analysis.

Comment:

The presentation of the temporal interaction also still needs tightening. The main Results section appropriately describes a crossover between Green and Gold OA around 2016 to 2017, with Green performing better in older cohorts and Gold in more recent cohorts. However, the wording around this pattern is not yet fully polished, and the relevant figure caption remains awkward and internally unclear. This should be revised so that the temporal interpretation is stated once, clearly and consistently, without ambiguity.

Response:

Thank you for this helpful suggestion. We agree that the temporal interaction required clearer and more consistent wording. We revised the Results, Discussion, Figure 2 caption, and Conclusion to describe the crossover between Green and Gold OA more clearly. The revised manuscript now states that Green OA had higher predicted citation counts in older publication cohorts, whereas Gold OA had higher predicted citation counts in more recent cohorts, with a crossover around 2016–2017.

We also clarified that this analysis compares cumulative citation counts across publication-year cohorts rather than following individual articles longitudinally. Therefore, the temporal interaction should be interpreted as an association across cohorts rather than direct evidence of within-article citation accumulation over time.

Revision made:

We revised the temporal interaction section of the Results, the Figure 2 caption, the Discussion, and the Conclusion to ensure that the Green-versus-Gold crossover is described consistently and without ambiguity.

Comment:

In addition, the manuscript still requires a careful language and formatting review before publication. There are multiple visible residual problems, including typographical errors, grammar issues, awkward or incomplete sentences, formatting artifacts, and malformed reporting in the post hoc comparison section. Because PLOS ONE does not copyedit accepted manuscripts, these issues should be corrected carefully at revision. I strongly encourage the authors to perform one final line-by-line review of the abstract, Methods, Results, figure captions, and discussion to ensure that the manuscript is not only statistically coherent but also clear and publication-ready in its presentation.

Response:

Thank you for this important comment. We agree that the manuscript required additional language and formatting refinement prior to publication. We performed a detailed line-by-line review of the Abstract, Methods, Results, figure captions, Discussion, and Conclusion. We corrected typographical errors, grammar issues, formatting artifacts, and incomplete or awkward sentences. We also revised the post hoc comparison section to improve clarity and ensure that effect estimates, confidence intervals, and p-values are reported consistently.

Revision made:

We performed a manuscript-wide language and formatting review and revised the Abstract, Methods, Results, figure captions, Discussion, Conclusion, and post hoc comparison section for clarity, consistency, and publication readiness.

Reviewer #2

Comment:

• Bronze OA requires more clarity.

Response:

Thank you for this helpful comment. We agree that the Bronze OA category required additional clarification. We revised the Methods to define Bronze OA more clearly as free-to-read articles available on the publisher’s website without a clear open license. We also expanded the Results and Discussion to explain that Bronze OA is heterogeneous and may include articles made freely available temporarily or at the publisher’s discretion.

In addition, we added new analyses showing that Bronze OA was disproportionately composed of lower-cited publication types, including editorial materials, letters, and corrections. This helped explain why Bronze OA had the lowest citation counts in unadjusted analyses despite modestly outperforming Closed Access in the adjusted model.

Revision made:

We clarified the definition of Bronze OA in the Methods and Table 1, expanded the Results to describe Bronze OA document-type composition, and revised the Discussion and Conclusion to distinguish the unadjusted and adjusted Bronze OA findings.

• There is no APC data to help in cost benefit analysis, please include this in recommendations for future research.

Response:

Thank you for this point. We agree that the lack of article-level APC data limits our ability to assess cost-effectiveness across OA models. We have this point in both the Limitations and Future Directions sections. We state that future research should incorporate article-level APC, licensing, and embargo data to enable a more comprehensive cost-benefit analysis of OA publishing in orthopaedics.

Revision made:

We added the absence of article-level APC data as a limitation and revised the Future Directions paragraph to recommend incorporation of APC data in future studies.

• The study has not established causality-this should be included in the limitations.

Response:

Thank you for this important comment. We agree. Because this study is observational and bibliometric in design, it cannot establish causal relationships between OA status and citation counts. We have added this explicitly to the Limitations section and revised language throughout the manuscript to avoid causal interpretation.

Revision made:

We added a final sentence to the Limitations section stating that, because this was an observational bibliometric analysis, all findings should be interpreted as associations rather than causal effects. We also revised causal wording throughout the manuscript where appropriate.

• The work has not clearly stated the distribution-Poisson/Negative Binomial .It requires more explanation.

Response:

Thank you for this helpful comment. We agree that the model distribution and rationale required clearer explanation. We expanded the Statistical Analysis section to explain that citation counts are overdispersed count data. Although a Poisson model was initially considered, likelihood ratio testing showed substantial overdispersion and demonstrated that the negative binomial model provided a substantially better fit. We also clarified that a zero-inflated negative binomial model was assessed but did not improve model fit, indicating that the standard negative binomial model adequately captured the observed zero-citation articles.

Revision made:

We expanded the Statistical Analysis section to include model justification, comparison of Poisson and negative binomial models, assessment of zero inflation, and rationale for the final negative binomial GLMM.

• IRRs require more explanation.

Response:

Thank you for this suggestion. We revised the Statistical Analysis and Results sections to clarify that model coefficients were exponentiated and reported as incidence rate ratios. We also added explanatory language indicating that IRRs represent multiplicative differences in expected citation rates between groups, after adjustment for model covariates.

Revision made:

We clarified the interpretation of IRRs in the Statistical Analysis section and revised the Results to report IRRs in a more reader-friendly manner.

• Correlations between predictors have not been made clear.

Response:

We thank you for raising this point. We clarified the model structure and interpretation of random effects, including the correlation between journal-specific random intercepts and publication-year slopes. We now report that the strong negative correlation between intercepts and slopes indicates that journals with higher baseline citation rates showed attenuated citation growth over time. We also clarified that continuous predictors were standardized prior to analysis to improve model convergence and facilitate interpretation.

Revision made:

We revised the Model Structure and Advanced Model Results sections to clarify the correlation between random intercepts and slopes and to improve explanation of standardized predictors.

• More explanation of statistical results is required.

Response:

Thank you for this comment. We expanded the Statistical Analysis and Results sections to improve the explanation of the model and its findings. Specifically, we added clearer descriptions of entropy balancing, model fit, overdispersion testing, zero-inflation assessment, Type II Wald chi-square tests, post hoc pairwise comparisons, and interpretation of the OA type x publication year interaction.

Revision made:

We revised the Statistical Analysis and Results sections to provide additional explanation of model selection, model diagnostics, covariate balance, IRRs, post hoc comparisons, and temporal interaction findings.

• Figures require more explanatory captions.

Response:

We thank you for this suggestion. We revised the figure captions to make them more self-contained and explanatory. Figure 1 now describes the unadjusted citation distributions, document-type composition across OA categories, standardized residuals from the chi-square analysis, and the articles-only analysis. Figure 2 now more clearly describes predicted citation counts by publication year and OA category, including the temporal crossover between Gold and Green OA. Figure 3 now describes the association between author count and predicted citation counts across OA categories.

Revision made:

We expanded and clarified the captions for Figures 1-3 to improve interpretability and to better align each figure with the corresponding Results text.

Attachment Submitted filename: OA Study - Response to Reviewers CLEAN.docx

# Peer review: Decision Letter 3

**DOI:** 10.1371/journal.pone.0360532.r007

**Authors:** Amitava Mukherjee

8 Sep 2026

PONE-D-25-46173R3

Is OA the way? A comparison of different open access classifications and their benefits in orthopaedic research

PLOS One

Dear Dr. Cruickshank,

Thank you for submitting your manuscript to PLOS One. After careful consideration, we feel that it has merit but does not fully meet PLOS One’s publication criteria as it currently stands. Therefore, we invite you to submit a revised version of the manuscript that addresses the points raised during the review process.

Please submit your revised manuscript by Oct 23 2026 11:59PM. If you will need more time than this to complete your revisions, please reply to this message or contact the journal office at plosone@plos.org. When you're ready to submit your revision, log on to https://www.editorialmanager.com/pone/ and select the 'Submissions Needing Revision' folder to locate your manuscript file.

Please include the following items when submitting your revised manuscript:

- A letter that responds to each point raised by the academic editor and reviewer(s). You should upload this letter as a separate file labeled 'Response to Reviewers'.
- A marked-up copy of your manuscript that highlights changes made to the original version. You should upload this as a separate file labeled 'Revised Manuscript with Track Changes'.
- An unmarked version of your revised paper without tracked changes. You should upload this as a separate file labeled 'Manuscript'.

If you would like to make changes to your financial disclosure, please include your updated statement in your cover letter. Guidelines for resubmitting your figure files are available below the reviewer comments at the end of this letter.

If applicable, we recommend that you deposit your laboratory protocols in protocols.io to enhance the reproducibility of your results. Protocols.io assigns your protocol its own identifier (DOI) so that it can be cited independently in the future. For instructions see: https://journals.plos.org/plosone/s/submission-guidelines#loc-laboratory-protocols. Additionally, PLOS One offers an option for publishing peer-reviewed Lab Protocol articles, which describe protocols hosted on protocols.io. Read more information on sharing protocols at https://plos.org/protocols?utm_medium=editorial-email&utm_source=authorletters&utm_campaign=protocols.

As the corresponding author, your ORCID iD is verified in the submission system and will appear in the published article. PLOS supports the use of ORCID, and we encourage all coauthors to register for an ORCID iD and use it as well. Please encourage your coauthors to verify their ORCID iD within the submission system before final acceptance, as unverified ORCID iDs will not appear in the published article. *Only*  the individual author can complete the verification step; PLOS staff *cannot*  verify ORCID iDs on behalf of authors.

We look forward to receiving your revised manuscript.

Kind regards,

Amitava Mukherjee, ME, Ph.D.

Academic Editor

PLOS One

**Journal Requirements:**

1. If the reviewer comments include a recommendation to cite specific previously published works, please review and evaluate these publications to determine whether they are relevant and should be cited. There is no requirement to cite these works unless the editor has indicated otherwise.

2. Please review your reference list to ensure that it is complete and correct. If you have cited papers that have been retracted, please include the rationale for doing so in the manuscript text, or remove these references and replace them with relevant current references. Any changes to the reference list should be mentioned in the rebuttal letter that accompanies your revised manuscript. If you need to cite a retracted article, indicate the article’s retracted status in the References list and also include a citation and full reference for the retraction notice.

[Note: HTML markup is below. Please do not edit.]

Reviewers' comments:

Reviewer's Responses to Questions

**Comments to the Author**

1. If the authors have adequately addressed your comments raised in a previous round of review and you feel that this manuscript is now acceptable for publication, you may indicate that here to bypass the “Comments to the Author” section, enter your conflict of interest statement in the “Confidential to Editor” section, and submit your "Accept" recommendation.

Reviewer #2: All comments have been addressed

**********

2. Is the manuscript technically sound, and do the data support the conclusions?

The manuscript must describe a technically sound piece of scientific research with data that supports the conclusions. Experiments must have been conducted rigorously, with appropriate controls, replication, and sample sizes. The conclusions must be drawn appropriately based on the data presented.

Reviewer #2: Yes

**********

3. Has the statistical analysis been performed appropriately and rigorously?

Reviewer #2: Yes

**********

4. Have the authors made all data underlying the findings in their manuscript fully available?

The PLOS Data policy requires authors to make all data underlying the findings described in their manuscript fully available without restriction, with rare exception (please refer to the Data Availability Statement in the manuscript PDF file). The data should be provided as part of the manuscript or its supporting information, or deposited to a public repository. For example, in addition to summary statistics, the data points behind means, medians and variance measures should be available. If there are restrictions on publicly sharing data—e.g. participant privacy or use of data from a third party—those must be specified.

Reviewer #2: Yes

**********

5. Is the manuscript presented in an intelligible fashion and written in standard English?

PLOS ONE does not copyedit accepted manuscripts, so the language in submitted articles must be clear, correct, and unambiguous. Any typographical or grammatical errors should be corrected at revision, so please note any specific errors here.

Reviewer #2: Yes

**********

6. Review Comments to the Author

Please use the space provided to explain your answers to the questions above. You may also include additional comments for the author, including concerns about dual publication, research ethics, or publication ethics. (Please upload your review as an attachment if it exceeds 20,000 characters)

Reviewer #2: 1. Please provide a reproducible journal selection flowchart showing all candidate journals, and reasons for exclusion, and an explicit rationale for the 2 mainly added journals,

2. The gold OA in the dataset consists exclusively of hybrid OA articles, because journals with >95% OA were excluded.

In abstract, results figures discussion and conclusion, please use “Gold/hybrid OA or hybrid Gold OA” and write in the abstract that fully OA journals were not represented. This will avoid over generalization.

3. Please frame the research question more narrowly as comparing OA routes within predominantly subscription/hybrid high-impact journals, rather than comparing OA models in general.

4. Please report weight distribution, maximum weights, and an overlap in the supplementary material. A sensitivity analysis using trimmed/truncated weights would also be useful.

5. If possible, separate Green subtypes and distinguish bronze access duration.

If it is not possible, mention that the analysis evaluates broad database derived. OA classification rather than homogeneous publishing strategies.

6. Consider citation rate per year, citations accumulated within a standardized window, were data permit or write that current analysis includes cumulative citation counts with unequal follow up periods.

7. Please use year specific journal influence metrics corresponding to each publication year.

8. Title should be changed. Please mention “citation impact/visibility” rather than general benefits or include additional impact metrics.

9. Please use the same words throughout-instead of advantage outperform, benefit or impact.

10. Please distinguish between three layers-unadjusted descriptive findings primary adjusted findings and sensitivity.

**********

7. PLOS authors have the option to publish the peer review history of their article (what does this mean?). If published, this will include your full peer review and any attached files.

If you choose “no”, your identity will remain anonymous but your review may still be made public.

**Do you want your identity to be public for this peer review?**  For information about this choice, including consent withdrawal, please see our Privacy Policy.

Reviewer #2: No

**********

[NOTE: If reviewer comments were submitted as an attachment file, they will be attached to this email and accessible via the submission site. Please log into your account, locate the manuscript record, and check for the action link "View Attachments". If this link does not appear, there are no attachment files.]

To ensure your figures meet our technical requirements, please review our figure guidelines: https://journals.plos.org/plosone/s/figures

You may also use PLOS’s free figure tool, NAAS, to help you prepare publication quality figures: https://journals.plos.org/plosone/s/figures#loc-tools-for-figure-preparation.

NAAS will assess whether your figures meet our technical requirements by comparing each figure against our figure specifications.

# Author comment: Author response to Decision Letter 4

**DOI:** 10.1371/journal.pone.0360532.r008

26 Sep 2026

Response to Reviewers: PONE-D-25-46173R3 (revised)

Manuscript: PONE-D-25-46173R3

Revised title: Open access route and citation impact in orthopaedic research: a comparison of Gold/hybrid, Green, and Bronze open access within ten high-impact subscription-based journals

Journal: PLOS ONE

Dear Dr. Mukherjee,

Thank you for reviewing out manuscript, we appreciate your time with this paper. We have addressed all ten points.

A short summary of what was changed:

The paper is now explicitly about comparing OA routes within predominantly subscription/hybrid journals, not about OA publishing in general. This runs through the title, abstract, introduction, discussion, and conclusion.

The Gold category is called Gold/hybrid OA everywhere now, including every remaining in-text mention in the Methods, Results, and Discussion, and the abstract says that fully OA journals were not represented.

We added a journal selection flow diagram and a screening log.

We added the weighting diagnostics that were missing, weight distribution, maximum weights, effective sample sizes, overlap, plus a trimmed-weights sensitivity analysis.

We split Green OA into its three subtypes and analysed them separately.

We added a citations-per-year analysis, and said plainly that our main outcome is a cumulative count with unequal follow-up.

We added a journal-by-year random effect to deal with year-specific journal influence, and now state why year-specific journal metrics could not be substituted directly.

We picked one set of words and removed the last evaluative terms ("advantage", "performance", "deficit", "benefit").

The Results are now split into three labelled layers: unadjusted, primary adjusted, and sensitivity.

We corrected several internal inconsistencies uncovered during this revision (see "A few other things we changed").

Three supplementary figures and four supplementary tables were added. We kept the supplement compact and grouped related results into panels within a single file, rather than splitting every result into its own item. None of our conclusions changed. Section names below refer to the revised manuscript. Everything is visible in the tracked-changes version.

Academic Editor

Requirement 1: citing specific published works. Reviewer #2 did not ask us to cite anything in particular, so we have not added citations on that basis.

Requirement 2: reference list and retracted articles. We went back through the reference list. It is complete and correctly formatted, none of the 24 references is retracted, and we did not add, remove, or replace any references in this revision.

Reviewer #2

1. Please provide a reproducible journal selection flowchart showing all candidate journals, and reasons for exclusion, and an explicit rationale for the 2 mainly added journals.

Thank you for your comment.

We added S1 Fig, a flow diagram covering the whole selection process: journals screened, how many were excluded at each step and why, then the article-level flow. We also added S1 Table, a screening log with one row per journal assessed, giving the SJR, JCR quartile, AIS, percentage OA, whether it was included, and why. Both are generated by a script that ships with the analysis code, so the numbers come straight from the data rather than being typed in by hand.

The Methods previously said two journals were added on senior author recommendation: the Journal of Bone and Joint Surgery (JBJS-American) and Clinical Orthopaedics and Related Research (CORR). When we checked that against the actual dataset, it was wrong. Only one journal was added: the Journal of Bone and Joint Surgery - American. CORR is not in the dataset at all and contributes no articles to any analysis. We have corrected the Methods and we apologize for the error.

JBJS-American was suggested by the senior author because of its standing in the specialty, and it also met all three inclusion criteria on its own. So it was not an exception to our criteria, and we now say so. It appears as its own box in S1 Fig.

Where: Methods, "Journal identification"; S1 Fig; S1 Table.

2. …please use "Gold/hybrid OA or hybrid Gold OA" … and write in the abstract that fully OA journals were not represented.

Thank you for pointing this out. The category is Gold/hybrid OA everywhere now including the title, abstract, results, figures, tables, discussion, and conclusion.

The abstract now says it in the third sentence: "Journals in which more than 95% of articles were OA were excluded by design; fully OA journals were therefore not represented, and every Gold OA article in this corpus was published as hybrid OA within a subscription journal (hereafter Gold/hybrid OA)." And the last sentence returns to it: "Within subscription-based orthopaedic journals, OA routes are not interchangeable; these findings do not extend to fully OA journals." The same point is made in the methods, the limitations, and conclusion.

Where: throughout.

3. Please frame the research question more narrowly as comparing OA routes within predominantly subscription/hybrid high-impact journals.

Thank you for bringing out attention to this point. We have made the changes accordingly.

The last paragraph of the Introduction now states: "among articles published in high-impact orthopaedic journals that operate on a predominantly subscription or hybrid basis, is the OA route by which an article becomes freely readable associated with its citation rate?" It then says what the study is not: "The study therefore does not compare fully OA journals with subscription journals, and it does not evaluate OA publishing models in general."

The title carries the same restriction. The conclusion opens with "Within ten high-impact orthopaedic journals that publish predominantly on a subscription or hybrid basis…" and closes by saying these findings do not speak to the fully-OA-versus-subscription question. The first limitations also report the same statement.

Where: Title; Abstract; Introduction, final paragraph; Discussion; Limitations; Conclusion.

4. Please report weight distribution, maximum weights, and an overlap … A sensitivity analysis using trimmed/truncated weights would also be useful.

We have added weight diagnostics, a common-support assessment, and truncated-weight sensitivity analyses.

Weights (S2 Table panel A; S2 Fig A–B). Entropy balancing sets the mean weight to 1.00 in every group. Maximum weights were 11.06 (Gold/hybrid OA), 5.76 (Green OA), 4.02 (Bronze OA), and 3.71 (Closed Access); variability was greatest in Gold/hybrid OA (CV 1.17) and Bronze OA (1.12) and least in Closed Access (0.18). The heaviest 1% of articles carried 3.7% of total weight overall and 9.0% within Gold/hybrid OA. Effective sample size was 19,546 of 24,115 overall (81.1%); by group, 386 of 911 (42.4%) for Gold/hybrid OA, 925 of 2,093 (44.2%) for Bronze OA, 1,431 of 2,187 (65.4%) for Green OA, and 18,343 of 18,924 (96.9%) for Closed Access.

Overlap (S2 Table panel B; S2 Fig C). We assessed common support using generalized propensity scores from a multinomial logistic regression on the covariates used for weighting. Support was good but not complete: 23,011 of 24,115 articles (95.4%) fell within the region where all four categories were represented. Of the 1,104 (4.6%) outside, most (885) lay outside on the Gold/hybrid dimension, 880 of them with a predicted probability of Gold/hybrid OA below the minimum observed within that group. These were low-propensity tails rather than broad separation between groups.

Trimming (S2 Table panel C). We refit the primary model with weights truncated at the 99th, 97.5th, and 95th percentiles and at the 1st/99th. Truncation at the 99th altered 239 weights (1.0%) and reduced the maximum from 11.06 to 2.99. Balance was exact before truncation and remained acceptable at the 99th (largest absolute SMD 0.055) and 97.5th (0.086); at the 95th, two covariates exceeded 0.1 (largest 0.158), so the most aggressive truncation reduces weight variability at the cost of reintroducing imbalance.

Gold/hybrid OA and Green OA versus Closed Access were stable: 1.41 → 1.44, 1.44, 1.44 and 1.35 → 1.34, 1.31, 1.28. The Gold–Green contrast narrowed progressively (p = 0.689, 0.210, 0.065, 0.019), reaching significance only under the 95th-percentile specification, the one with compromised balance. The two-tailed 1st / 99th specification matched the 99th throughout.

Bronze OA versus Closed Access was more sensitive, moving from 1.09 (p = 0.003) to 1.05 (p = 0.269) at the 95th percentile. We report this openly and have removed the directional Bronze claim from the Discussion and Conclusion (see point 10).

Where: Methods, "Sensitivity and descriptive analyses"; Results, "Layer 3," paragraphs 1–2; S2 Table; S2 Fig.

5. If possible, separate Green subtypes and distinguish bronze access duration. If not, mention that the analysis evaluates broad database-derived OA classification rather than homogeneous publishing strategies.

Thank you for bringing up this point. We were able to do the Green subtypes.

Green subtypes (S4 Table). The Green-submitted, Green-accepted, and Green-published flags are still in the raw Web of Science export, so we pulled them back out, re-ran the entropy balancing with a six-level treatment variable, and refit. Green-submitted was 555 articles (2.3%), Green-accepted 823 (3.4%), Green-published 809 (3.4%).

All three had higher citation rates than Closed Access (1.58, 1.26, and 1.17; all p < 0.001), but they were not the same as each other. Green-submitted was higher than Green-accepted (1.25, 95% CI 1.07–1.46) and Green-published (1.35, 95% CI 1.16–1.58), while Green-accepted and Green-published did not differ (p = 0.544). Gold/hybrid OA was higher than Green-published (1.22, 95% CI 1.07–1.39) but did not differ from Green-submitted (p = 0.337) or Green-accepted (p = 0.115).

The ordering is consistent with the embargo account in our Discussion, though we note the subtypes are assigned by most-final version, so Green-published records may also have had earlier repository versions; the contrast is therefore between deposit patterns rather than strictly between access dates. Raw means run in the opposite order, reflecting the higher author counts and journal influence of Green-published articles, which the weighting adjusts for. Collapsing the three subtypes reproduced the original estimates to within ~3%.

Bronze duration. Unfortunately, it was not possible with our data, and we now say this plainly in both the Methods and the limitations. The Web of Science OA field is genuinely a one point snapshot from the moment we pulled the data, it does not record how long anything has been free to read. So Bronze OA here means "free to read when we looked", not "durably free", and we could not subdivide it by how long access lasted.

Database-derived labels. We took the reviewer's wording directly. The Methods now describe these as "database-derived access labels rather than homogeneous publishing strategies", and the limitations repeat it with numbers. The flags are not mutually exclusive, and our precedence rule (hybrid > Green > Bronze > none, now spelled out in the Methods) puts each article in the most privileged bucket it qualifies for. Concretely: 798 of the 911 Gold/hybrid OA articles (87.6%) also carry a Green flag, and 362 of the 2,187 Green OA articles (16.6%) also carry a Bronze flag. Reporting those overlaps seemed like the most honest way to make the reviewer's point.

Where: Methods, "Individual article metadata extraction"; Results, "Layer 3", paragraph 3; Discussion; Limitations; S4 Table.

6. Consider citation rate per year … or write that the current analysis includes cumulative citation counts with unequal follow-up periods.

Thank you for bringing this to our attention. We implemented the first suggestion, and for the second we did what the reviewer suggested and stated the limitation.

Citations per year (S5 Table panels C-D). Mean citations per year of follow-up were 2.31 for Bronze OA, 4.37 for Closed Access, 6.99 for Green OA, and 9.53 for Gold/hybrid OA — same ordering as the cumulative counts. We also refit the primary model with an offset for log(years since publication), which turns the outcome into a per-year rate rather than a running total. Almost nothing moved: Gold/hybrid OA versus Closed Access 1.41, Green OA versus Closed Access 1.36, Gold/hybrid OA versus Green OA 1.04 (p = 0.689). S5 Table also gives the follow-up window for each cohort.

Standardised window. We are unable to build one. The Web of Science export gives a single cumulative citation total per article, not citations year by year, so there is no way to reconstruct something like "citations within three years of publication". We say this in the Methods and again in the limitations, in the terms the reviewer suggested: the counts are cumulative totals at the extraction date, and follow-up ran from roughly five years for the 2019 cohort to roughly ten years for the 2014 cohort. Worth noting that publication year is in the model, and the OA-by-year interaction is one of the paper's actual findings, so the uneven follow-up is modelled rather than ignored.

Where: Methods, "Individual article metadata extraction" and "Sensitivity and descriptive analyses"; Results, "Layer 3", paragraph 4; Limitations; S5 Table.

7. Please use year-specific journal influence metrics corresponding to each publication year.

Thank you for highlighting this point. We were unable to obtain year-specific Article Influence Scores for all ten journals across 2014-2019, and we now state this explicitly in the Methods to explain why we took a different route rather than year-matching a single index. Rather than swap in a partial or approximate set, we used an approach that we think answers the underlying concern better.

Journal-by-year random intercept (S5 Table panel E). We added a random intercept for each journal-year cell. Any year-specific journal metric, AIS, impact factor, SJR, whichever is constant within a journal-year cell by definition, so this term soaks all of them up at once, along with anything else about a journal that changes from year to year (editorial turnover, indexing changes, a themed volume). It is a broader adjustment than plugging in a year-specific value for one index.

It improved fit (AIC 202,564 versus 202,773). The journal-by-year SD was 0.118 across 60 cells, smaller than the between-journal SD of 0.160, which suggests year-to-year movement in journal influence is modest next to the differences between journals. The OA estimates barely moved: Gold/hybrid OA versus Closed Access 1.40, Green OA versus Closed Access 1.31, Gold/hybrid OA versus Green OA 1.07 (p = 0.279).

It is also worth saying that the primary model already had a random slope for publication year by journal, so journal-specific citation trends over time were in there from the start.

We added the single-value AIS to the limitations, along with this result, so a reader sees both the shortcoming and the evidence that it does not drive the findings.

Where: Methods, "Individual article metadata extraction" and "Sensitivity and descriptive analyses"; Results, "Layer 3", paragraph 4; Limitations; S5 Table.

8. Title should be changed. Please mention "citation impact/visibility" rather than general benefits, or include additional impact metrics.

Thank you for bringing up this point. We have changed it from:

Is OA the way? A comparison of different open access classifications and their benefits in orthopaedic research

It is now:

Open access route and citation impact in orthopaedic research: a comparison of Gold/hybrid, Green, and Bronze open access within ten high-impact subscription-based journals

The new title describes the outcome instead of claiming a benefit, names the categories, and carries the subscription-journal restriction from point 3. We also cut the "Is OA the way?" hook and the "OA pays" line that opened the conclusion to prevent over-generalisation.

Where: Title

9. Please use the same words throughout - instead of advantage, outperform, benefit or impact.

Thank you for highlighting this point. We picked one vocabulary and said so in the Methods so the reader knows what we mean.

The new "Analytic framework" section states: "Throughout, 'citation rate' denotes the model-estimated expected number of citations for an article and 'citation count' denotes observed totals; differences between OA categories are reported as rate ratios. Evaluative terms such as 'advantage', 'benefit' and 'outperform' are avoided so that a single vocabulary is used throughout."

We made changes throughout the manuscript: "Citation advantage", "citation benefit", "citation performance", "outperformed", "underperformed", "superior citation performance", "the advantage conferred by", "greater citation impact", "apparent Bronze OA deficit", and "OA pays" are all gone, replaced with "had a higher/lower citation rate than" or "had a citation rate x times that of". In this revision we specifically corrected the Discussion opening ("citation advantage" → "higher citation rate"; "citation performance" → "citation rate") and the Layer 1 subheading ("deficit" → "difference"). "Citation impact" survives in the title only, as the general idea the reviewer asked us to name in point 8; inside the paper it is "citation rate" throughout. We fixed the headings to match ("Open access effects by author count" became "Citation rate by author count"; "Effects of collaboration, impact, and document type" became "Author count, journal influence, and document type"), and swapped "journal prestige" for "journal influence" so the AIS is described the same way every time. ("Outperformed" is retained only where it describes model fit of the negative binomial versus the Poisson specification, which is not an OA comparison.)

Where: throughout.

10. Please distinguish between three layers — unadjusted descriptive findings, primary adjusted findings, and sensitivity.

Thank you for bringing this point to our attention. The layers are defined in the methods, signposted in the results, and carried into the supplement.

The new "Analytic framework" section defines them. Layer 1 is the unadjusted descriptive work, which "describes what is published under each OA label in this corpus and is not adjusted for confounding". Layer 2 is "a single prespecified entropy-balanced negative binomial GLMM, which is the source of every inferential conclusion reported in the Abstract and Conclusion". Layer 3 is the sensitivity analyses, which "vary one modelling decision at a time around the Layer 2 model … and are not used to generate new claims".

The Results now carry three headings to match, and each descriptive paragraph describes the unadjusted citation comparison, for instance, ends "These are unadjusted descriptive comparisons and are not used for inference". The abstract states the structure up front. S5 Table and S3 Fig line up all nine specifications side by side with the primary one marked, so it is obvious at a glance which findings are robust and which are not.

Across all nine specifications, Gold/hybrid OA and Green OA having higher citation rates than Closed Access was stable. Bronze OA versus Closed Access was not: 1.09 in the primary model, 1.05 and non-significant under 95th-percentile truncation, and 0.93, the other direction, with no weighting at all. We removed the directional claim about Bronze from the Discussion and the Conclusion and replaced it with a statement that the direction depends on the specification.

Where: Abstract; Methods, "Analytic framework"; Results, three new layer headings plus a five-paragraph "Layer 3" section; Discussion; Conclusion; S5 Table, S3 Fig.

A few other things we changed

Confidence intervals on the post hoc comparisons. While building the sensitivity tables we noticed the post hoc CIs were computed with an unadjusted critical value while the p-values next to them were Tukey-adjusted. They now both come from the Tukey-adjusted contrasts. Point estimates and p-values are unchanged; the intervals are a bit wider (Bronze OA versus Closed Access is 1.02-1.16 rather than 1.04-1.14), and nothing about the conclusions changes. One comparison had also been printed with a missing upper limit, which we fixed.

A directional contradiction in the Discussion. The first paragraph of the Discussion had retained a definitive statement that Closed Access received 8% fewer citations than Bronze OA (rate ratio 0.92), which contradicted the specification-dependent conclusion stated in the abstract, Layer 3, and Conclusion. We rewrote that sentence so the Discussion, like the rest of the paper, declines to assign a direction to the Bronze OA versus Closed Access comparison.

Mis-referenced tables and incomplete sentences in Layer 1. Two Layer 1 cross-references pointed to the wrong tables (the OA-definitions table and the journal-level table) and three sentences had been left incomplete during earlier restructuring, the unadjusted pairwise-test sentence, the articles-only sentence preceding Table 4, and the Fig 1 caption. All cross-references now point to the correct descriptive/composition tables, and all three sentences are complete.

Figure captions aligned to figure content. The Fig 2 caption referred to stratification "by document type" that did not match the figure as plotted; the caption now matches the figure. The Fig 1 caption now describes both panels (A: citation distribution by category; B: document-type composition).

Two Results paragraphs that contradicted each other. Two consecutive paragraphs in "Reconciliation with the adjusted model" were leftovers from an earlier section order — the first referred in the future tense to model results "reported above" that actually appear below, and the second said the opposite of the first. Merged into one paragraph that makes sense.

Small grammar fixes, including an incomplete sentence in the Methods ("Definitions of each OA category, as classified in Web of Science (Table 1)." now reads "Definitions of each OA category, as classified in Web of Science, are given in Table 1.").

Supporting Information list. It previously listed only two items and did not match what was actually cited in the text. It now lists everything the paper refers to: three figures, five tables, and the data file. Related results are grouped into lettered panels inside a single table rather than split across separate files, so the supplement stays manageable. The standardised-residual table kept its existing S3 Table label so the in-text cross-reference still works.

Thank you again for your review and time with this manuscript. We hope we can publish this manuscript soon.

Kind regards,

Michelle Cruickshank, on behalf of all authors

Attachment Submitted filename: Response to Reviewers - SEPT26.docx

# Editor report: Decision Letter 4

**DOI:** 10.1371/journal.pone.0360532.r009

**Authors:** Amitava Mukherjee

29 Sep 2026

Open access route and citation impact in orthopaedic research: a comparison of Gold/hybrid, Green, and Bronze open access within ten high-impact subscription-based journals

PONE-D-25-46173R4

Dear Dr. Cruickshank,

We’re pleased to inform you that your manuscript has been judged scientifically suitable for publication and will be formally accepted for publication once it meets all outstanding technical requirements.

Within one week, you’ll receive an e-mail detailing the required amendments. When these have been addressed, you’ll receive a formal acceptance letter and your manuscript will be scheduled for publication.

An invoice will be generated when your article is formally accepted. Please note, if your institution has a publishing partnership with PLOS and your article meets the relevant criteria, all or part of your publication costs will be covered. Please make sure your user information is up-to-date by logging into Editorial Manager at Editorial Manager® and clicking the ‘Update My Information' link at the top of the page. For questions related to billing, please contact billing support.

If your institution or institutions have a press office, please notify them about your upcoming paper to help maximize its impact. If they’ll be preparing press materials, please inform our press team as soon as possible -- no later than 48 hours after receiving the formal acceptance. Your manuscript will remain under strict press embargo until 2 pm Eastern Time on the date of publication. For more information, please contact onepress@plos.org.

Kind regards,

Amitava Mukherjee, ME, Ph.D.

Academic Editor

PLOS One

Additional Editor Comments (optional):

Reviewers' comments:

# Editor report: Acceptance letter

**DOI:** 10.1371/journal.pone.0360532.r010

**Authors:** Amitava Mukherjee

PONE-D-25-46173R4

PLOS One

Dear Dr. Cruickshank,

I'm pleased to inform you that your manuscript has been deemed suitable for publication in PLOS One. Congratulations! Your manuscript is now being handed over to our production team.

At this stage, our production department will prepare your paper for publication. This includes ensuring the following:

* All references, tables, and figures are properly cited

* All relevant supporting information is included in the manuscript submission,

* There are no issues that prevent the paper from being properly typeset

You will receive further instructions from the production team, including instructions on how to review your proof when it is ready. Please keep in mind that we are working through a large volume of accepted articles, so please give us a few days to review your paper and let you know the next and final steps.

Lastly, if your institution or institutions have a press office, please let them know about your upcoming paper now to help maximize its impact. If they'll be preparing press materials, please inform our press team within the next 48 hours. Your manuscript will remain under strict press embargo until 2 pm Eastern Time on the date of publication. For more information, please contact onepress@plos.org.

You will receive an invoice from PLOS for your publication fee after your manuscript has reached the completed accept phase. If you receive an email requesting payment before acceptance or for any other service, this may be a phishing scheme. Learn how to identify phishing emails and protect your accounts at https://explore.plos.org/phishing.

If we can help with anything else, please email us at customercare@plos.org.

Thank you for submitting your work to PLOS One and supporting open access.

Kind regards,

PLOS One Editorial Office Staff

on behalf of

Professor Dr. Amitava Mukherjee

Academic Editor

PLOS One
